# Machine-Learning-Based Prediction of the Glass Transition Temperature of Organic Compounds Using Experimental Data

**DOI:** 10.1021/acsomega.2c08146

**Published:** 2023-03-22

**Authors:** Gianluca Armeli, Jan-Hendrik Peters, Thomas Koop

**Affiliations:** Faculty of Chemistry, Bielefeld University, 33615 Bielefeld, Germany

## Abstract

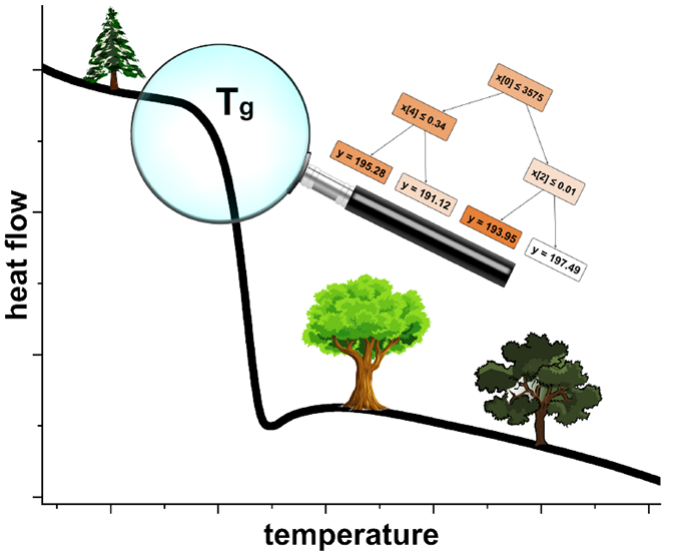

Knowledge of the glass transition temperature of molecular compounds that occur in atmospheric aerosol particles is important for estimating their viscosity, as it directly influences the kinetics of chemical reactions and particle phase state. While there is a great diversity of organic compounds present in aerosol particles, for only a minor fraction of them experimental glass transition temperatures are known. Therefore, we have developed a machine learning model designed to predict the glass transition temperature of organic molecular compounds based on molecule-derived input variables. The *extremely randomized trees (extra trees)* procedure was chosen for this purpose. Two approaches using different sets of input variables were followed. The first one uses the number of selected functional groups present in the compound, while the second one generates descriptors from a SMILES (Simplified Molecular Input Line Entry System) string. Organic compounds containing carbon, hydrogen, oxygen, nitrogen, and halogen atoms are included. For improved results, both approaches can be combined with the melting temperature of the compound as an additional input variable. The results show that the predictions of both approaches show a similar mean absolute error of about 12–13 K, with the SMILES-based predictions performing slightly better. In general, the model shows good predictive power considering the diversity of the experimental input data. Furthermore, we also show that its performance exceeds that of previous parameterizations developed for this purpose and also performs better than existing machine learning models. In order to provide user-friendly versions of the model for applications, we have developed a web site where the model can be run by interested scientists via a web-based interface without prior technical knowledge. We also provide Python code of the model. Additionally, all experimental input data are provided in form of the Bielefeld Molecular Organic Glasses (BIMOG) database. We believe that this model is a powerful tool for many applications in atmospheric aerosol science and material science.

## 1 Introduction

The glass transition is a nonequilibrium phase transition. Kinetically, the glass transition temperature *T*_g_ is defined as the temperature at which the viscosity of a substance reaches a value of about 10^12^ Pa s. This transition from a liquid to an amorphous solid state is accompanied by a change in heat capacity that can be detected experimentally, e.g., by differential scanning calorimetry.^1^ Due to its nonequilibrium nature, the glass transition temperature depends on the thermal history of the material, thereby impeding the comparison of experimental *T*_g_ values and their theoretical prediction.^2^ Over the years, several surrogate methods have been developed for predicting the conditions for a glassy state and its transition temperature for molecular organic compounds.^3−5^ A long-known and simple, but surprisingly reliable, method is the Boyer–Beaman rule^6,7^ that formulates a proportional relationship between the glass transition temperature *T*_g_ and the melting temperature *T*_m_ of a substance. Further studies have elaborated that the *T*_g_/*T*_m_ ratio is ∼0.7 for a large variety of substances.^8^ Nevertheless, this approach is sometimes considered to be only a rule of thumb, in light of the statistical deviation of the various data from the prediction (1σ: ±21 K, 2σ: ±42 K).^8^

When it comes to theoretical frameworks that function based on historical data, so-called data-centric models, machine learning algorithms are the state-of-the-art tools. These experience-driven models have already shown to yield promising results in context of predicting the glass transition temperatures of inorganic oxide glasses;^9,10^ however, only very few attempts have been made to apply them to molecular organic compounds.^11,12^ The latter compounds are of great interest for atmospheric science, because they exist in the atmosphere, for example as components in organic aerosol particles.^13^ Moreover, the glass transition temperature is crucial for our understanding of organic aerosols, as it affects, for example, cloud formation processes or diffusion-dependent in-particle processes during gas uptake and chemical reactions.^14,15^

Within the past decade, the application of machine learning methods in natural sciences has experienced a rapid growth. Most models that were developed in this context are used either for the discovery of new systems^16,17^ or for the prediction of a specific property.^18−22^ In 2014, Alzghoul et al.^11^ published a study in which machine learning algorithms (mainly support vector machines and neural networks) were used to predict the glass transition temperature of organic molecular compounds. One limitation of their work is that only a rather small data set of 71 druglike substances (mostly functionalized heterocycles) was considered. Thus, their model is only applicable to this specific group of substances, which lacks the representation of more common and simple molecules. In addition, no possibility to access and use the model for further calculations was provided. Tao et al.^23^ investigated the performance of over 70 different machine learning models on a polymer *T*_g_ data set and observed that a *random forest* algorithm performed best.^23^ Even more recently, Galeazzo and Shiraiwa^12^ introduced a “tgBoost” model for *T*_g_ predictions, which was built on a larger experimental data set of 298 substances (mainly obtained from Koop et al.^8^). This model employs *extreme gradient boost regression* along with molecular descriptors based on the SMILES notation to predict the *T*_g_ of organic molecules. The model code is accessible through a public repository, thereby allowing the implementation in future research activities. We note that its application requires prior knowledge on the installation and usage of Python (and its packages) as well as other prerequisites, which may be a drawback to some researchers unfamiliar with these tools.

In the work described in this article, we present several variants of a machine learning model that achieve an improved performance by combining molecular descriptors and melting temperature. The model operates on an *extremely randomized trees (extra trees)* algorithm that is substantially different from the algorithms used in the previous models. Furthermore, we provide an online version of the model in the form of a web site, thus ensuring a user-friendly environment where everyone can use the model without any prior technical knowledge to predict *T*_g_ of organic compounds. We also provide Python code for model applications through a public repository.

## 2 Methods

### 2.1 Machine Learning

In general, machine learning (ML) aims at training an algorithm to solve a specific problem given the independent *x*-values (features) and the corresponding *y*-values (labels) of a predefined data set.^24^ ML is used to describe complex multivariant dependencies beyond the capabilities of conventional fitting methods. In case the output data is categorical, i.e., it can only adopt discrete values, for example, the categories of “cats” and “dogs” in image classification, a classification problem is faced. If the label is continuous, as in our case the glass transition temperature *T*_g_, then a regression problem needs to be solved. In both cases, the model should have learned about the data set’s patterns and dependencies in a way that it is able to predict the yet unknown output label for a set of input features that was not included in the original training data set.

#### 2.1.1 Decision Tree Based Algorithms

The algorithm that performed best in our study uses a *decision tree*(25) as its estimator. Resembling a real tree, in a *decision tree* multiple nodes are connected by branches. The final nodes are called leaf nodes, while all the others are termed decision nodes. The very first node (root node) contains the whole data set. At each decision node, a feature-specific statement checks whether or not a sample exceeds a certain threshold (e.g., *X*_1_ ≥ 2). Such decisions split the data set and pass the splits (children) to the following nodes. In the case of a regression problem, the best split is chosen by maximizing the variance reduction of the parent and child nodes, i.e., by creating child nodes that have lower variances than their parent. Ideally, this procedure should result in low-variance leaf nodes, without overfitting the tree, i.e., strictly memorizing the training data and losing the ability to generalize. This tree-development procedure is controlled by various parameters such as the depth of the tree, i.e., the number of layers. Finally, the predicted value is obtained by forming the mean of all the values in one leaf.

The *random forest* algorithm^26^ consists of multiple individual *decision tree*s and is, therefore, an ensemble method.^27^ Such an ensemble of trees is more powerful than a single *decision tree*, because of the *uncorrelatedness* of the different trees. The trees become uncorrelated by assigning each tree a different subset of the data instead of providing each tree the entire data set. The subset is randomly picked from the entire data set with replacement, which allows the subset to contain duplicate samples. This method is known as *bootstrapping*. Another way to uncorrelate trees is the *random subspace* method, where instead of giving each tree access to all the features, each tree can only use a random subset of features. Each tree will then make an individual prediction and subsequent averaging over the predictions of all the trees’ results in the forest’s final output.

The *extra trees* algorithm^28^ relies on the same concept as the *random forest*, but has two major differences. First, the subsets are created without replacement and, second, instead of searching for the best split by maximizing the variance reduction, only a few random splits are considered and the best one among those is chosen. Generally, the *random forest* and *extra trees* algorithm are believed to perform equally well, but *extra trees* seems to outperform *random forest* when noisy features are present.^29^

Another advantage of the algorithms described above is that they represent “explainable” ML algorithms. For some time, ML was criticized for being mainly used as a black box tool, as algorithms got too complex to be understood by users of other fields, as supported by a recent online survey.^24^ However, the principles of decision trees are relatively easy to understand and with some further research more profound knowledge can be gained. It is possible to inspect individual nodes and trees in order to follow the work flow, thereby enhancing the reproducibility and confirmability.

#### 2.1.2 Training and Test Sets

In contrast to conventional fitting techniques, employing the whole data set to train a model is not recommended for ML applications, because more sophisticated algorithms can become so efficient at predicting the training data that no reasonable assessment of the model’s performance is possible anymore. For this reason, a minor fraction of the data set should be set aside for testing the model appropriately and typically 80–90% of the data are used for training.^9,10^ In the current study, 90% of the data were used to train the model, whereas 10% were used to test it afterward.

The actual splitting of the data into a training and a test set was done randomly using a pseudorandom number generator (PRNG).^30^ The PRNG is initiated with a seed, which is an arbitrarily chosen number. According to the seed, a set of random numbers is generated. A specific seed will always create the identical set of random numbers, which is important when it comes to comparing the results of different training runs. If a true random number generator was used at this point, then there would be splits with different samples for training and test data in each run, which would not allow for a comparison of the different runs. The PRNG makes it possible to control the randomness and receive reproducible results of data set splits.

An important step in model development is to choose an appropriate ML algorithm. The above explanation underlines that the performance of any algorithm will depend on the randomly assigned training and test sets. However, when looking for a more general statement of how an algorithm will perform “in the wild” on unseen data, different splits of training and test sets have to be explored. This is typically achieved by the execution of the so-called *k-fold cross-validation*.^31,32^ In this method, *k* is an integer number that represents the number of equally large segments, into which the data set is split. Thereafter, a total of *k* runs will be performed. For every run, one of the *k* data segments will be used as a test set, while the other segments are combined and used as a training set. The model performance is then assessed by a metric score, e.g., the mean absolute error. This procedure is repeated, but each time with a different data segment as the test set. Finally, after completing *k* runs, each data segment was used once as the test set and *k* – 1 times in the training set (see Figure S1 for illustration). The cross-validation score is obtained by calculating the mean of the *k* individual metric scores of each run. In this work, a 10-fold cross-validation (*k* = 10) was applied to investigate, which of the algorithms performs best among the various ones tested. The metric we chose for our evaluation is the mean absolute error (MAE):
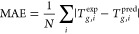
1

Here,  is the predicted glass transition temperature value of compound *i*,  is the experimental *T*_g_ value of compound *i* and *N* is the number of samples in the test set.

### 2.2 Feature Representations

The objective of this work was the robust prediction of the glass transition temperature of organic substances with a low MAE, implying that *T*_g_ is the label. As features, numerical forms of molecular descriptors are required that characterize a particular substance distinctly. These kinds of vector representations are called fingerprints^33^ or feature vectors. For our purposes, we developed several different approaches, which we term modes, and we examine each of these modes in detail below.

#### 2.2.1 Functional Group Mode

The first approach builds on a series of chemical functional groups and uses the number of appearance in the molecule of each of these functional groups as well as other molecular properties to build the feature vector used by the algorithm. Working with functional groups is a principle that was already applied successfully by numerous previous works, for example, in UNIFAC-type models for describing thermodynamic properties in multicomponent liquid mixtures.^34−36^ Such functional group-based features can be divided into direct inputs, which users enter by themselves, and indirect inputs that are calculated autonomously by the model. The functional groups considered in our model are methyl (CH_3_), methylene (CH_2_), methine (CH), carbon atoms that are not bonded to hydrogen atoms (C), hydroxyl (OH), ether oxygen (−O−), carbonyl oxygen (=O), nitrogen atoms (N), and halogens (Hal). Additionally, the following features were included: The atomic oxygen-to-carbon (O/C) ratio of the molecule, its molar mass (*M*), and the double-bond equivalent (DBE) calculated according to the following equation:
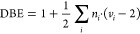
2where *n*_*i*_ is the number of atoms present in the molecule and *v*_*i*_ is their valence, defined as an atom’s number of regular bonding partners.^37^ Finally, the melting temperature *T*_m_ can also be included as an optional feature, see Table 1 for an overview of all the features used here. Altogether, these features form a feature vector that contains the molecular information and that can be passed into a ML algorithm. We chose these particular functional groups in order to describe a molecule as precisely as possible, while at the same time ensuring a relatively user-friendly input. While this feature vector representation still has some limitations (see section 2.3 below), it is much more distinct than just simply using the chemical formula, for example.

**Table 1:** Molecular Features and Their Description

feature	type of input	description
CH_3_	direct	number of methyl groups
CH_2_	direct	number of methylene groups
CH	direct	number of methine groups
C	direct	number of carbon atoms not bonded to hydrogen atoms
OH	direct	number of hydroxyl groups
–O–	direct	number of ether groups
=O	direct	number of double-bonded oxygen atoms
N	direct	number of nitrogen atoms
Hal	direct	number of halogen atoms
DBE	direct	double bond equivalent
O/C	indirect^a^	atomic oxygen-to-carbon ratio
*M*	indirect^a^	molar mass in g mol^–1^
*T*_m_	optional	melting temperature in K

a Calculated internally by the model from the direct input features.

#### 2.2.2 SMILES Mode

**Figure 1:**
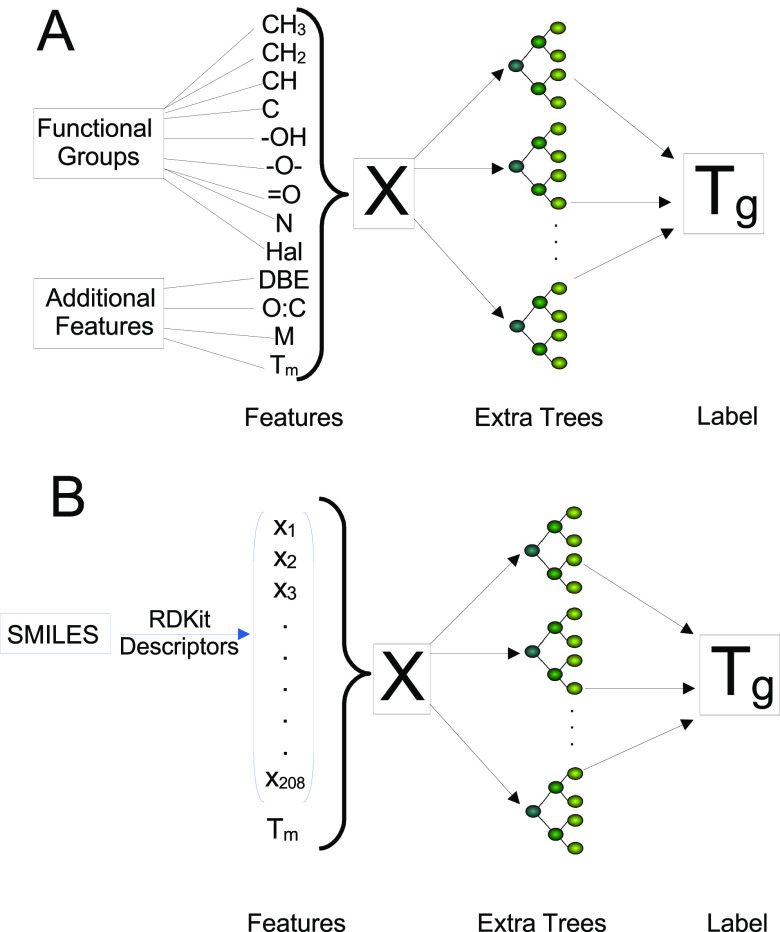
Schematic representation of the work flow of each mode. The Functional Group Mode (A) uses functional group attributes and some additional features, while the SMILES Mode (B) uses molecular descriptors from a SMILES string. Once the feature vector is generated, both models pass it to an *extra trees* regressor for *T*_g_ prediction.

The second approach used in this study employs a SMILES string as an input. SMILES is the abbreviation for *Simplified Molecular Input Line Entry System*, which is a widely used method for capturing the chemical structure of a molecule as a series of characters, a so-called string. The SMILES string of a particular molecule is transformed into a fingerprint by a so-called featurizer. In the Python programming language, the RDKit package^38^ is required to handle SMILES strings. Along with the DeepChem package,^39^ the featurizer RDKitDescriptors is used here. It generates a 208-bit vector, in which every bit refers to a certain molecular property or structural information. One restriction of this kind of representation is that stereochemistry can not be resolved, even when formally declared in the SMILES string, because different stereoisomers result in the same RDKitDescriptors vector. Since the stereo information would not have any benefit, it was therefore neglected in the SMILES code, thus using the canonical SMILES string that does not consider stereo configuration. We note that the loss of stereo information is only a minor drawback at this point, because for the evaluated samples, the glass transition temperatures of configuration isomers and diastereomers is not expected to differ significantly, as evidenced by exemplary experiments.^40^ Moreover, there are only very few literature sources, in which any clear distinction between such stereoisomers has been made when providing experimental *T*_g_ data of molecular compounds. Finally, also in the SMILES Mode we added the melting temperature as an optional feature, as it turned out that it significantly enhances the performance of ML predictions in this mode, see further details below. A schematic drawing of the work flow in both of the employed modes is depicted in Figure 1.

### 2.3 Data Set

For this work, experimental glass transition temperatures of organic molecular substances were required as a training set. The majority of the data used here was taken from a data set collected by Koop et al.^8^ In that study, a total of 596 *T*_g_ values of various substances were analyzed, of which about 480 originate from organic molecular compounds, and these were used in the current study. The other major data source arises from the supplement of Rothfuss and Petters,^41^ who also collected a very similar type of data set. Note that we carefully looked up each reference and excluded those data, which were already obtained from our first source.^8^ Finally, the data set was enriched with further experimental *T*_g_ data obtained by differential scanning calorimetry measurements in our laboratory.^40,42,60^ It is worth mentioning that we exclusively used experimental data and did not include any theoretically derived *T*_g_ data, for example from molecular dynamics simulations or similar methods. The only exception is that for 20 compounds, for which experimental *T*_g_ data were available, we did not find any experimental *T*_m_ data. Hence, in these cases, the melting temperatures were approximated with help of the Joback method.^43^

All experimental input data used in this study are provided in form of the Bielefeld Molecular Organic Glasses (BIMOG) database in the Supporting Information and in a public data repository (DOI: 10.5281/zenodo.7319485).

The molecular compounds in our data set consist primarily of C–, H–, O–, N–, and halogen atoms. There are very few compounds with sulfur atoms, but they are so underrepresented in the data set that we do not recommend to use the model for such compounds. The same is true for relatively large molecules (>600 g mol^–1^): While there are some compounds in the data set with larger molar mass, there are too few to justify a generalization. One problem that arose at the early data assessment stage was that quite often multiple experimental *T*_g_ data are available for the same substance, originating from different literature sources, methods or experimental conditions, e.g., cooling and heating rate. In a previous study on the prediction of glass transition temperatures of polymers with a ML algorithm, this issue was addressed already.^44^ That study revealed that in all cases analyzed, using the median of the glass transition temperatures of multiple-reported values leads to best results as measured by standard metrics such as the root mean squared error. For this reason, the same approach was followed in this work when encountering such “duplicate” samples. A detailed description of the data preprocessing can be found in the Supporting Information.

One drawback of the feature representations described above is that they are not always capable of describing a molecule unambiguously. For example, in a few cases either the constitution of the molecule or its configuration becomes ambiguous, implying that these originally distinct molecules are represented by the same feature vector; thus, they are treated as the same substance. As discussed above, the configuration is apparently not very important, but the importance of constitution can not be assessed easily. There are cases where the difference in the glass transition temperature of constitutional isomers is only minor, as in the cases of 2-pentanol (*T*_g_ = 140 K) and 3-pentanol (*T*_g_ = 143 K).^45^ On the other hand, there are isomers that differ significantly in their glass transition temperature, for example, sucrose (*T*_g_ = 334 K) and trehalose (*T*_g_ = 385 K).^8^

**Figure 2:**
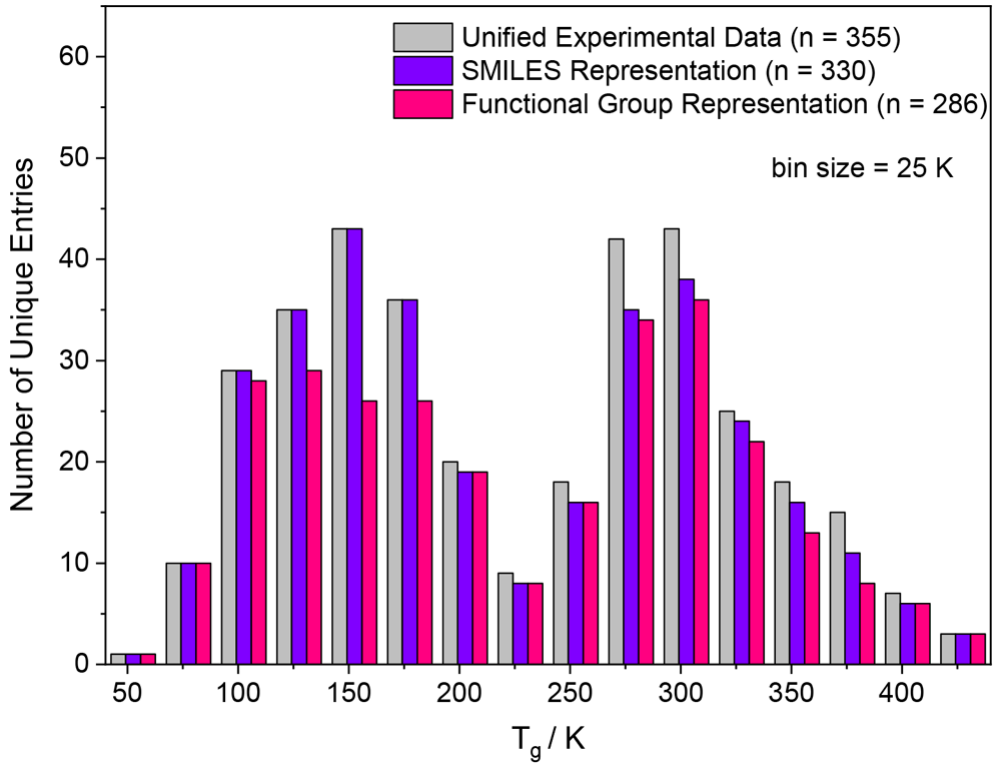
Histogram plot of the glass transition temperature distribution of the unified experimental data set used in this study. Gray bars indicate the number of unique entries, i.e., individual substances, in the data set. Violet and pink bars show the unified data in the feature representation of the SMILES Mode and the Functional Group Mode, respectively. The bin size was set to 25 K (leading to the bins 50–75 K, 75–100 K, etc.) and the bins are separated by blank space.

As a result of these restrictions, the data set shrinks in terms of unique *x*-values and at the same time there are multiple *y*-values for some *x*-values. Figure 2 visualizes this effect by showing the unified experimental data used in this study (gray bars) consisting of 355 entries. Applying the SMILES Mode feature representation reduces the original data to 330 unique entries (violet bars), because the RDKit descriptors do not resolve the stereo configuration, which is the information that gets compromised at this point. The Functional Group Mode feature representation is less powerful at providing a unique definition of a particular molecule, and, thus, only 286 unique entries remain for this representation (pink bars). Generally, a continuous uniform distribution of the label input data would be desired here to allow for a balanced representation over the entire data range, and any strongly unbalanced distribution would lead to an over-representation of the label in the data range close to the maximum. The distribution in Figure 2 has some gaps at the ends and in the middle, but overall it is relatively balanced. We note that the size of the data set is rather small compared to typical ML applications, but previous studies have already shown that also small data sets can yield promising outcomes.^46,47^

**Figure 3:**
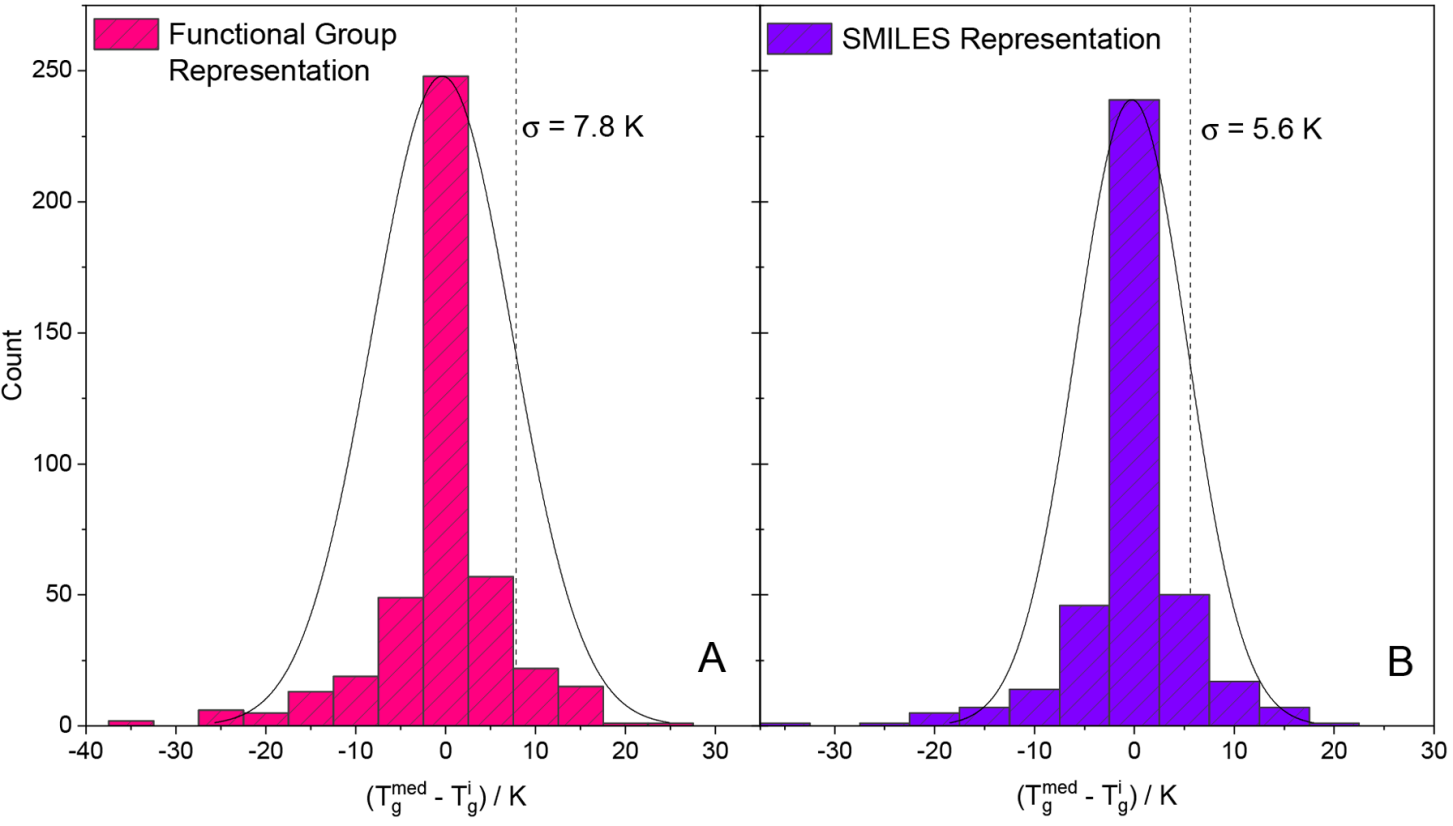
Histogram with normal distribution fit of the duplicates’ variation from their median for the Functional Group Representation (A) and the SMILES Representation (B). For all multiple values belonging to one feature vector the median was calculated and then the difference between this median and the individual values was obtained. The fits yielded standard deviations of ±7.8 K and ±5.6 K, respectively.

Another interesting point concerning the duplicate feature values can be seen in Figure 3, in which the deviation of the individual duplicate glass transition temperature values  (all belonging to one feature vector) from their median value  is depicted. As noted above, such duplicated values originate because the same substance was actually measured multiple times or because the feature representation of different substances is the same. For the Functional Group Mode (Figure 3A) as well as the SMILES Mode (Figure 3B), the data obey a normal distribution centered around the zero value, most likely due to the fact that most of the values are doublets. Often, these doublets come from the same research groups which measured a substance twice and/or reported the identical value in two different articles published a few years apart from each other. The distributions shown in Figure 3 thus support the procedure described above of using the median *T*_g_ value for such doublets.

### 2.4 Parametrizations for the Prediction of *T*_g_

To compare the predictive power of the ML models developed in this work with well-established literature methods, three alternative models were used for comparison: First, the Boyer–Beaman rule^6,7,48^ (eq 3), which predicts *T*_g_ based on the melting temperature *T*_m_ of a substance:
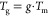
3Here, *T*_m_ is the melting temperature and *g* is a constant, whose value was found to be approximately 0.7 (1σ: ±21 K, 2σ: ±42 K) according to a previous analysis.^8^ Second, we used the parametrization by Shiraiwa et al.^3^ (eq 4), which is based on the molar mass and atomic oxygen-to-carbon (O/C) ratio of organic compounds:
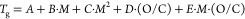
4Here, *A*, *B*, *C*, *D*, and *E* are fit parameters with the following values: *A* = −21.57 (±13.47) [K], *B* = 1.51 (±0.14) [K mol g^–1^], *C* = 1.7 × 10^–3^ (±3 × 10^–4^) [K mol^2^ g^–2^], *D* = 131.4 (±16.01) [K], and *E* = −0.25 (±0.085) [K mol g^–1^]. *M* is the molar mass and (O/C) the atomic O/C-ratio. And third, we used the parametrization by DeRieux et al.^4^ (eq 5), which is based on the molecular formula of a compound:
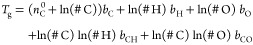
5Here, #C, #H, and #O are the number of carbon, hydrogen and oxygen atoms present in the compound. The values of all the other parameters are given in Table 2. We note that the parametrizations presented in eqs 4 and 5 were designed for compounds consisting only of C, H, and O atoms, i.e., CH and CHO compounds.

**Table 2:** Parameter Values for eq 5 Depending on the Substance Class^4^

classes	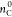	*b*_C_	*b*_H_	*b*_CH_	*b*_O_	*b*_CO_
CH	1.96	61.99	–113.33	28.74	0	0
CHO	12.13	10.95	–41.82	21.61	118.96	–24.38

More recently, Li et al.^5^ provided a parametrization for calculating the glass temperature of CHON compounds that also include nitrogen (N) atoms:
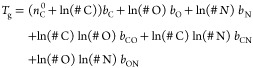
6with the parameter values  = 5.24, *b*_C_ = 31.53, *b*_O_ = −7.06, *b*_N_ = 134.96, *b*_CO_ = 6.54, *b*_CN_ = −34.36, and *b*_ON_ = −15.35. The *#* symbol refers once again to the number of the specified atom in the compound.

## 3 Results and Discussion

### 3.1 Feature Importance in the Functional Group Mode

Initially, we applied different decision tree-based algorithms to a subset of the data that contained only CH and CHO compounds, in order to allow for a better and fair comparison to the literature parametrizations (eqs 3–5) that were only designed for this group of compounds. From now on, we will refer to this subset as the “CHO data set”.

**Figure 4:**
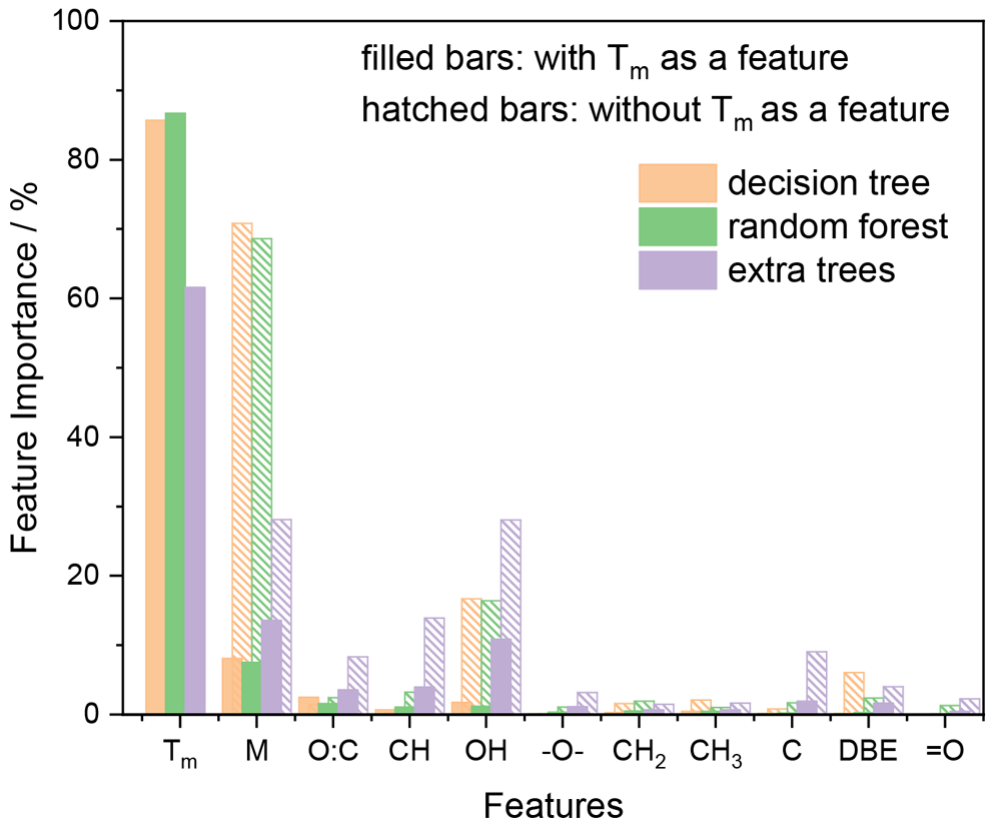
Illustration of the relative feature importance within decision tree-based algorithms applied to the representation of the Functional Group Mode using the CHO data set.

Figure 4 illustrates how important the analyzed algorithms consider each feature of the Functional Group Mode for the CHO data set. These values were obtained by claiming the algorithms’ intrinsic attribute “feature importance” and were computed as the normalized total variance reduction. As already explained in section 2.1.1, the estimators in the *random forest* (green bars) and *extra trees* (purple bars) do not necessarily get full access to all the available features/samples, when compared to a single *decision tree* (orange bars). The *random forest* estimators then search for the best split among all possible splits.

All three algorithms have in common that the top three features are the melting temperature *T*_m_, the molar mass *M*, and one of the oxygen-related features such as OH or O/C, some of which have already been identified as key parameters for the glass transition temperature previously.^3−5,8,41^ This observation is consistent with the fact that they have a high correlation with *T*_g_ and have been used already in previous parametrizations, i.e., eqs 3–5. While the observation that *T*_m_, *M*, OH, and O/C are the most important features is not surprising and not a new result in general, it nevertheless corroborates that also the ML model automatically identifies these strong correlations of physical significance. One may argue, however, that including indirect features such as *T*_m_, *M*, or O/C (see Table 1) is an unphysical self-fulfilling prophecy. Therefore, we also ran an *extra trees* model that was trained without the *T*_m_, *M*, and O/C features and analyzed its feature importances. Those values were then compared to the original model that was trained with all the features, but had the feature importance for *T*_m_, *M*, and O/C removed and subsequently renormalized the remaining relative importance feature values (see Figure S3). That comparison indicates that the removal of the features does not fundamentally change the model and only very slightly reduces its predictive power (MAE of 13.1 K is increased to 14.1 K when the indirect features were removed). Moreover, the relative feature importance distribution among the functional groups is hardly affected, with the number of OH-groups being the most important feature (see Figure S3 and related discussion). This outcome can be explained by the fact that OH-groups contribute to the formation of intermolecular hydrogen bonds, which are an essential property contributing to the viscosity and, hence, also the glass transition temperature of organic compounds, as noted previously.^8,41^

In conclusion, the feature importance comparisons of Figures 4 and S3 reveal that the underlying physical chemistry is not compromised by including the indirect features *T*_m_, *M*, and O/C into the Functional Group Model, but very slightly improves its predictive power.

In addition, we checked whether our model produces a physically meaningful behavior when predicting *T*_g_ of various chemical compound classes such as *n*-alkanes, *n*-alcohols, diols and triols as a function of the number of carbon atoms of the molecule (see Figure S4). We observed that *T*_g_ generally increases slightly with the number of carbon atoms within a compound class, and *T*_g_ strongly increases with the number of OH-groups at constant number of carbon atoms, consistent with the results of prior studies.^12,41^

We also considered the problem of multicollinearity caused by quasi redundant features, which is a known problem in ML.^49,50^ For that purpose, we calculated a correlation matrix for the feature–feature correlations. Features are considered as highly correlated when their correlation coefficient is greater than 0.9,^49,50^ however, that was not the case for any of the features (see Figure S2 and related text).

As mentioned above, the *k*-fold cross-validation is an effective method for evaluating different training and test sets and the mean absolute error (MAE) is an appropriate metric for comparing different models or algorithms. The comparison of the three Functional Group Mode algorithms in a 10-fold cross-validation revealed that the *extra trees* regressor reached the best score, i.e., the lowest MAE compared to the *random forest* and *decision tree* algorithm. For this reason, we chose the *extra trees* regressor algorithm as the core of our ML models described in more detail below.

### 3.2 Functional Group Mode

**Figure 5:**
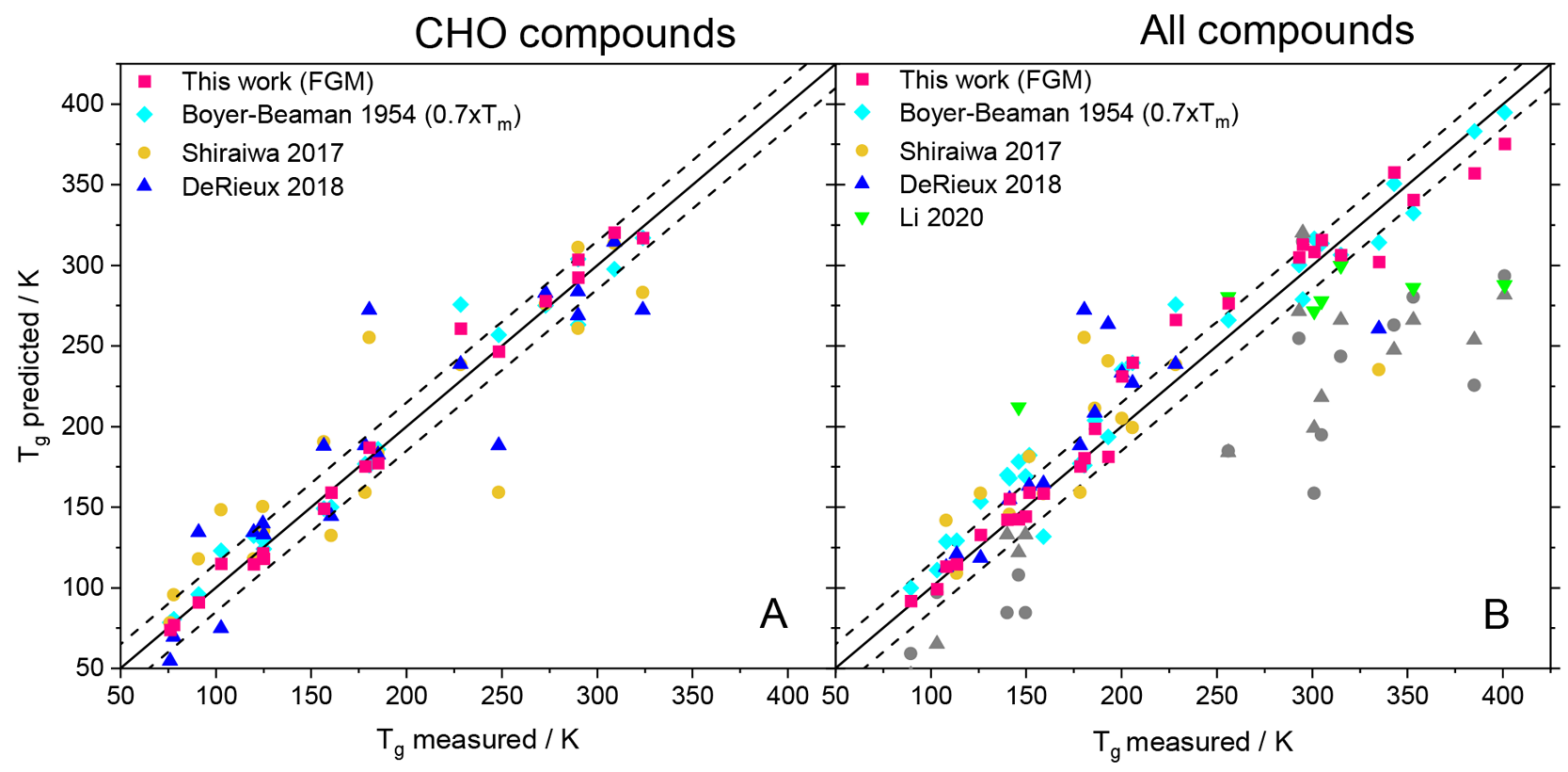
Predicted *T*_g_ versus experimental *T*_g_ values. Panel A contains samples from the CHO test set and panel B that of the NHal-extended test set. The predictions of the Functional Group Mode (magenta squares) are compared with predictions of various parametrization-based models from the literature: The Boyer–Beaman rule^6^ (cyan diamonds), Shiraiwa et al.^3^ (yellow circles), DeRieux et al.^4^ (blue triangles), and Li et al.^5^ (green triangles). Gray masked symbols originate from nitrogen or halogen compounds and were calculated with the Shiraiwa or DeRieux formula, which shall be applied to CHO compounds only. The dashed lines indicate a deviation of ±15 K from the 1:1 line (solid line).

Figure 5 shows the *T*_g_ values predicted with the Functional Group Mode (FGM) versus the experimental *T*_g_ data. The line of unity (solid black line) represents a perfect prediction and the dashed lines indicate deviations of ±15 K from the perfect centerline. In Figure 5 we compare the results of our ML model operating on *extra trees* regression (magenta squares) with those of the previously introduced literature parametrizations (cyan diamonds, yellow circles, and blue triangles) on randomly picked test sets. We note that the data of the test sets were not included in the training of our ML models. Panel A shows the predictions of Functional Group Mode on the CHO test set, and panel B shows those on the NHal-extended test set. At first glance, the magenta squares (ML model) are closer to the line of unity than any of the other types of symbols, indicating a good description of the experimental data by our model. In both panels, only a few outliers are observed, which are not located within the corridor of ±15 K of the two dashed lines. Another point worth mentioning is that even though the melting temperature is the most important feature, there are a few samples where the predicted value from the Boyer–Beaman rule (cyan diamonds) is significantly off the center line, while the magenta square of the ML prediction is closer to the experimental value. This behavior implies that the ML models do not solely rely on the melting point dependency but also take other features into account and thereby adjust their weights for different chemical structures. In the discussion of Figure 4 above we pointed out that *T*_m_ is the most important feature in the ML models, which seems reasonable given its very high correlation with *T*_g_, as evidenced by the Boyer–Beaman rule (eq 3). However, a *T*_m_ value may not be available for every substance for which the prediction of *T*_g_ is desired. Therefore, we trained two ML model variants, one with and one without the melting temperature as a feature; see more detailed discussion below.

Furthermore, to make a meaningful comparison of the models, a *nested cross-validation* was performed. This evaluation method gives a reliable estimate of how a model performs on different test sets. This nested cross-validation (ncv) works just like a normal cross-validation as described in section 2.1.2, but with the difference that for each cv-training set a hyperparameter tuning is conducted. This process returns the best model parameters among a predefined selection of possible parameter values (also known as a *grid search cv*). Thereafter, these best parameters are used to make the prediction on the cv-test set. Once again, the MAE (eq 1) was used as a metric score. Since a nested cross-validation can only be performed for the ML algorithms, we calculated the MAE for the literature parametrizations (eqs 4 and 5) using the entire data set, which we believe is a fair and valid comparison. The resulting MAEs are given in Table 3. As highlighted with bold text, the Functional Group Mode (FGM) reaches the lowest MAE (about 13 K) for both data sets. If the FGM is used without the melting point feature, then its performance drops significantly, but it is still more accurate than those of the previous parametrizations.

**Table 3:** Nncv-MAE of the Functional Group Mode (FGM) Is Compared to the MAE of the Parametrizations on the Whole Data Set in the FGM Feature Representation

data set	FGM(with *T*_m_) ncv-MAE [K]	FGM(no *T*_m_) ncv-MAE [K]	Boyer–Beaman MAE [K]	Shiraiwa MAE [K]	DeRieux MAE [K]
CHO set	**13.1**	16.4	21.2	27.4	22.8
NHal set	**13.0**	19.6	19.1	29.3^a^	25.8^a^

a For CHON compounds, the parametrization from Li et al.^5^ was used; other compounds (e.g., CHN, CHHal, etc.) were not considered.

### 3.3 SMILES Mode

**Figure 6:**
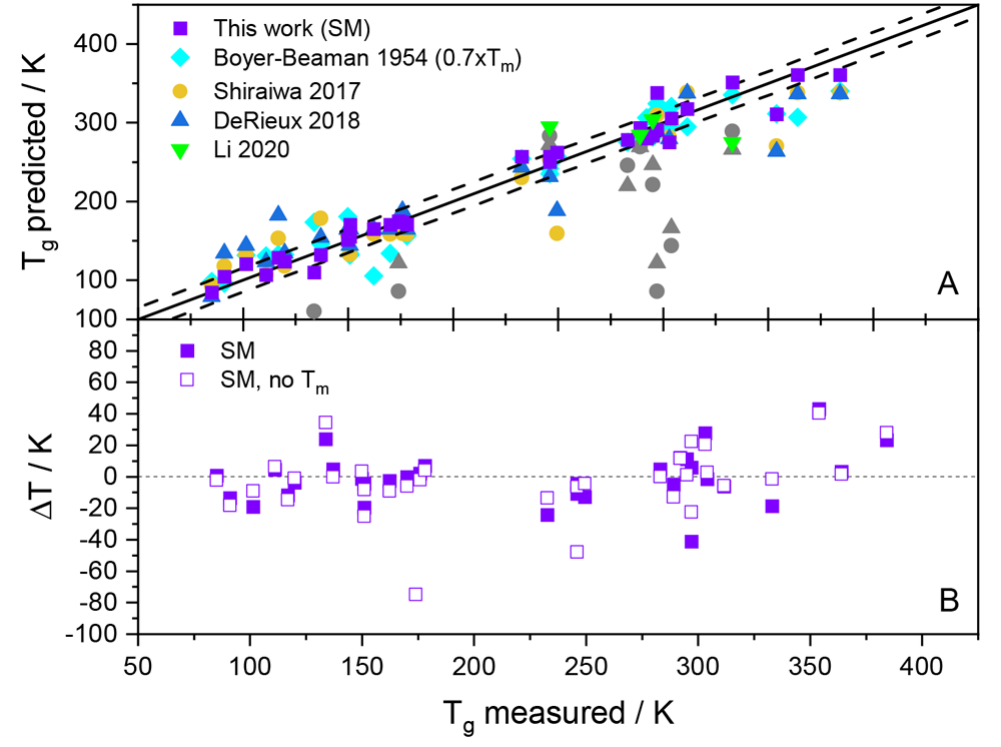
Predicted *T*_g_ values plotted against experimental *T*_g_ values (panel A). Purple squares are predictions using the SMILES Mode of the model, all other symbols are shown as in Figure 5. Gray masked symbols pertain to nitrogen or halogen compounds and were calculated with the Shiraiwa or DeRieux formulas (eqs 4 and 5), which shall be applied to CHO compounds only. In panel B the difference between the experimental and the predicted *T*_g_ value is shown for the SMILES Mode with the melting temperature as a feature (filled squares) and without it (open squares).

In this section, we repeat the evaluation of the ML model of the previous section, but this time for the SMILES Mode (SM) feature representation, which is built on a SMILES-based molecular descriptor. The unified version of the CHONHal data set within this feature space contains 330 unique entries, 44 more than that of the FGM model. In Figure 6A the *T*_g_ predictions of the SM are plotted against the experimental *T*_g_ literature values. In Figure 6B, the difference between the experimental and the predicted value, i.e., , is presented for the ML model predictions with and without the melting point as a feature. Except for a few outliers, the points that correspond to the same molecule do not deviate much from each other, implying that using the SMILES-based descriptor without the *T*_m_ feature still provides very reasonable prediction results. We note a few data points, where the no-*T*_m_ variant performed slightly better than that including the *T*_m_ feature. Small differences on the order of a few kelvins can be probably attributed to the variance in the algorithm. In our case, the decision trees are built in a greedy fashion, which implies that the algorithm will follow the first path with the lowest variance reduction. This procedure may not necessarily lead to the global minimum, which is why minor changes in building the trees can make a difference. Moreover, there are a few data points that show higher deviation from each other (in both directions), independently of whether the Boyer–Beaman rule works well for these substances or not. Focusing on the two highest deviations in this graph (*ortho*-fluoroaniline and dibucaine, no *T*_m_ mode) it is striking that the Boyer–Beaman rule works moderately well, and seemingly this transferred to the *T*_m_ mode, which is significantly better.

In Figure 6A we compare the predictions of the SM model to the other parametrization methods. Once again the *T*_g_ predictions of the SMILES Mode ML model with *T*_m_ feature (purple squares) show the best overall agreement with the experimental *T*_g_ values. We again performed nested cross-validations yielding MAE values of 11.7 and 15.1 K for the SMILES Mode variants with and without the *T*_m_ feature, respectively (see Table 4). The comparison shows that the *T*_m_ feature significantly improves the performance of the ML model, but even without it the SM model still outperforms the other parametrization-based methods. The Boyer–Beaman rule (cyan diamonds) produced a MAE of 19.7 K. The parametrizations by Shiraiwa^3^ and DeRieux^4^ (yellow circles and blue triangles, respectively) are only suited for CHO compounds, which is why the data points referring to NHal components calculated with these formulas are masked as gray data points for fairness. Green triangles represent *T*_g_ values predicted with the parametrization by Li et al.^5^ (eq 6). Since this parametrization is only valid for CHON substances, not every nitrogen-containing substance in the test set could be considered (e.g., CHN and CHONHal compounds). Overall, the comparison in Figure 6A shows that the SMILES Mode ML model does a very good job in predicting *T*_g_ for a large variety of organic compounds.

**Table 4:** ncv-MAE of the SMILES Mode (SM) Compared to the MAE of the Parameterizations on the Whole (NHal) Data Set in the SM Feature Representation

SM(with *T*_m_) ncv-MAE [K]	SM(no *T*_m_) ncv-MAE [K]	Boyer–Beaman MAE [K]	Shiraiwa MAE [K]	DeRieux MAE [K]
**11.7**	15.1	19.7	27.6^a^	24.2^a^

a For CHON compounds the parametrization from Li et al.^5^ was used; other compounds (e.g., CHN, CHHal, etc.) were not considered.

Finally, Galeazzo and Shiraiwa^12^ very recently introduced the so-called tgBoost ML model for predicting *T*_g_ of organic molecular compounds. They also performed a nested cross-validation of their model, which resulted in a MAE of 18.3 K. We did not include the tgBoost model in our comparison plots, because it was built on nearly the same data set as used in our models introduced here, and it is not clear to us which of these experimental data were used for training and which for testing. Therefore, we devised an alternative procedure to provide a direct and fair comparison of the two ML models. In order to do this, we used an entirely independent data set of Alzghoul et al.^11^ for testing their performance. Although this data set consists of a relatively specific data set of organic druglike substances, it fulfills valuable criteria for the comparison, since it is guaranteed that none of the two models has seen the data before. Not every data point out of the 71 substances provided could be used in the comparison, because the tgBoost model is not meant for handling molecules with a molar mass larger than 500 g mol^–1^. Moreover, also all sulfur-containing compounds were excluded, since neither of the two ML models was actually trained on such compounds. The remaining data set contained 41 compounds with experimental *T*_g_ values, which is still a decent size for a test set.

**Figure 7:**
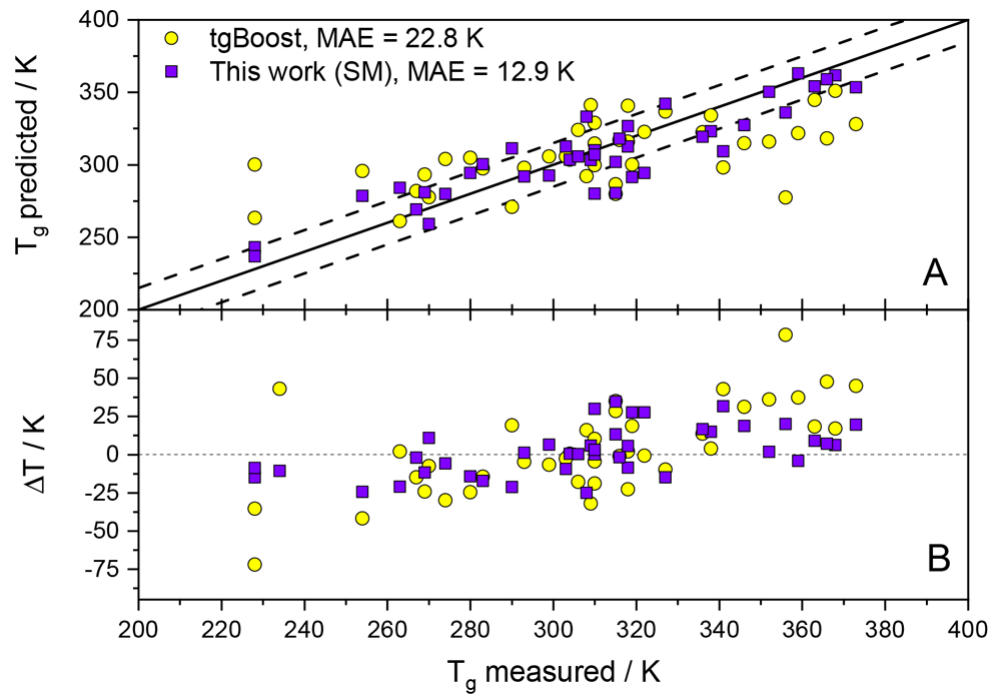
Comparison of the tgBoost ML model^12^ (yellow circles) and the SMILES Mode ML model from this work (purple squares) on a literature test set with relatively complex molecular structures. Panel A shows the predicted *T*_g_ values versus the experimental *T*_g_ values (dashed lines mark ±15 K), while in panel B the deviations  for each *T*_g_ value are presented. In Figure S5, we also show the prediction results of the other parametrizations for this data set.

Figure 7 shows the predicted *T*_g_ versus the measured *T*_g_ for the two ML models. The graph reveals that for the majority of the samples the SMILES Mode ML model developed here provides more accurate predictions than the tgBoost model, which is also evident from the fact that the MAE values are 12.9 K for our model and 22.8 K for the tgBoost model. In addition, Figure 7B visualizes that the spread of the predicted values is more narrow for our SM model. One striking observation is that for samples with higher *T*_g_ values, both models underestimate the experimental *T*_g_ values, whereas for *T*_g_ values below about 310 K a slight trend to overestimation is observed. One possible explanation for this behavior may be that the druglike molecules in this data set are predominantly annulated poly heterocycles with additional functional substituents. A problem with such molecules may be that the models tend to overestimate the accumulated effect of these functional groups and, hence, predict a glass transition temperature that is too high. We surmise that this behavior may be overcome by including more of such highly functionalized molecules in the training data, thereby representing such species better. Such extension of the current models is left to future studies.

## 4 Model Deployment

### 4.1 Web Site

The application of machine learning to many types of problems has become a frequently used tool in the recent past.^51,52^ In addition to domain-specific expertise in the area of the particular problem, the usage of ML requires a sophisticated technical understanding in statistics and informatics. Unfortunately, even when ML models are published, they can not be easily executed by foreign users without technical obstacles. Therefore, we have developed a user-friendly web application for our ML models, which can be accessed via any standard web browser at https://tgml.chemie.uni-bielefeld.de. In this way, we want to make our model and its results available to the public. For this purpose, we chose an architecture that supports the user by automatically selecting the best model, i.e., that with the lowest MAE, for a particular set of input variables, making it comfortable and easy to use.

On accessing the web site, the user is presented with an overview page containing information about the ML model and instructions on how to operate it. Based on the molecular information available, either the Functional Group Mode or the SMILES Mode can be selected, and we recommend the SMILES Mode because of its lower MAE as mentioned before. When the SMILES Mode is selected, the user is presented with a simple input form with two fields, one mandatory field for the SMILES string of the molecule of interest and another optional field for its melting temperature. If both are available, then we recommend entering both for best accuracy. After clicking the “Start Calculation” button, an implemented input filter checks the user-given input for syntax errors in the SMILES string or unrealistic melting temperatures, and then the application automatically selects the respective ML model and determines the predicted glass transition temperature *T*_g_. Thereafter, a result window opens, which displays all relevant parameters (type of model and mode, the SMILES string, chemical formula, melting temperature, and molar mass of the compound, and finally the model’s *T*_g_ prediction and the associated mean absolute error). All output parameters can be copied into the clipboard by a simple click for further processing. When the Functional Group Mode is selected, the user is presented with an input form, where the number of different functional groups, the double bond equivalent and optionally the melting temperature can be entered. Again, an input filter is used for a quick and direct plausibility check of the input parameters. After starting the calculation, a result page with all relevant input and output data is presented to the user. Further technical details on the architecture of the web site are given in the Supporting Information.

The provision of our ML model for the prediction of glass transition temperatures as a web application has several advantages. First and foremost, technical barriers are greatly reduced for any user trying to make a *T*_g_ prediction for a particular compound. In addition, future updated versions of the model can be easily implemented on the web site, ensuring that users always use the latest up-to-date version (to ensure the reproducibility of past results, we are planning to offer older versions of the model on a separate subpage in the future, once applicable). We further note that we intend to improve the model over time by retraining the algorithms once sufficient new experimental data points are available. Therefore, we encourage everyone to submit their experimental glass transition temperature data via a contact form on the web site on the subpage “Submit Data”.

### 4.2 Python code

Furthermore, we also provide files for the execution of the model via a Python development environment. The bundle consists of the main Python script, a readme.txt, and a requirements_console_script.txt specifying the required packages, and the trained model modes as pickle files (pickle is a package for saving and loading trained algorithms). In the Python script, explanations for the correct input format are given along with examples. The code also enables multicomponent input, which may be a fast and convenient option for some applications. Generally, this code can be used for the further implementation into other programs or models and, therefore, could be interesting for the modeling community. The bundle is available at DOI: 10.5281/zenodo.7650576.

## 5 Summary and Conclusions

In this work, we presented a new ML model for the prediction of the glass transition temperature of organic molecular compounds. Two alternative modes were developed, in which the molecular information is either encoded in the type and number of functional groups or is extracted from a SMILES string. Both modes show increased accuracy with the additional input of the melting temperature *T*_m_ as an optional feature. The model was trained with up to 330 different organic compounds, including those containing nitrogen and halogen atoms. The ML model operates on an *extra trees* regressor, which is an ensemble method based on decision trees. An evaluation of the performance showed that the model’s predictions were significantly more accurate than those of conventional methods, i.e., fitting-based parametrizations, which was confirmed by direct comparisons with individual test sets as well as by a nested cross-validation procedure resulting in a MAE of 12–13 K (see Tables 3 and 4). For these reasons, we recommend using our new ML-based prediction model as an alternative to previously introduced conventional prediction methods to calculate *T*_g_. These parameterizations^3,4^ have been used to predict *T*_g_ as an input parameter for estimating aerosol viscosity in other types of models.^53−56^ We suppose that using our ML model for predicting *T*_g_ in those models could lead to improved results.

As the ML model cannot be provided as an analytic equation, we developed a public online version of the model, where the users can input the relevant variables of the desired molecule on a web site and receive the predicted *T*_g_ value as an output, without having to run the actual full code in Python environment on their own hardware. For in-model applications we also provide a Python code for use of the different models.

Our sensitivity tests revealed that the ML model has the ability to not only accurately predict the *T*_g_ of simple molecules, but also shows high accuracy in the prediction of *T*_g_ of highly complex structures. Altogether, the results suggest that the ML model is expected to maintain a robust performance throughout many different applications in various fields.

## Data Availability

The experimental data set used in this study is publicly available via the Zenodo repository at DOI: 10.5281/zenodo.7319485. A Python script for the execution of the model and *T*_g_ prediction is available at DOI: 10.5281/zenodo.7650576.

## Supporting Information Available

Information on data preprocessing, model analysis (feature correlation and relative feature importance), technical details of the web site, tables with metric scores of the model intercomparisons, a model info sheet, and database of all experimental *T*_g_ values used in this study (PDF)
