# Supplementary material for: Machine-Learning-Based Prediction of the Glass Transition Temperature of Organic Compounds Using Experimental Data
Source: ACS Omega. 2023 Mar 22;8(13):12298–309. doi: 10.1021/acsomega.2c08146 (PMC10077449; doi:10.1021/acsomega.2c08146)
Supplement: Supplementary file 1 — ao2c08146_si_001.pdf [file ao2c08146_si_001.pdf]

Supplementary Information to:

Machine Learning-Based Prediction of the Glass Transition Temperature of Organic Compounds Using Experimental Data

Gianluca Armeli, Jan-Hendrik Peters, and Thomas Koop*

Faculty of Chemistry, Bielefeld University, 33615 Bielefeld, Germany

E-mail: thomas.koop@uni-bielefeld.de

Data pre-processing

In order to avoid the phenomenon of 'data leakage'[1] due to duplicate samples, the raw data were pre-processed prior to further applications. For this purpose, a code was written that loops through the data set and identifies duplicates for each feature vector (X) and adds them to a list. Then an array with the median T_g values (Y) for every X was created. Finally, the numpy.unique method was applied to remove all the duplicates and generate the unified data set.

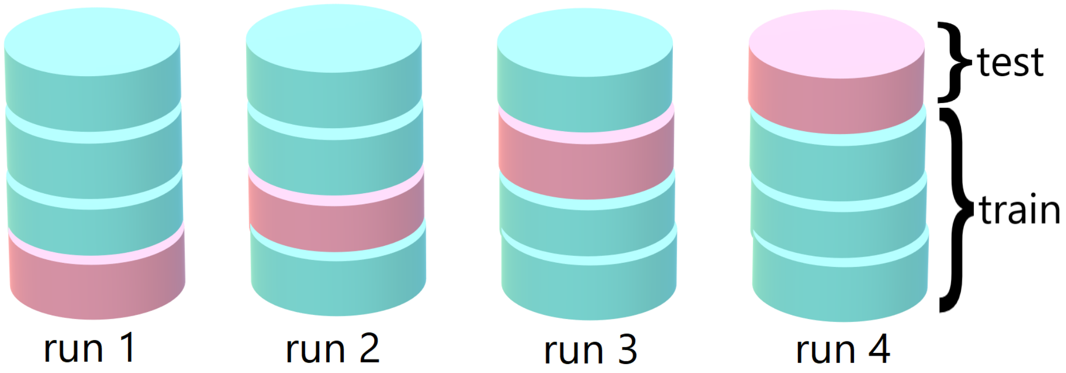

Figure S1: Exemplary illustration of a k-fold cross-validation for $k = 4$. The data set is split into four equally large segments. A total of four runs are performed, each time using a different segment as the test set. In our study, we performed a 10-fold cross-validation, i.e. $k = 10$.

Further model evaluation

In general, multicollinearity caused by redundant features is a problem in ML.[2,3] Since the features used in the Functional Group Mode in part depend upon each other, we discuss this aspect here in more detail. First of all, it is generally desirable to keep the number of features small, because the more there are, the longer the model will require for training. There are various dimensionality reduction techniques available for reducing the number of features and identifying redundant ones. We already have a relatively small set of features, so that the aspect of computational time is negligible. However, some algorithms such as linear regression are affected negatively by highly correlated features, because they hamper or even prevent the correct determination of the fitting coefficients. In our ML model, we use a decision tree-based approach, in which each feature is evaluated individually, and so ultimately the best option is selected by the model. Therefore, we consider the model to be rather robust against such cross-correlations.

Nevertheless, we calculated a correlation matrix given in Figure S2 that shows the Pearson correlation coefficient of the feature-feature correlations in the triangle on the left hand side. Features are considered as highly correlated when the correlation coefficient is greater than 0.9.[2,3] This is not the case for any of our features. The only case, where this value is exceeded is the correlation between the T_{m} feature and the T_{g} label. However, this is not a multicollinearity problem, but a desired feature-label correlation. In fact, this particular correlation is indicative of the well known Boyer-Beaman relationship.[4–6] So it is not surprising, in fact re-assuring that the model reveals such a high correlation value for this feature-label correlation.

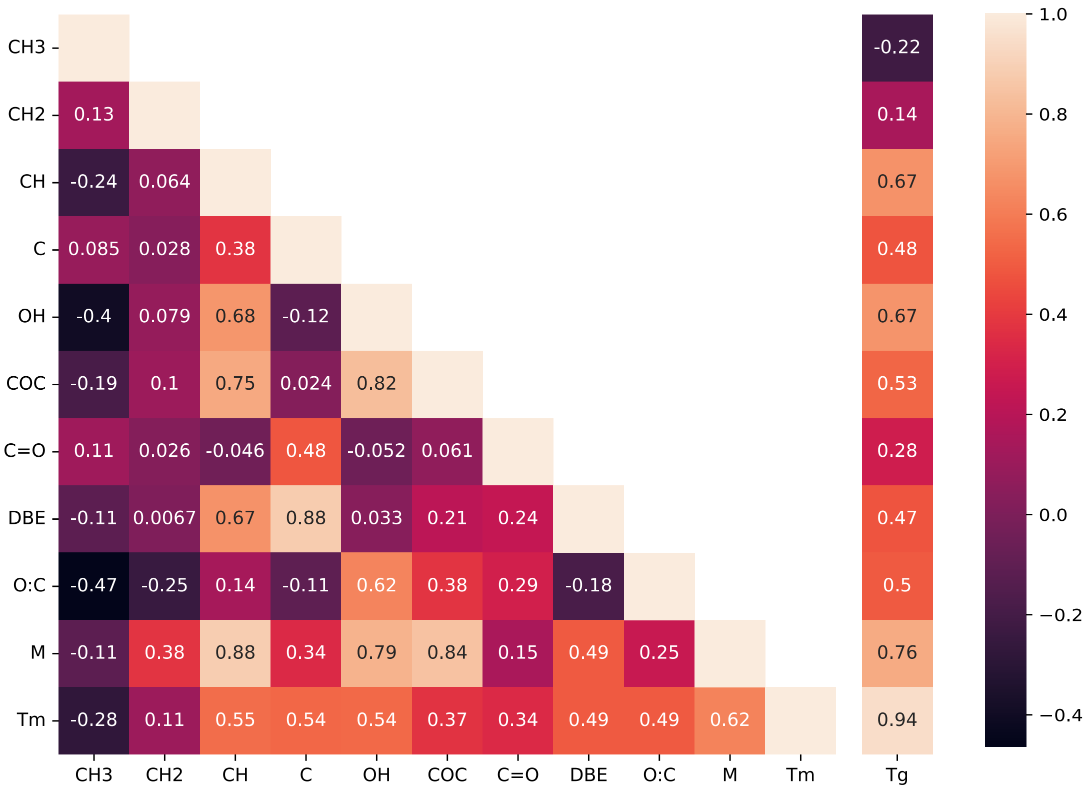

Figure S2: Correlation matrix displaying the Pearson correlation coefficients of the feature-feature correlations (triangle on the left) and the feature-label correlations (single-column at the right). For details, see text.

Other features that could be associated with a high feature-feature correlation are the molar mass M and the atomic oxygen-to-carbon ratio O:C. To explicitly test this issue, we also ran an *extra trees* model that was trained without the T_m, M and O:C feature, resulting in a slight deterioration of the MAE from 13.1 K to 14.1 K, similar to the one encountered when dropping the T_m feature only. We also analyzed the resulting feature importances. Those values are then compared in Figure S3 to the original model that was trained with all the features, but had the feature importance for T_m, M and O:C removed and subsequently renormalized the remaining relative importance feature values. Figure S3 shows that overall the feature importance values of both models have not changed much, indicating that the original model is not negatively influenced by the additional features. The comparison indicates that the number of OH-groups is by far the most important feature, in line with the

fact that OH-groups contribute to the formation of intermolecular hydrogen bonds, which are an essential property contributing to the viscosity and, hence, also the glass transition temperature of organic compounds, as noted previously.[7,8] Moreover, a slight increase in the importance of the feature 'number of carbon atoms not bonded to hydrogen atoms' (C) is observed in the reduced model. The reason for this observation is probably that this feature, together with the CH feature (number of methine groups), is more frequent in larger molecules and, therefore, becomes more pronounced when the molar mass M is discarded as a feature.

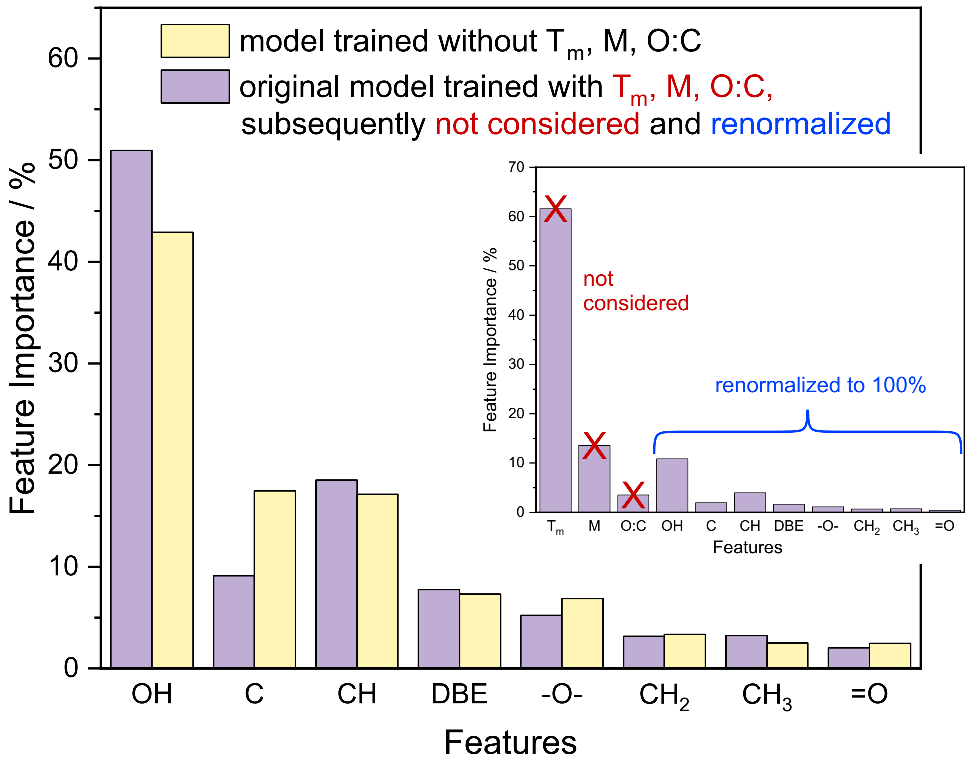

Figure S3: Relative feature importance of the Functional Group Mode on the CHO data set without considering the T_{m}, M and O:C features (yellow bars). For comparison, the lilac bars were generated by calculating the feature importances from the FG model including the T_{m}, M and O:C features (see inset, which is identical to the lilac bars in Figure 4 of the main paper), and subsequently removing those features after training and renormalizing the remaining feature values to 100%.

Figure S4 shows the T_g prediction of the SMILES Mode for different molecular species as a function of the total number of carbon atoms in the molecule. In analogy to the work by Rothfuss and Petters[7] on the viscosity of organic compounds, the considered species were: n-alkanes (gray squares), n-alcohols (blue circles), diols (green triangles) and triols (red inverted triangles), see Table S1 for the SMILES code strings of the diols and triols. Open symbols represent values calculated with the no T_m mode, whereas filled symbols where generated using T_m as an input feature. The length of the alkyl chain ranges from three to ten carbon atoms. The graph shows that the addition of an OH-group or a C-atom usually leads to a higher T_g value. T_g generally increases slightly with the number of carbon atoms within a compound class, and T_g strongly increases with the number of OH-groups at constant number of carbon atoms. Thus, the model is consistent with prior studies[7,9] and produces a physically meaningful behavior.

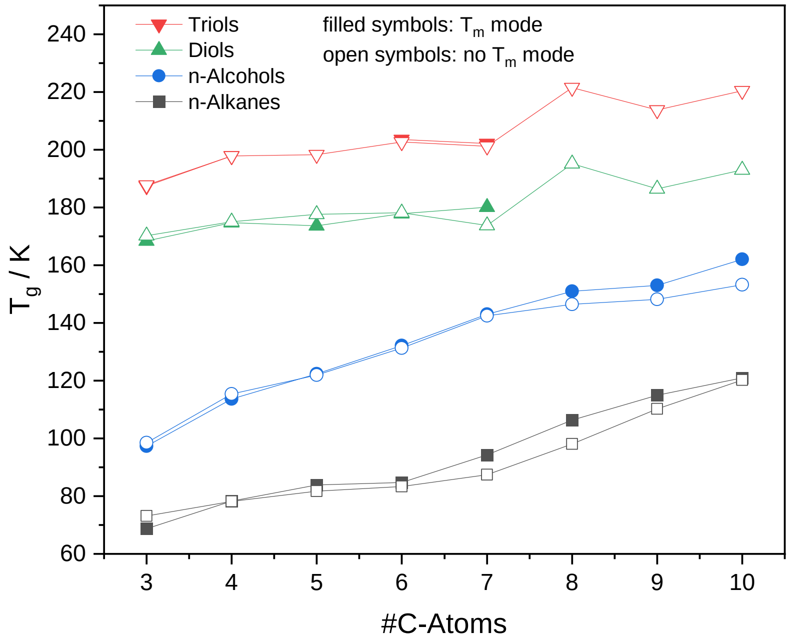

Figure S4: Predicted T_g values for a series of molecular compounds using the SMILES Mode. The x-axis represents the total number of carbon atoms in the linear molecules. Lines between symbols are just to guide the eye and have no physical meaning. T_g values for the larger diols and triols could not be calculated in the T_m mode as no experimental T_m values are available for these compounds.

Table S1: SMILES code of series of molecular compounds shown in Figure S4

#C	Diols	Triols
3	CC(O)CO	OCC(O)CO
4	CC(CCO)O	C(CO)C(CO)O
5	CC(CCCO)O	C(CO)C(CCO)O
6	CC(CCCCO)O	C(CCO)CC(CO)O
7	OCCCCCCCO	C(CCC(CO)O)CCO
8	CCCCCCC(CO)O	CCCCCC(C(CO)O)O
9	OCCCCCCCCCO	C(CCCC(CO)O)CCCO
10	OCCCCCCCCCCO	C(CCCCO)CCCC(CO)O

Figure S5 shows the model predictions on the drug data set as already given in Figure 7A of the main article, but now also including the predictions of the parametrizations. The parametrization of Shiraiwa et al.[10] (orange circles) combined with the one of Li et al.[11] (green inverted triangles) achieved an MAE of 35.3 K, the parametrization of DeRieux et al.[12] (blue triangles) combined with Li et al.[11] produced an MAE of 35.9 K and the Boyer-Beaman rule (cyan diamonds) resulted in an MAE of 16.4 K. For comparison, our SMILES Mode ML model resulted in an MAE of 12.9 K.

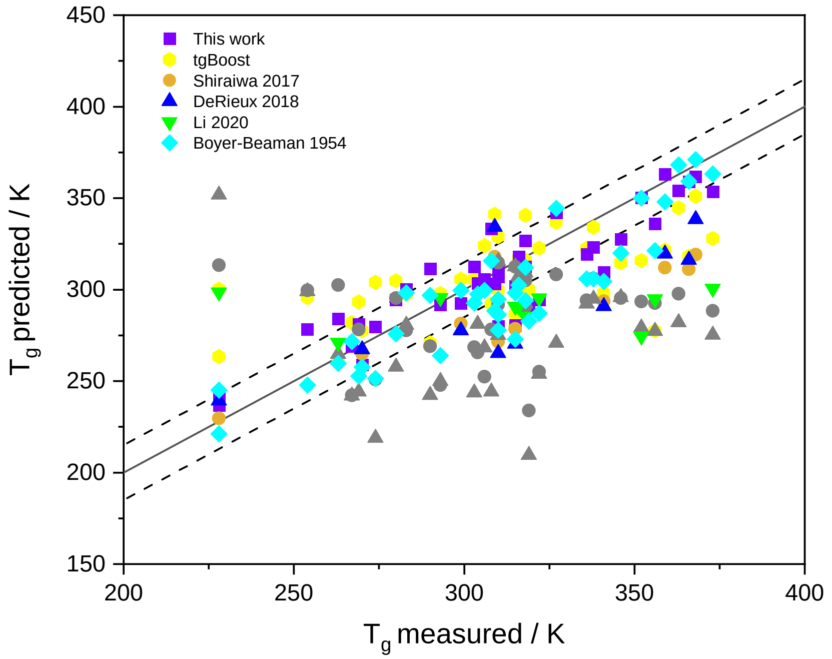

Figure S5: Predicted T_g versus measured T_g for the drug data set, also including the predictions by the various parametrizations. Gray masked symbols originate from nitrogen or halogen compounds and were calculated with the Shiraiwa or DeRieux formulas, which shall be applied to CHO compounds only. The dashed lines indicate a deviation of ±15 K from the 1:1 line (solid line).

Tables with metric scores

Table S2: Metric scores (MAE: Mean Absolute Error, RMSE: Root Mean Squared Error, R^2: Coefficient of Determination, MAPE: Mean Absolute Percentage Error) on the CHO test set using the Functional Group Mode.

	MAE / K	RMSE / K	R^2	MAPE / %
Shiraiwa 2017	25.5	34.6	0.81	15.5
DeRieux 2018	24.0	32.9	0.83	15.4
Boyer-Beaman 1954	10.1	15.0	0.97	5.6
This work (FGM)	6.9	10.6	0.98	3.9

Table S3: Metric scores (MAE: Mean Absolute Error, RMSE: Root Mean Squared Error, R^2: Coefficient of Determination, MAPE: Mean Absolute Percentage Error) on the NHal test set using the Functional Group Mode.

	MAE / K	RMSE / K	R^2	MAPE / %
Shiraiwa 2017	32.8[a]	41.2[a]	0.65[a]	15.0[a]
DeRieux 2018	27.0[a]	34.3[a]	0.76[a]	11.2[a]
Boyer-Beaman 1954	17.2	20.8	0.93	9.2
This work (FGM)	9.9	12.4	0.97	4.7

[a] for CHON compounds the parametrization from Li et al.[11] was used; other compounds (e.g. CHN, CHHal etc.) were not considered

Table S4: Metric scores (MAE: Mean Absolute Error, RMSE: Root Mean Squared Error, R^2: Coefficient of Determination, MAPE: Mean Absolute Percentage Error) on the NHal test set using the SMILES Mode.

	MAE / K	RMSE / K	R^2	MAPE / %
Shiraiwa 2017	28.9[a]	41.0[a]	0.74[a]	13.4[a]
DeRieux 2018	28.8[a]	42.0[a]	0.73[a]	13.3[a]
Boyer-Beaman 1954	19.8	24.6	0.92	10.5
This work (SM)	10.6	14.7	0.97	5.2

[a] for CHON compounds the parametrization from Li et al.[11] was used; other compounds (e.g. CHN, CHHal etc.) were not considered

Table S5: Metric scores (MAE: Mean Absolute Error, RMSE: Root Mean Squared Error, R^2: Coefficient of Determination, MAPE: Mean Absolute Percentage Error) on the drug data set using the SMILES Mode.

	MAE / K	RMSE / K	R^2	MAPE / %
Galeazzo 2022	22.8	29.2	0.39	7.6
This work (SM)	12.7	16.0	0.82	4.2

Technical Details of the Website

A flow chart of the different software components used for the web-based version of the ML model is shown in Fig S6. The basis forms an Ubuntu 22.04 Long-Term-Support (LTS) Linux server. When users connect to the website via a web browser, they send a Hypertext Transfer Protocol (HTTP) request to the server, which is processed by a Nginx reverse proxy web server.[13] Nginx forwards this request to the Gunicorn web server, which is able to deliver Python web applications via the Web Server Gateway Interface (WSGI).[14] The combination of Nginx and Gunicorn optimizes security, performance and functionality of the system architecture. The web application for the usage of the ML model is written in Python and is based on the Flask web framework.[15] In addition, the Supervisor software monitors all processes related to the Gunicorn web server and restarts them automatically if necessary.[16] Flask is a minimalist, easy to use web framework that allows for a simple combination of HTML templates with Python code. The Python packages and modules used for our application are given in Table S6. To ensure a modern and responsive website design, we used the Bootstrap framework for HTML templates.[17] Further technical details are available from the authors on reasonable request.

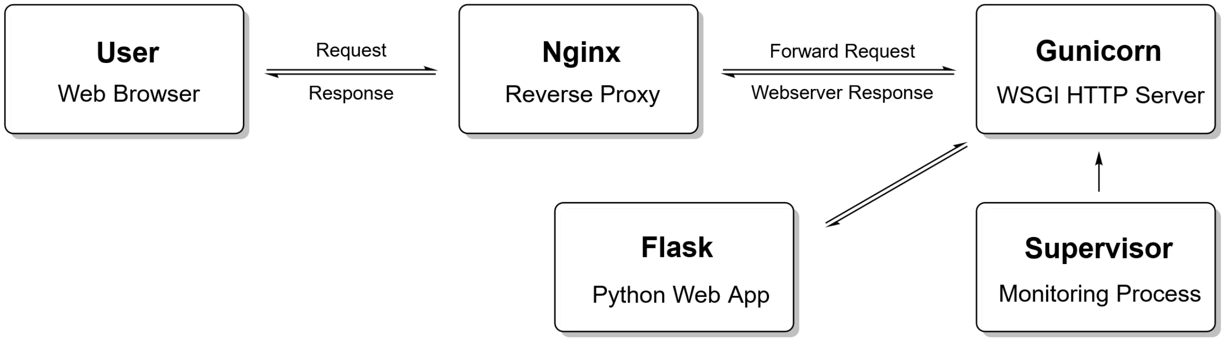

Figure S6: Flow chart of the software components used for deployment of the Python application.

Table S6: Versions of the Python packages or modules used for the Flask web application.

Python Package or Module	Version
Click[18]	8.1.3
DeepChem[19]	2.5.0
Flask[20]	2.1.2
Gunicorn[21]	20.1.0
Numpy[22]	1.22.4
Pandas[23]	1.4.2
RdKit-pypi[24]	2022.3.2.1
SciKit-Learn[25]	1.1.1
Tensorflow-cpu[26]	2.9.0
Werkzeug[27]	2.1.2

Model info sheet

To improve the reproducibility and transparency of ML studies, Kapoor and Narayanan[1] created a model info sheet with pre-defined questions that authors of new studies should answer. In the following, the model info sheet for the current study is completed.

Information about paper

1) Author(s): Names of the authors of the paper or report

Gianluca Armeli, Jan-Hendrik Peters, Thomas Koop

2) Title of the paper or report which introduces the model

Machine Learning-Based Prediction of the Glass Transition Temperature of Organic Compounds Using Experimental Data

3) DOI or permanent link to the paper or report (for example, link to arxiv.org webpage)

None

4) License: Under which license(s) are the data and/or model shared?

The data set is shared under a CC-BY 4.0 International license.

5) Email address of the corresponding author

thomas.koop@uni-bielefeld.de

Scientific claim(s) of interest

6) Does your paper make a generalizable claim based on the ML model? If yes, what is the scientific claim? For example, "Our ML model can be used to diagnose Covid-19 using chest radiographs of adult patients".

Our ML model can be used to predict the glass transition temperature of organic molecular compounds.

7) Is the scientific claim made about a distribution or population from which you can sample? If yes: (a) what is the population or distribution about which the scientific claim is being made? (b) What is the sample used for the study? For example, "(a) Population: adult patients with symptoms of Covid-19. (b) Sample: We use a random sample of adult patients who present at a U.S. based hospital between April 2020 and June 2020".

(a) Distribution: Organic molecular compound containing C-, H-, O-, N-, F-, Cl-, Br- and I-atoms. (b) Sample: The data was extracted from scientific publications and from experimental results obtained in our laboratory.

8) Does the scientific claim only apply to certain subsets of the distribution mentioned in Q6? For example, "Our model works on chest radiographs of U.S.-based adult patients and might not generalize to radiographs taken in other places or using different machines."

Our model works for the compounds described in Q7b and may not be generalized to other types of molecules.

Train-test split is maintained across all steps in creating the model

9) Train-test split type: How was the dataset split into train and test sets? (For example, cross-validation; separate train and test sets).

For the overall model evaluation, a nested cross-validation was performed. In addition, train/test splits were generated by a pseudo random number generator.

10) Are there duplicates in the dataset? If yes, explain how duplicates are handled to ensure the train-test split.

Duplicates were handled as described in section 'Data pre-processing' above.

11) In case the dataset has dependencies (e.g., multiple rows of data from the same patient), describe how the dependencies were addressed (for example, using block-cross validation).

Our data set does not have such dependencies.

12) List all the pre-processing steps used in creating your model. For example, imputing missing data, normalizing feature values, selecting a subset of rows from the dataset for building the model.

Pre-processing of the data was performed as described in section 'Data pre-processing' above.

13) How was the train-test split observed during each pre-processing step? If applicable, use a separate line for each step mentioned in Q12.

During pre-processing train/test splits were not necessary and therefore not performed.

14) List all the modeling steps used in creating your model. For example, feature selection, parameter tuning, model selection.

After pre-processing, an algorithm (*extra trees* regression) was selected through cross-validation. Some features were selected by recursive feature elimination. The parameters were tuned by grid search cross-validation.

15) How was the train-test split observed during each modeling step? If applicable, use a separate line for each step mentioned in Q14.

Each modeling step of Q14 was applied to the training set only, in order to avoid any bias from the test set.

16) List all the evaluation steps used in evaluating model performance. For example, cross-validation, out-of-sample testing.

For evaluation, a nested cross-validation was applied to the whole data set. The procedure itself maintains legitimate train/test splits during each step.

17) How was the train-test split observed during each evaluation step? If applicable, use a separate line for each step mentioned in Q16.

See Q16.

Test set is drawn from the distribution of scientific interest

18) Why is your test set representative of the population or distribution about which you are making your scientific claims?

Our data set contains a variety of different chemical compounds that are also equally represented (by random sampling) in the test set.

19) Explain the process for selecting the test set and why this does not introduce selection bias in the learning process.

We collected all the data currently available for experimental T_g values of organic molecular compounds and added them to our data set. Then, the test set was randomly picked by a pseudo random number generator and, hence, we do not expect any selection bias.

20) In case your model is used to predict a future outcome of interest using past data, detail how data in the training set is always from a date earlier than the data in the test set.

Our label is a property that is independent of time.

Each feature used in the model is legitimate for the task

21) List the features used in the model, alongside an argument for their legitimacy. A legitimate feature is one that would be available when the model is used in the real world and is not a proxy of the outcome being predicted. You can also include this list in an appendix and reference the relevant section of your Appendix here.

We chose representations of the chemical structure of organic compounds, since our label T_g has shown to follow several structure-property relationships.[10,12] Therefore, our features are numeric representations of structural elements of molecules, for example the number of functional groups, molar mass etc.

References

(1) Kapoor, S.; Narayanan, A. Leakage and the Reproducibility Crisis in ML-based Science. *arXiv preprint arXiv:2207.07048* **2022**,

(2) Alin, A. Multicollinearity. *Wiley interdisciplinary reviews: computational statistics* **2010**, *2*, 370–374.

(3) Daoud, J. I. Multicollinearity and regression analysis. Journal of Physics: Conference Series. 2017; p 012009.

(4) Beaman, R. G. Relation between (apparent) second-order transition temperature and melting point. *Journal of Polymer Science* **1952**, *9*, 470–472.

(5) Boyer, R. F. Relationship of first-to second-order transition temperatures for crystalline high polymers. *Journal of Applied Physics* **1954**, *25*, 825–829.

(6) Okui, N. Relationships between melting temperature, maximum crystallization temperature and glass transition temperature. *Polymer* **1990**, *31*, 92–94.

(7) Rothfuss, N. E.; Petters, M. D. Influence of functional groups on the viscosity of organic aerosol. *Environmental Science & Technology* **2017**, *51*, 271–279.

(8) Koop, T.; Bookhold, J.; Shiraiwa, M.; Pöschl, U. Glass transition and phase state of organic compounds: dependency on molecular properties and implications for secondary organic aerosols in the atmosphere. *Physical Chemistry Chemical Physics* **2011**, *13*, 19238–19255.

(9) Galeazzo, T.; Shiraiwa, M. Predicting glass transition temperature and melting point of organic compounds via machine learning and molecular embeddings. *Environmental Science: Atmospheres* **2022**, *2*, 362–374.

(10) Shiraiwa, M.; Li, Y.; Tsimpidi, A. P.; Karydis, V. A.; Berkemeier, T.; Pandis, S. N.; Lelieveld, J.; Koop, T.; Pöschl, U. Global distribution of particle phase state in atmospheric secondary organic aerosols. *Nature communications* **2017**, *8*, 15002.

(11) Li, Y.; Day, D. A.; Stark, H.; Jimenez, J. L.; Shiraiwa, M. Predictions of the glass transition temperature and viscosity of organic aerosols from volatility distributions. *Atmospheric Chemistry and Physics* **2020**, *20*, 8103–8122.

(12) DeRieux, W.-S. W.; Li, Y.; Lin, P.; Laskin, J.; Laskin, A.; Bertram, A. K.; Nizkorodov, S. A.; Shiraiwa, M. Predicting the glass transition temperature and viscosity of secondary organic material using molecular composition. *Atmospheric Chemistry and Physics* **2018**, *18*, 6331–6351.

(13) Sysoev, I. Nginx, Inc. *https://nginx.org* **2022**,

(14) Chesneau, B. Gunicorn. *https://gunicorn.org* **2022**,

(15) Ronacher, A. *https://palletsprojects.com/p/flask* **2022**,

(16) Consulting, A.; Contributors Supervisor: A Process Control System. *https://supervisord.org* **2022**,

(17) Bootstrap Version 5.2.0. *https://getbootstrap.com* **2022**,

(18) Click Version 8.1.3. *https://pypi.org/project/click/8.1.3* **2022**,

(19) Deepchem Version 2.5.0. *https://pypi.org/project/deepchem/2.5.0* **2022**,

(20) Flask Version 2.1.2. *https://pypi.org/project/Flask/2.1.2* **2022**,

(21) Gunicorn Version 20.1.0. *https://pypi.org/project/gunicorn/20.1.0* **2022**,

(22) Numpy Version 1.22.4. *https://pypi.org/project/numpy/1.22.4* **2022**,

(23) Pandas Version 1.4.2. *https://pypi.org/project/pandas/1.4.2* **2022**,

(24) RdKit-Pypi Version 2022.3.2.1. *https://pypi.org/project/rdkit-pypi/2022.3.2.1* **2022**,

(25) SciKit-Learn Version 1.1.1. *https://pypi.org/project/scikit-learn/1.1.1* **2022**,

(26) Tensorflow-cpu Version 2.9.0. *https://pypi.org/project/tensorflow-cpu/2.9.0* **2022**,

(27) Werkzeug Version 2.1.2. *https://pypi.org/project/werkzeug/2.1.2* **2022**,

Name	Formula	#CH3	#CH2	#CH	#C	#OH	#C-O-C	#O=C	DBA	#N	#Hal	O:C	M / g/mol	Tm / K	Tg / K	Tg/Tm	Reference(Tm)	Reference(Tg)	SMILES	Figure
glucose	C6H12O6	0	1	4	1	5	0	1	1	0	0	1.0000	180	419	303	0.7232	kercthermochimacta95	SimonBA	C(C1C(C(C(C(O1)O)O)O)O)O	6
glucose	C6H12O6	0	1	4	1	5	0	1	1	0	0	1.0000	180	414	290	0.7005	beamanjpolysci52	beamanjpolysci52	C(C1C(C(C(C(O1)O)O)O)O)O	6
glucose	C6H12O6	0	1	4	1	5	0	1	1	0	0	1.0000	180	432.9	309	0.7138	murthyjchemsocfaradaytrans93	murthyjchemsocfaradaytrans93	C(C1C(C(C(C(O1)O)O)O)O)O	6
glucose	C6H12O6	0	1	4	1	5	0	1	1	0	0	1.0000	180	419.15	309	0.7372	wangjchemphys06	wangjchemphys06	C(C1C(C(C(C(O1)O)O)O)O)O	6
glucose	C6H12O6	0	1	4	1	5	0	1	1	0	0	1.0000	180	414	290	0.7005	naitojphychem93	naitojphychem93	C(C1C(C(C(C(O1)O)O)O)O)O	6
glucose	C6H12O6	0	1	4	1	5	0	1	1	0	0	1.0000	180	419	303	0.7232	kercthermochimacta95	kercthermochimacta95	C(C1C(C(C(C(O1)O)O)O)O)O	6
dextrose	C6H12O6	0	1	4	1	5	0	1	1	0	0	1.0000	180	432	310	0.7176	kercthermochimacta95	kercthermochimacta95	C(C1C(C(C(C(O1)O)O)O)O)O	6
tartaric acid	C4H6O6	0	0	2	2	4	0	2	2	0	0	1.5000	150	430	291	0.6767	kercthermochimacta95	SimonBA	O=C(O)C(O)C(O)C(=O)O	
1,2,3-propanetricarboxylic acid	C6H8O6	0	2	1	3	3	0	3	3	0	0	1.0000	176	431.15	284	0.6587	koop2011	SimonBA	C(C(CC(=O)O)C(=O)O)C(=O)O	
1,2,4-butanetricarboxylic acid	C7H10O6	0	3	1	3	3	0	3	3	0	0	0.8571	190	391.65	276	0.7047	koop2011	SimonBA	C(CC(=O)O)C(CC(=O)O)C(=O)O	5A
glycerol	C3H8O3	0	2	1	0	3	0	0	0	0	0	1.0000	92	291	180	0.6186	kercthermochimacta95	kercthermochimacta95	OCC(O)CO	5B
glycerol	C3H8O3	0	2	1	0	3	0	0	0	0	0	1.0000	92	291.25	190	0.6524	wangjchemphys06	wangjchemphys06	OCC(O)CO	5B
glycerol	C3H8O3	0	2	1	0	3	0	0	0	0	0	1.0000	92	291	185	0.6357	beamanjpolysci52	beamanjpolysci52	OCC(O)CO	5B
glycerol	C3H8O3	0	2	1	0	3	0	0	0	0	0	1.0000	92	291.25	189.9	0.6520	wangjchemphys08	wangjchemphys08	OCC(O)CO	5B
glycerol	C3H8O3	0	2	1	0	3	0	0	0	0	0	1.0000	92	292	186	0.6370	naitojphychem93	naitojphychem93	OCC(O)CO	5B
glycerol	C3H8O3	0	2	1	0	3	0	0	0	0	0	1.0000	92	291.25	186	0.6386	carpenterjchemphys67	carpenterjchemphys67	OCC(O)CO	5B
ribose	C5H10O5	0	1	3	1	5	0	1	1	0	0	1.2000	166	360	263	0.7306	kercthermochimacta95	kercthermochimacta95	C1C(C(C(C(O1)O)O)O)O	
ethanol	C2H6O	1	1	0	0	1	0	0	0	0	0	0.5000	46	161	96	0.5963	naitojphychem93	naitojphychem93	OCC	
n-propanol	C3H8O	1	2	0	0	1	0	0	0	0	0	0.3333	60	148.76	98	0.6588	carpenterjchemphys67	carpenterjchemphys67	CCCO	
n-propanol	C3H8O	1	2	0	0	1	0	0	0	0	0	0.3333	60	148.76	96.2	0.6467	wangjchemphys06	wangjchemphys06	CCCO	
n-propanol	C3H8O	1	2	0	0	1	0	0	0	0	0	0.3333	60	148.76	103	0.6924	murthyjchemsocfaradaytrans93	murthyjchemsocfaradaytrans93	CCCO	
n-propanol	C3H8O	1	2	0	0	1	0	0	0	0	0	0.3333	60	146	93	0.6370	naitojphychem93	naitojphychem93	CCCO	
n-propanol	C3H8O	1	2	0	0	1	0	0	0	0	0	0.3333	60	146	93	0.6370	beamanjpolysci52	beamanjpolysci52	CCCO	
n-propanol	C3H8O	1	2	0	0	1	0	0	0	0	0	0.3333	60	148.76	99.95	0.6719	lesikarjchemphys75	lesikarjchemphys75	CCCO	
n-propanol	C3H8O	1	2	0	0	1	0	0	0	0	0	0.3333	60	148.76	99.85	0.6712	lesikar_jsolchem_77	lesikar_jsolchem_77	CCCO	
methanol	CH4O	1	0	0	0	1	0	0	0	0	0	1.0000	32	175.62	100	0.5694	wangjchemphys06	wangjchemphys06	CO	5A
methanol	CH4O	1	0	0	0	1	0	0	0	0	0	1.0000	32	175	103	0.5886	naitojphychem93	naitojphychem93	CO	5A
methanol	CH4O	1	0	0	0	1	0	0	0	0	0	1.0000	32	175.62	102.65	0.5845	lesikarjchemphys75	lesikarjchemphys75	CO	5A
methanol	CH4O	1	0	0	0	1	0	0	0	0	0	1.0000	32	175.62	102.75	0.5851	lesikar_jsolchem_77	lesikar_jsolchem_77	CO	5A
ethylenglycol	C2H6O2	0	2	0	0	2	0	0	0	0	0	1.0000	62	260.46	151	0.5797	wangjchemphys06	wangjchemphys06	OCCO	5B
ethylenglycol	C2H6O2	0	2	0	0	2	0	0	0	0	0	1.0000	62	260	152	0.5846	naitojphychem93	naitojphychem93	OCCO	5B
phenolphthalein	C20H14O4	0	0	12	8	2	1	1	14	0	0	0.2000	318	535.65	363	0.6777	wangjchemphys06	wangjchemphys06	O=C1OC(C2=C1C=CC=C2)(C3=CC=C(C=C3)O[H])C4=CC=C(C=C4)O[H]	
maltitol	C12H24O11	0	3	9	0	9	2	0	1	0	0	0.9167	344	418.15	311	0.7438	wangjchemphys06	wangjchemphys06	C(C1C(C(C(C(O1)OC(C(CO)O)C(C(CO)O)O)O)O)O)O	6
flopropione	C9H10O4	1	1	2	5	3	0	1	5	0	0	0.4444	182	448.65	335	0.7467	wangjchemphys06	wangjchemphys06	CCC(=O)C1=C(C=C(C=C1O)O)O	5B
2-methyl-1-propanol	C4H10O	2	1	1	0	1	0	0	0	0	0	0.2500	74	171.25	107	0.6248	wangjchemphys06	wangjchemphys06	CC(C)CO	
2-methyl-1-propanol	C4H10O	2	1	1	0	1	0	0	0	0	0	0.2500	74	171.25	116.5	0.6803	murthyjchemsocfaradaytrans93	murthyjchemsocfaradaytrans93	CC(C)CO	
2-methyl-1-propanol	C4H10O	2	1	1	0	1	0	0	0	0	0	0.2500	74	171.25	127	0.7416	koleskepolymengsci79	koleskepolymengsci79	CC(C)CO	
n-hexanol	C6H14O	1	5	0	0	1	0	0	0	0	0	0.1667	102	221	125	0.5656	naitojphychem93	naitojphychem93	CCCCCCO	
n-pentanol	C5H12O	1	4	0	0	1	0	0	0	0	0	0.2000	88	195	120	0.6154	naitojphychem93	naitojphychem93	CCCCCO	
benzyl alcohol	C7H8O	0	1	5	1	1	0	0	4	0	0	0.1429	108	258	168	0.6512	naitojphychem93	naitojphychem93	c1ccc(cc1)CO	
propan-1,2-diol	C3H8O2	1	1	1	0	2	0	0	0	0	0	0.6667	76	217	160	0.7373	naitojphychem93	naitojphychem93	CC(O)CO	
propan-1,2-diol	C3H8O2	1	1	1	0	2	0	0	0	0	0	0.6667	76	213.15	169.2	0.7938	wangjchemphys08	wangjchemphys08	CC(O)CO	
propan-1,2-diol	C3H8O2	1	1	1	0	2	0	0	0	0	0	0.6667	76	213.15	169	0.7929	johariannnyacadsci76	johariannnyacadsci76	CC(O)CO	
resorcinol	C6H6O2	0	0	4	2	2	0	0	4	0	0	0.3333	110	383	250	0.6527	naitojphychem93	naitojphychem93	c1cc(cc(c1)O)O	
phenyl-salicylate	C13H10O3	0	0	9	4	1	1	1	9	0	0	0.2308	214	314	217	0.6911	naitojphychem93	naitojphychem93	O=C(Oc2ccccc2)c1c(O)cccc1	
phenyl-salicylate	C13H10O3	0	0	9	4	1	1	1	9	0	0	0.2308	214	316.2	221.6	0.7008	murthyjchemsocfaradaytrans93	murthyjchemsocfaradaytrans93	O=C(Oc2ccccc2)c1c(O)cccc1	
phenyl salicylate	C13H10O3	0	0	9	4	1	1	1	9	0	0	0.2308	214	316.15	220	0.6959	wangjchemphys06	wangjchemphys06	O=C(Oc2ccccc2)c1c(O)cccc1	
phenyl salicylate	C13H10O3	0	0	9	4	1	1	1	9	0	0	0.2308	214	316.15	213	0.6737	wangjchemphys06	cukierman73	O=C(Oc2ccccc2)c1c(O)cccc1	
dimethylphthalat	C10H10O4	2	0	4	4	0	2	2	6	0	0	0.4000	194	274	192	0.7007	naitojphychem93	naitojphychem93	O=C(OC)c1ccccc1C(=O)OC	5B
dimethylphthalat	C10H10O4	2	0	4	4	0	2	2	6	0	0	0.4000	194	274.4	194.9	0.7103	murthyjchemsocfaradaytrans93	murthyjchemsocfaradaytrans93	O=C(OC)c1ccccc1C(=O)OC	5B
dimethylphthalat	C10H10O4	2	0	4	4	0	2	2	6	0	0	0.4000	194	278.65	193	0.6926	nayakjchemphys93	nayakjchemphys93	O=C(OC)c1ccccc1C(=O)OC	5B
dimethylphthalat	C10H10O4	2	0	4	4	0	2	2	6	0	0	0.4000	194	278.65	193	0.6926	carpenterjchemphys67	carpenterjchemphys67	O=C(OC)c1ccccc1C(=O)OC	5B
diethylphthalat	C12H14O4	2	2	4	4	0	2	2	6	0	0	0.3333	222	270	180	0.6667	naitojphychem93	naitojphychem93	CCOC(=O)c1ccccc1C(=O)OCC	5A, 5B
diethylphthalat	C12H14O4	2	2	4	4	0	2	2	6	0	0	0.3333	222	273	182.5	0.6685	murthyjchemsocfaradaytrans93	murthyjchemsocfaradaytrans93	CCOC(=O)c1ccccc1C(=O)OCC	5A, 5B
diethylphthalat	C12H14O4	2	2	4	4	0	2	2	6	0	0	0.3333	222	232.65	178	0.7651	wangjchemphys06	wangjchemphys06	CCOC(=O)c1ccccc1C(=O)OCC	5A, 5B
diethylphthalat	C12H14O4	2	2	4	4	0	2	2	6	0	0	0.3333	222	232.65	180.8	0.7771	nayakjchemphys93	nayakjchemphys93	CCOC(=O)c1ccccc1C(=O)OCC	5A, 5B
2-ethyl-1-hexanol	C8H18O	2	5	1	0	1	0	0	0	0	0	0.1250	130	203.15	148.9	0.7330	murthyjphyschem96	murthyjphyschem96	CCCCC(CC)CO	
5-methyl-3-heptanol	C8H18O	3	3	2	0	1	0	0	0	0	0	0.1250	130	181.95	159	0.8739	murthyjphyschem96	murthyjphyschem96	CCC(C)CC(CC)O	
2-phenyl-1-ethanol	C8H10O	0	2	5	1	1	0	0	4	0	0	0.1250	122	246.15	181.4	0.7369	murthyjphyschem96	murthyjphyschem96	c1ccc(cc1)CCO	
2-butanol	C4H10O	2	1	1	0	1	0	0	0	0	0	0.2500	74	184.65	127	0.6878	koleskepolymengsci79	koleskepolymengsci79	CCC(C)O	
2-butanol	C4H10O	2	1	1	0	1	0	0	0	0	0	0.2500	74	184.65	120.34	0.6517	murthyjchemsocfaradaytrans93	murthyjchemsocfaradaytrans93	CCC(C)O	
2-butanol	C4H10O	2	1	1	0	1	0	0	0	0	0	0.2500	74	171.25	116	0.6774	johariannnyacadsci76	johariannnyacadsci76	CCC(C)O	
2-butanol	C4H10O	2	1	1	0	1	0	0	0	0	0	0.2500	74	158	100	0.6329	naitojphychem93	naitojphychem93	CCC(C)O	
2,2-dimethyl-1-propanol	C5H12O	3	1	0	1	1	0	0	0	0	0	0.2000	88	325.65	166	0.5097	koleskepolymengsci79	koleskepolymengsci79	CC(CO)(C)C	
o-xylene	C8H10	2	0	4	2	0	0	0	4	0	0	0.0000	106	247.95	123	0.4961	wangjphyschemb07	wangjphyschemb07	CC1=C(C)C=CC=C1	
m-xylene	C8H10	2	0	4	2	0	0	0	4	0	0	0.0000	106	225.35	120	0.5325	wangjphyschemb07	wangjphyschemb07	CC1=CC(=CC=C1)C	
m-xylene	C8H10	2	0	4	2	0	0	0	4	0	0	0.0000	106	225.35	125.5	0.5569	wangjchemphys06	wangjchemphys06	CC1=CC(=CC=C1)C	
2-methylpentane	C6H14	3	2	1	0	0	0	0	0	0	0	0.0000	86	120	80	0.6667	naitojphychem93	naitojphychem93	CCCC(C)C	5A
2-methylpentane	C6H14	3	2	1	0	0	0	0	0	0	0	0.0000	86	119.55	78	0.6524	wangjchemphys06	wangjchemphys06	CCCC(C)C	5A
triphenylethene	C20H16	0	0	16	4	0	0	0	13	0	0	0.0000	256	345.65	246	0.7117	wangjchemphys06	wangjchemphys06	C1=CC=C(C=C1)C=C(C2=CC=CC=C2)C3=CC=CC=C3	6
n-buten	C4H8	1	2	1	0	0	0	0	1	0	0	0.0000	56	88	61	0.6932	naitojphychem93	naitojphychem93	C=CCC	
n-buten	C4H8	1	2	1	0	0	0	0	1	0	0	0.0000	56	87.81	58	0.6605	wangjchemphys06	wangjchemphys06	C=CCC	
4-methylstyrene	C9H10	1	1	5	2	0	0	0	5	0	0	0.0000	118	239.05	167	0.6986	wangjchemphys06	wangjchemphys06	CC1=CC=C(C=C1)C=C	
o-terphenyl	C18H14	0	0	14	4	0	0	0	12	0	0	0.0000	230	329	244	0.7416	tiersthermochimact93	tiersthermochimact93	C1=CC=C(C=C1)C2=CC=CC=C2C3=CC=CC=C3	
toluol	C7H8	1	0	5	1	0	0	0	4	0	0	0.0000	92	177.6	119.4	0.6723	murthyjchemsocfaradaytrans93	murthyjchemsocfaradaytrans93	Cc1ccccc1	
toluol	C7H8	1	0	5	1	0	0	0	4	0	0	0.0000	92	178.2	113	0.6341	carpenterjchemphys67	carpenterjchemphys67	Cc1ccccc1	
toluol	C7H8	1	0	5	1	0	0	0	4	0	0	0.0000	92	178.2	117	0.6566	wangjchemphys06	wangjchemphys06	Cc1ccccc1	
toluol	C7H8	1	0	5	1	0	0	0	4	0	0	0.0000	92	178	113	0.6348	naitojphychem93	naitojphychem93	Cc1ccccc1	
toluol	C7H8	1	0	5	1	0	0	0	4	0	0	0.0000	92	178.2	117.25	0.6580	lesikarjchemphys77	lesikarjchemphys77	Cc1ccccc1	
ethylbenzene	C8H10	1	1	5	1	0	0	0	4	0	0	0.0000	106	178.19	115	0.6454	wangjchemphys06	wangjchemphys06	CCc1ccccc1	
ethylbenzene	C8H10	1	1	5	1	0	0	0	4	0	0	0.0000	106	178.19	111	0.6229	carpenterjchemphys67	carpenterjchemphys67	CCc1ccccc1	
ethylbenzene	C8H10	1	1	5	1	0	0	0	4	0	0	0.0000	106	178	111	0.6236	naitojphychem93	naitojphychem93	CCc1ccccc1	
propylbenzene	C9H12	1	2	5	1	0	0	0	4	0	0	0.0000	120	173.6	126.2	0.7270	murthyjchemsocfaradaytrans93	murthyjchemsocfaradaytrans93	CCCC1=CC=CC=C1	
n-pentylbenzene	C11H16	1	4	5	1	0	0	0	4	0	0	0.0000	148	194	128	0.6598	naitojphychem93	naitojphychem93	CCCCCC1=CC=CC=C1	
n-pentylbenzene	C11H16	1	4	5	1	0	0	0	4	0	0	0.0000	148	198.15	128	0.6460	carpenterjchemphys67	carpenterjchemphys67	CCCCCC1=CC=CC=C1	
n-hexylbenzene	C12H18	1	5	5	1	0	0	0	4	0	0	0.0000	162	212.15	137	0.6458	carpenterjchemphys67	carpenterjchemphys67	CCCCCCC1=CC=CC=C1	6
n-hexylbenzene	C12H18	1	5	5	1	0	0	0	4	0	0	0.0000	162	205	137	0.6683	naitojphychem93	naitojphychem93	CCCCCCC1=CC=CC=C1	6
sec-butylbenzene	C10H14	2	1	6	1	0	0	0	4	0	0	0.0000	134	190.45	127	0.6668	carpenterjchemphys67	carpenterjchemphys67	CCC(C)C1=CC=CC=C1	
sec-butylbenzene	C10H14	2	1	6	1	0	0	0	4	0	0	0.0000	134	198	128	0.6465	naitojphychem93	naitojphychem93	CCC(C)C1=CC=CC=C1	
tert-butyl-benzene	C10H14	3	0	5	2	0	0	0	4	0	0	0.0000	134	215.35	142	0.6594	carpenterjchemphys67	carpenterjchemphys67	CC(C)(C)C1=CC=CC=C1	
tert-butyl-benzene	C10H14	3	0	5	2	0	0	0	4	0	0	0.0000	134	215	142	0.6605	naitojphychem93	naitojphychem93	CC(C)(C)C1=CC=CC=C1	

Name	Formula	#CH3	#CH2	#CH	#C	#OH	#C-O-C	#O=C	DBA	#N	#Hal	O:C	M / g/mol	Tm / K	Tg / K	Tg/Tm	Reference(Tm)	Reference(Tg)	SMILES	Figure
3-methylheptane	C8H18	3	4	1	0	0	0	0	0	0	0	0.0000	114	150	98	0.6533	naitojphychem93	naitojphychem93	CCCCC(C)CC	
3-methylheptane	C8H18	3	4	1	0	0	0	0	0	0	0	0.0000	114	152.67	107	0.7009	koleskepolymengsci79	koleskepolymengsci79	CCCCC(C)CC	
cyclohexene	C6H10	0	4	2	0	0	0	0	2	0	0	0.0000	82	170	92	0.5412	naitojphychem93	naitojphychem93	C1CCC=CC1	
methylcyclohexane	C7H14	1	5	1	0	0	0	0	1	0	0	0.0000	98	146.55	85	0.5800	carpenterjchemphys67	carpenterjchemphys67	CC1CCCCC1	
methylcyclohexane	C7H14	1	5	1	0	0	0	0	1	0	0	0.0000	98	147	85	0.5782	naitojphychem93	naitojphychem93	CC1CCCCC1	
ethylcyclohexane	C8H16	1	6	1	0	0	0	0	1	0	0	0.0000	112	161.9	98	0.6053	carpenterjchemphys67	carpenterjchemphys67	CCC1CCCCC1	
ethylcyclohexane	C8H16	1	6	1	0	0	0	0	1	0	0	0.0000	112	162	98	0.6049	naitojphychem93	naitojphychem93	CCC1CCCCC1	
isopropylcyclohexane	C9H18	2	5	2	0	0	0	0	1	0	0	0.0000	126	183.8	108	0.5876	carpenterjchemphys67	carpenterjchemphys67	CC(C)C1CCCCC1	5B
sec-butylcyclohexane	C10H20	2	6	2	0	0	0	0	1	0	0	0.0000	140	178.4	123	0.6895	ramos2014	carpenterjchemphys67	CCC(C)C1CCCCC1	
n-butylcyclohexane	C10H20	1	8	1	0	0	0	0	1	0	0	0.0000	140	198.42	119	0.5997	carpenterjchemphys67	carpenterjchemphys67	CCCCC1CCCCC1	
n-butylcyclohexane	C10H20	1	8	1	0	0	0	0	1	0	0	0.0000	140	198	119	0.6010	naitojphychem93	naitojphychem93	CCCCC1CCCCC1	
n-pentylcyclohexane	C11H22	1	9	1	0	0	0	0	1	0	0	0.0000	154	215.65	125	0.5796	carpenterjchemphys67	carpenterjchemphys67	CCCCCC1CCCCC1	
n-hexylcyclohexane	C12H24	1	10	1	0	0	0	0	1	0	0	0.0000	168	230.15	133	0.5779	carpenterjchemphys67	carpenterjchemphys67	CCCCCCC1CCCCC1	
trans-2-hexene	C6H12	2	2	2	0	0	0	0	1	0	0	0.0000	84	140.15	85	0.6065	carpenterjchemphys67	carpenterjchemphys67	CCCC=CC	6
1-heptene	C7H14	1	5	1	0	0	0	0	1	0	0	0.0000	98	154.25	91	0.5900	carpenterjchemphys67	carpenterjchemphys67	CCCCCC=C	
cis-2-octene	C8H16	2	4	2	0	0	0	0	1	0	0	0.0000	112	172.95	101	0.5840	carpenterjchemphys67	carpenterjchemphys67	CCCCCC=CC	
3-methylhexane	C7H16	3	3	1	0	0	0	0	0	0	0	0.0000	100	154	80	0.5195	naitojphychem93	naitojphychem93	CCCC(C)CC	
3-methylhexane	C7H16	3	3	1	0	0	0	0	0	0	0	0.0000	100	153.75	99	0.6439	koleskepolymengsci79	koleskepolymengsci79	CCCC(C)CC	
4-methylnonane	C10H22	3	6	1	0	0	0	0	0	0	0	0.0000	142	174.15	123	0.7063	koleskepolymengsci79	koleskepolymengsci79	CCCCCC(C)CCC	
2-isopropoxyethanol	C5H12O2	2	2	1	0	1	1	0	0	0	0	0.4000	104	191.58	141	0.7360	wangjphyschemb07	wangjphyschemb07	CC(C)OCCO	
2,5-hexanediol	C6H14O2	2	2	2	0	2	0	0	0	0	0	0.3333	118	316.15	203.5	0.6437	wangjphyschemb07	wangjphyschemb07	CC(CCC(C)O)O	
n-eugenol	C10H12O2	1	2	4	3	1	1	0	5	0	0	0.2000	164	265.65	192.7	0.7254	wangjphyschemb07	wangjphyschemb07	COC1=C(C=CC(=C1)CC=C)O	
sorbitol	C6H14O6	0	2	4	0	6	0	0	0	0	0	1.0000	182	384.15	268	0.6976	wangjchemphys06	wangjchemphys06	C(C(C(C(C(CO)O)O)O)O)O	
sorbitol	C6H14O6	0	2	4	0	6	0	0	0	0	0	1.0000	182	367	271	0.7384	kercthermochimacta95	kercthermochimacta95	C(C(C(C(C(CO)O)O)O)O)O	
sorbitol	C6H14O6	0	2	4	0	6	0	0	0	0	0	1.0000	182	384	270	0.7031	kercthermochimacta95	kercthermochimacta95	C(C(C(C(C(CO)O)O)O)O)O	
propylencarbonate	C4H6O3	1	1	1	1	0	2	1	2	0	0	0.7500	102	224.35	159.5	0.7109	wangjchemphys06	wangjchemphys06	CC1COC(=O)O1	
guajacol	C7H8O2	1	0	4	2	1	1	0	4	0	0	0.2857	124	303	205	0.6766	tyllijmolecstruct90	tyllijmolecstruct90	COC1=CC=CC=C1O	
sucrose	C12H22O11	0	3	8	1	8	3	0	2	0	0	0.9167	342	458	319	0.6965	tiersthermochimact93	tiersthermochimact93	C(C1C(C(C(C(O1)OC2(C(C(C(O2)CO)O)O)CO)O)O)O)O	
sucrose	C12H22O11	0	3	8	1	8	3	0	2	0	0	0.9167	342	453	348	0.7682	hancockjpharmsci97	hancockjpharmsci97	C(C1C(C(C(C(O1)OC2(C(C(C(O2)CO)O)O)CO)O)O)O)O	
sucrose	C12H22O11	0	3	8	1	8	3	0	2	0	0	0.9167	342	458.65	345	0.7522	wangjchemphys06	wangjchemphys06	C(C1C(C(C(C(O1)OC2(C(C(C(O2)CO)O)O)CO)O)O)O)O	
sucrose	C12H22O11	0	3	8	1	8	3	0	2	0	0	0.9167	342	465.7	334	0.7172	murthyjchemsocfaradaytrans93	murthyjchemsocfaradaytrans93	C(C1C(C(C(C(O1)OC2(C(C(C(O2)CO)O)O)CO)O)O)O)O	
sucrose	C12H22O11	0	3	8	1	8	3	0	2	0	0	0.9167	342	453	329	0.7263	kercthermochimacta95	kercthermochimacta95	C(C1C(C(C(C(O1)OC2(C(C(C(O2)CO)O)O)CO)O)O)O)O	
trehalose	C12H22O11	0	2	10	0	8	3	0	2	0	0	0.9167	342	476	385	0.8088	hancockjpharmsci97	hancockjpharmsci97	C(C1C(C(C(C(O1)OC2C(C(C(C(O2)CO)O)O)O)O)O)O)O	
dioctylphthalat	C24H38O4	2	14	4	4	0	2	2	6	0	0	0.1667	390	298.15	182.5	0.6121	nayakjchemphys93	nayakjchemphys93	CCCCCCCCOC(=O)C1=CC=CC=C1C(=O)OCCCCCCCC	
dioctylphthalat	C24H38O4	2	14	4	4	0	2	2	6	0	0	0.1667	390	298.15	184.4	0.6185	murthyjchemsocfaradaytrans93	murthyjchemsocfaradaytrans93	CCCCCCCCOC(=O)C1=CC=CC=C1C(=O)OCCCCCCCC	
naphthylsalyciate	C17H12O3	0	0	11	6	1	1	1	12	0	0	0.1765	264	368	244	0.6630	naitojphychem93	naitojphychem93	C1=CC=C2C(=C1)C=CC=C2OC(=O)C3=CC=CC=C3O	
vinylacetate	C4H6O2	1	1	1	1	0	1	1	2	0	0	0.5000	86	180	125	0.6944	naitojphychem93	naitojphychem93	CC(=O)OC=C	
xylitol	C5H12O5	0	2	3	0	2	0	0	0	0	0	0.4000	104	366.65	244	0.6655	wangjchemphys06	wangjchemphys06	C(C(C(C(CO)O)O)O)O	5A, 6
methyl(s)(-)lactate	C4H8O3	2	0	1	1	1	1	1	1	0	0	0.7500	104	396	286	0.7222	tiersthermochimact93	tiersthermochimact93	CC(C(=O)OC)O	
(s)-glycerol acetonate	C6H12O3	2	2	1	1	1	2	0	1	0	0	0.5000	132	384	282	0.7344	tiersthermochimact93	tiersthermochimact93	CC1(OCC(O1)CO)C	5B
(r)-nopol	C11H18O	2	4	3	2	1	0	0	3	0	0	0.0909	166	348	277	0.7960	tiersthermochimact93	tiersthermochimact93	CC1(C)[C@H]2CC=C(CCO)[C@@H]1C2	
3-hydroxy-2-methylpropionate	C5H10O3	2	1	1	1	1	1	1	1	0	0	0.6000	118	376	269	0.7154	tiersthermochimact93	tiersthermochimact93	CC(CO)C(=O)OC	
dianin’s compound	C18H20O2	3	1	8	6	1	1	0	9	0	0	0.1111	268	430	299	0.6953	tiersthermochimact93	tiersthermochimact93	CC1(CC(C2=CC=CC=C2O1)(C)C3=CC=C(C=C3)O)COc1ccc(cc1)C2(c3c(OC(C2)(C)C)cccc3)C	
4-(1-idianyl)phenol	C15H14O	0	2	9	4	1	0	0	9	0	0	0.0667	210	363	269	0.7410	tiersthermochimact93	tiersthermochimact93	C1CC2=CC=CC=C2C1C3=CC=C(C=C3)O	
2p-hydroxyphenoxytetrahydropyran	C11H14O3	0	4	5	2	1	2	0	5	0	0	0.2727	194	358	255	0.7123	tiersthermochimact93	tiersthermochimact93	C1CCOC(C1)OC2=CC=CC=C2O	
salicin	C13H18O7	0	2	9	2	5	2	0	5	0	0	0.5385	286	471	333.5	0.7081	tiersthermochimact93	tiersthermochimact93	C1=CC=C(C(=C1)CO)OC2C(C(C(C(O2)CO)O)O)O	
salicin	C13H18O7	0	2	9	2	5	2	0	5	0	0	0.5385	286	466	333	0.7146	kercthermochimacta95	kercthermochimacta95	C1=CC=C(C(=C1)CO)OC2C(C(C(C(O2)CO)O)O)O	
cholesterol	C27H46O	5	11	8	3	1	0	0	5	0	0	0.0370	386	422	332	0.7867	tiersthermochimact93	tiersthermochimact93	CC(C)CCCC(C)C1CCC2C1(CCC3C2CC=C4C3(CCC(C4)O)C)C	
beta-d-glucosepentaacetate	C16H22O11	5	1	5	5	0	6	5	6	0	0	0.6875	390	405	291	0.7185	tiersthermochimact93	tiersthermochimact93	CC(=O)OCC1C(C(C(C(O1)OC(=O)C)OC(=O)C)OC(=O)C)OC(=O)C	
beta-d-glucosepentaacetate	C16H22O11	5	1	5	5	0	6	5	6	0	0	0.6875	390	386	296	0.7668	tiersthermochimact93	tiersthermochimact93	CC(=O)OCC1C(C(C(C(O1)OC(=O)C)OC(=O)C)OC(=O)C)OC(=O)C	
3-pentadecylcyclohexanone	C21H40O	1	18	1	1	0	0	1	2	0	0	0.0476	308	313	225	0.7188	tiersthermochimact93	tiersthermochimact93	CCCCCCCCCCCCCCCC1CCCC(=O)C1	
phenolphthalein	C20H14O4	0	0	12	8	2	1	1	14	0	0	0.2000	318	534	340	0.6367	beamanjpolysci52	beamanjpolysci52	O=C1OC(C2=C1C=CC=C2)(C3=CC=C(C=C3)O[H])C4=CC=C(C=C4)O[H]	
phenolphthalein	C20H14O4	0	0	12	8	2	1	1	14	0	0	0.2000	318	535	365	0.6822	tiersthermochimact93	tiersthermochimact93	O=C1OC(C2=C1C=CC=C2)(C3=CC=C(C=C3)O[H])C4=CC=C(C=C4)O[H]	
4,4’-dimethoxytetraphenylmethane	C27H24O2	2	0	18	7	0	2	0	16	0	0	0.0741	380	452	297	0.6571	tiersthermochimact93	tiersthermochimact93	COc4ccc(C(c1ccccc1)(c2ccccc2)c3ccc(OC)cc3)cc4	6
lactic acid	C3H6O3	1	0	1	1	2	0	1	1	0	0	1.0000	90	299	204	0.6823	naitojphychem93	naitojphychem93	CC(C(=O)O)O	
lactic acid	C3H6O3	1	0	1	1	2	0	1	1	0	0	1.0000	90	291	200	0.6873	beamanjpolysci52	beamanjpolysci52	CC(C(=O)O)O	
propylencarbonate	C4H6O3	1	1	1	1	0	2	1	2	0	0	0.7500	102	218	160	0.7339	naitojphychem93	naitojphychem93	CC1COC(=O)O1	
epoxypropane	C3H6O	1	1	1	0	0	1	0	1	0	0	0.3333	58	160.2	114.7	0.7160	murthyjchemsocfaradaytrans93	murthyjchemsocfaradaytrans93	CC1CO1	
n-butylacetate	C6H12O2	2	3	0	1	0	1	1	1	0	0	0.3333	116	194.3	125.7	0.6469	murthyjchemsocfaradaytrans93	murthyjchemsocfaradaytrans93	CCCCOC(=O)C	
camphor	C10H16O	3	3	2	3	0	0	1	3	0	0	0.0909	166	213	156.7	0.7357	murthyjchemsocfaradaytrans93	murthyjchemsocfaradaytrans93	CC1(C2CCC1(C(=O)C2)C)C	5A
dibutylphthalat	C16H22O4	2	6	4	2	0	2	2	6	0	0	0.2857	250	238.15	178.7	0.7504	murthyjchemsocfaradaytrans93	murthyjchemsocfaradaytrans93	CCCCOC(=O)C1=CC=CC=C1C(=O)OCCCC	
dibutylphthalat	C16H22O4	2	6	4	2	0	2	2	6	0	0	0.2857	250	238.15	176	0.7390	carpenterjchemphys67	carpenterjchemphys67	CCCCOC(=O)C1=CC=CC=C1C(=O)OCCCC	
dibutylphthalat	C16H22O4	2	6	4	2	0	2	2	6	0	0	0.2857	250	238.15	178.5	0.7495	wangjchemphys08	wangjchemphys08	CCCCOC(=O)C1=CC=CC=C1C(=O)OCCCC	
dibutylphthalat	C16H22O4	2	6	4	2	0	2	2	6	0	0	0.2857	250	238.15	177.4	0.7449	wangjphyschemb07	wangjphyschemb07	CCCCOC(=O)C1=CC=CC=C1C(=O)OCCCC	
dibutylphthalat	C16H22O4	2	6	4	2	0	2	2	6	0	0	0.2857	250	238.15	176	0.7390	nayakjchemphys93	nayakjchemphys93	CCCCOC(=O)C1=CC=CC=C1C(=O)OCCCC	
o-cresol	C13H12O	0	1	9	3	1	0	0	8	0	0	0.0769	184	325.6	220.1	0.6760	murthyjchemsocfaradaytrans93	murthyjchemsocfaradaytrans93	CC1=CC=CC=C1O	
isopropranol	C3H8O	2	0	1	0	1	0	0	0	0	0	0.3333	60	185.25	118.8	0.6413	murthyjchemsocfaradaytrans93	murthyjchemsocfaradaytrans93	CC(C)O	
isopropranol	C3H8O	2	0	1	0	1	0	0	0	0	0	0.3333	60	185.25	121	0.6532	koleskepolymengsci79	koleskepolymengsci79	CC(C)O	
1,3-butanediol	C4H10O2	1	2	1	0	2	0	0	0	0	0	0.5000	90	262.76	175.4	0.6675	mariajthermanalcalorim10	mariajthermanalcalorim10	CC(CCO)O	
1,3-butanediol	C4H10O2	1	2	1	0	2	0	0	0	0	0	0.5000	90	262.76	175.6	0.6683	mariajthermanalcalorim10	mariajthermanalcalorim10	CC(CCO)O	
2,3-butanediol	C4H10O2	2	0	2	0	2	0	0	0	0	0	0.5000	90	291.3	195.1	0.6698	mariajthermanalcalorim10	mariajthermanalcalorim10	CC(C(C)O)O	
m-cresol	C7H8O	1	0	4	1	1	0	0	4	0	0	0.1667	94	285	198	0.6947	albasimionescojchemphys99	albasimionescojchemphys99	CC1=CC(=CC=C1)O	
mephenesin	C10H14O3	1	2	5	2	2	1	0	4	0	0	0.3000	182	340	247	0.7265	fukuokachempharmbull91	fukuokachempharmbull91	CC1=CC=CC=C1OCC(CO)O	
mephenesin	C10H14O3	1	2	5	2	2	1	0	4	0	0	0.3000	182	340	247	0.7265	kercthermochimacta95	kercthermochimacta95	CC1=CC=CC=C1OCC(CO)O	
estradiol-17b-cypionate	C26H36O3	1	12	8	5	1	1	1	9	0	0	0.1154	396	425	309	0.7271	fukuokachempharmbull91	fukuokachempharmbull91	CC12CCC3C(C1CCC2OC(=O)CCC4CCCC4)CCC5=C3C=CC(=C5)O	5A
estradiol-17b-cypionate	C26H36O3	1	12	8	5	1	1	1	9	0	0	0.1154	396	425	309	0.7271	kercthermochimacta95	kercthermochimacta95	CC12CCC3C(C1CCC2OC(=O)CCC4CCCC4)CCC5=C3C=CC(=C5)O	5A
norethylnodrel	C20H26O2	1	9	4	6	1	0	1	8	0	0	0.1000	298	453	324	0.7152	fukuokachempharmbull91	fukuokachempharmbull91	CC12CCC3C(C1CCC2(C#C)O)CCC4=C3CCC(=O)C4	5A
norethylnodrel	C20H26O2	1	9	4	6	1	0	1	8	0	0	0.1000	298	453	324	0.7152	kercthermochimacta95	kercthermochimacta95	CC12CCC3C(C1CCC2(C#C)O)CCC4=C3CCC(=O)C4	5A
beta-estradiol-3-benzoate	C25H28O3	1	6	12	6	1	1	1	12	0	0	0.1200	376	472	336	0.7119	fukuokachempharmbull91	fukuokachempharmbull91	CC12CCC3C(C1CCC2O)CCC4=C3C=CC(=C4)OC(=O)C5=CC=CC=C5	
beta-estradiol-3-benzoate	C25H28O3	1	6	12	6	1	1	1	12	0	0	0.1200	376	472	336	0.7119	kercthermochimacta95	kercthermochimacta95	CC12CCC3C(C1CCC2O)CCC4=C3C=CC(=C4)OC(=O)C5=CC=CC=C5	
ursodeoxycholic acid	C24H40O4	3	10	8	3	3	0	1	5	0	0	0.1667	392	477	378	0.7925	fukuokachempharmbull91	fukuokachempharmbull91	CC(CCC(=O)O)C1CCC2C1(CCC3C2C(CC4C3(CCC(C4)O)C)O)C	
ursodeoxycholic acid	C24H40O4	3	10	8	3	3	0	1	5	0	0	0.1667	392	477	378	0.7925	kercthermochimacta95	kercthermochimacta95	CC(CCC(=O)O)C1CCC2C1(CCC3C2C(CC4C3(CCC(C4)O)C)O)C	
deoxycholic acid	C24H40O4	3	10	8	3	3	0	1	5	0	0	0.1667	392	447	377	0.8434	fukuokachempharmbull91	fukuokachempharmbull91	CC(CCC(=O)O)C1CCC2C1(C(CC3C2CCC4C3(CCC(C4)O)C)O)C	
deoxycholic acid	C24H40O4	3	10	8	3	3	0	1	5	0	0	0.1667	392	447	377	0.8434	kercthermochimacta95	kercthermochimacta95	CC(CCC(=O)O)C1CCC2C1(C(CC3C2CCC4C3(CCC(C4)O)C)O)C	
cholic acid	C24H40O5	3	9	9	3	4	0	1	5	0	0	0.2083	408	473	393	0.8309	fukuokachempharmbull91	fukuokachempharmbull91	CC(CCC(=O)O)C1CCC2C1(C(CC3C2C(CC4C3(CCC(C4)O)C)O)O)C	
cholic acid	C24H40O5	3	9	9	3	4	0	1	5	0	0	0.2083	408	473	393	0.8309	kercthermochimacta95	kercthermochimacta95	CC(CCC(=O)O)C1CCC2C1(C(CC3C2C(CC4C3(CCC(C4)O)C)O)O)C	
lactose	C12H22O11	0	2	10	0	8	3	0	2	0	0	0.9167	342	486	383	0.7881	hancockjpharmsci97	hancockjpharmsci97	C(C1C(C(C(C(O1)OC(C(CO)O)C(C(C=O)O)O)O)O)O)O	6
diethylhexylphthalat	C24H38O4	4	10	6	4	0	2	2	6	0	0	0.1667	390	218.15	184	0.8435	carpenterjchemphys67	carpenterjchemphys67	O=C(OCC(CC)CCCC)C1=CC=CC=C1C(OCC(CC)CCCC)=O	
cholecalciterol	C27H44O	4	12	7	4	1	0	0	6	0	0	0.0370	384	352	296	0.8409	kercthermochimacta95	kercthermochimacta95	CC(C)CCCC(C)C1CCC2C1(CCCC2=CC=C3CC(CCC3=C)O)C	
diethylstilbestrol	C18H20O2	2	2	8	6	2	0	0	9	0	0	0.1111	268	439	308	0.7016	kercthermochimacta95	kercthermochimacta95	CCC(=C(CC)C1=CC=C(C=C1)O)C2=CC=C(C=C2)O	
dehydrocholic acid	C24H34O5	3	9	6	6	1	0	4	8	0	0	0.2083	402	502	348	0.6932	kercthermochimacta95	kercthermochimacta95	CC(CCC(=O)O)C1CCC2C1(C(=O)CC3C2C(=O)CC4C3(CCC(=O)C4)C)C	

Name	Formula	#CH3	#CH2	#CH	#C	#OH	#C-O-C	#O=C	DBA	#N	#Hal	O:C	M / g/mol	Tm / K	Tg / K	Tg/Tm	Reference(Tm)	Reference(Tg)	SMILES	Figure
cellobiose	C12H22O11	0	2	10	0	8	3	0	2	0	0	0.9167	342	498	350	0.7028	kercthermochimacta95	kercthermochimacta95	C(C1C(C(C(C(O1)OC2C(OC(C(C2O)O)O)CO)O)O)O)O	6
17b-estradiol	C18H24O2	1	6	7	4	2	0	0	7	0	0	0.1111	272	445	354	0.7955	kercthermochimacta95	kercthermochimacta95	CC12CCC3C(C1CCC2O)CCC4=C3C=CC(=C4)O	6
aspirin	C9H8O4	1	0	4	4	1	1	1	6	0	0	0.3333	164	408	243	0.5956	kercthermochimacta95	kercthermochimacta95	CC(=O)OC1=CC=CC=C1C(=O)O	
methyltestosterone	C20H30O2	3	8	4	5	1	0	1	6	0	0	0.1000	302	421	270	0.6413	kercthermochimacta95	kercthermochimacta95	CC12CCC(=O)C=C1CCC3C2CCC4(C3CCC4(C)O)C	
progesterone	C21H30O2	3	8	5	5	0	0	2	7	0	0	0.0952	314	399	279	0.6992	kercthermochimacta95	kercthermochimacta95	CC(=O)C1CCC2C1(CCC3C2CCC4=CC(=O)CCC34C)C	
citric acid	C6H8O7	0	2	0	4	4	0	3	3	0	0	1.1667	192	432	283	0.6551	kercthermochimacta95	kercthermochimacta95	C(C(=O)O)C(CC(=O)O)(C(=O)O)O	
ergocalciferol	C28H44O	5	9	10	4	1	0	0	7	0	0	0.0357	396	376	290	0.7713	kercthermochimacta95	kercthermochimacta95	CC(C)C(C)C=CC(C)C1CCC2C1(CCCC2=CC=C3CC(CCC3=C)O)C	5A
n-nonanol	C9H20O	1	8	0	0	1	0	0	0	0	0	0.1111	144	268.15	153	0.5706	koleskepolymengsci79	koleskepolymengsci79	CCCCCCCCCO	
2-pentanol	C5H12O	2	2	1	0	1	0	0	0	0	0	0.2000	88	200.15	140	0.6995	koleskepolymengsci79	koleskepolymengsci79	CCCC(C)O	
3-pentanol	C5H12O	2	2	1	0	1	0	0	0	0	0	0.2000	88	204.15	143	0.7005	koleskepolymengsci79	koleskepolymengsci79	CCC(CC)O	
2-octanol	C8H18O	2	5	1	0	1	0	0	0	0	0	0.1250	130	241.55	166	0.6872	koleskepolymengsci79	koleskepolymengsci79	CCCCCCC(C)O	
4-hetpanol	C7H16O	2	4	1	0	1	0	0	0	0	0	0.1429	116	231.95	166	0.7157	koleskepolymengsci79	koleskepolymengsci79	CCCC(CCC)O	
4-decanol	C10H22O	2	7	1	0	1	0	0	0	0	0	0.1000	158	262.15	181	0.6904	koleskepolymengsci79	koleskepolymengsci79	CCCCCCC(CCC)O	
3-methyl-4-octanol	C9H20O	3	4	2	0	1	0	0	0	0	0	0.1111	144	221.51	180	0.8126	koleskepolymengsci79	koleskepolymengsci79	CCCCC(C(C)CC)O	
2-methyl-2-propanol	C4H10O	3	1	0	1	1	0	0	0	0	0	0.2000	88	298	180	0.6040	naitojphychem93	naitojphychem93	CC(C)(C)O	
2-methyl-2-propanol	C4H10O	3	0	0	1	1	0	0	0	0	0	0.2500	74	298.84	180	0.6023	koleskepolymengsci79	koleskepolymengsci79	CC(C)(C)O	
2-methyl-2-propanol	C4H10O	3	0	0	1	1	0	0	0	0	0	0.2500	74	303	158	0.5215	murthyjchemsocfaradaytrans93	murthyjchemsocfaradaytrans93	CC(C)(C)O	
2-methyl-2-butanol	C5H12O	3	1	0	1	1	0	0	0	0	0	0.2000	88	264.05	154	0.5832	koleskepolymengsci79	koleskepolymengsci79	CCC(C)(C)O	
2-methyl-2-heptanol	C8H18O	3	4	0	1	1	0	0	0	0	0	0.1250	130	222.75	178	0.7991	koleskepolymengsci79	koleskepolymengsci79	CCCCCC(C)(C)O	6
4-methyl-4-heptanol	C8H18O	3	4	0	1	1	0	0	0	0	0	0.1250	130	191.15	170	0.8894	koleskepolymengsci79	koleskepolymengsci79	CCCC(C)(CCC)O	6
6-methyl-3-heptanol	C8H18O	3	3	2	0	1	0	0	0	0	0	0.1250	130	213.15	159	0.7460	johariannnyacadsci76	johariannnyacadsci76	CCC(CCC(C)C)O	
3-methyl-3-heptanol	C8H18O	3	4	0	1	1	0	0	0	0	0	0.1250	130	190.15	151	0.7941	johariannnyacadsci76	johariannnyacadsci76	CCCCC(C)(CC)O	6
6-methyl-1-heptanol	C8H18O	2	5	1	0	1	0	0	0	0	0	0.1250	130	167.15	140	0.8376	johariannnyacadsci76	johariannnyacadsci76	CC(C)CCCCCO	
diethylether	C4H10O	2	2	0	0	0	1	0	0	0	0	0.2500	74	156.95	92.55	0.5897	lesikarjchemphys77	lesikarjchemphys77	CCOCC	
diisopropylether	C6H14O	4	0	2	0	0	1	0	0	0	0	0.1667	102	187.75	101.35	0.5398	lesikarjchemphys77	lesikarjchemphys77	CC(C)OC(C)C	6
acetone	C3H6O	2	0	0	1	0	0	1	1	0	0	0.3333	58	178.45	100.15	0.5612	lesikarjchemphys77	lesikarjchemphys77	CC(=O)C	
methylethylketone	C4H8O	2	1	0	1	0	0	1	1	0	0	0.2500	72	186.51	111	0.5951	lesikarjchemphys77	lesikarjchemphys77	CCC(=O)C	6
methylisobutylketone	C6H12O	3	1	0	1	0	0	1	1	0	0	0.2000	86	189.15	119.85	0.6336	lesikarjchemphys77	lesikarjchemphys77	CC(C)CC(=O)C	5A, 6
ethyl acetate	C4H8O2	2	1	0	1	0	1	1	1	0	0	0.5000	88	189.35	117.65	0.6213	lesikarjchemphys77	lesikarjchemphys77	CCOC(=O)C	6
ethyl acetate	C4H8O2	2	1	0	1	0	1	1	1	0	0	0.5000	88	189.4	116	0.6125	murthyjchemsocfaradaytrans93	murthyjchemsocfaradaytrans93	CCOC(=O)C	6
benzaldehyde	C7H6O	0	0	6	1	0	0	1	5	0	0	0.1429	106	216.05	149	0.6897	lesikarjchemphys77	lesikarjchemphys77	C1=CC=C(C=C1)C=O	
acetophenone	C8H8O	1	0	5	2	0	0	1	5	0	0	0.1250	120	293.65	161	0.5483	lesikarjchemphys77	lesikarjchemphys77	CC(=O)C1=CC=CC=C1	
1,3,5-trinaphthylbenzene	C36H24	0	0	24	12	0	0	0	25	0	0	0.0000	456	472	342	0.7246	naitojphychem93	naitojphychem93	C1=CC=C2C(=C1)C=CC=C2C3=CC(=CC(=C3)C4=CC=CC5=CC=CC=C54)C6=CC=CC7=CC=CC=C76	
decalin	C10H18	0	8	2	0	0	0	0	2	0	0	0.0000	138	233.15	137.4	0.5893	wangjchemphys08	wangjchemphys08	C1CCC2CCCCC2C1	
decalin	C10H18	0	8	2	0	0	0	0	2	0	0	0.0000	138	233.15	137.4	0.5893	wangjchemphys06	wangjchemphys06	C1CCC2CCCCC2C1	
3-methoxy-1-butanol	C5H12O2	2	2	1	0	1	1	0	0	0	0	0.4000	104	214	146.9	0.6864	joback	wangjchemphys08	CC(CCO)OC	
3-methoxy-1-butanol	C5H12O2	2	2	1	0	1	1	0	0	0	0	0.4000	104	214	145.3	0.6790	joback	wangjphyschemb07	CC(CCO)OC	
propylencarbonate	C4H6O3	1	1	1	1	0	2	1	2	0	0	0.7500	102	224.35	159.5	0.7109	wangjchemphys08	wangjchemphys08	CC1COC(=O)O1	
2,4-pentanediol	C5H12O2	2	1	2	0	2	0	0	0	0	0	0.4000	104	318.15	205.9	0.6472	mellan62	wangjchemphys08	CC(CC(C)O)O	
2-methyl-1-butanol	C5H12O	2	2	1	0	1	0	0	0	0	0	0.2000	88	156	123.6	0.7923	wangjchemphys08	wangjchemphys08	CCC(C)CO	
o-terphenyl	C18H14	0	0	14	4	0	0	0	12	0	0	0.0000	230	329.35	245.9	0.7466	wangjchemphys08	wangjchemphys08	C1=CC=C(C=C1)C2=CC=CC=C2C3=CC=CC=C3	
o-terphenyl	C18H14	0	0	14	4	0	0	0	12	0	0	0.0000	230	329.35	246	0.7469	wangjchemphys06	wangjchemphys06	C1=CC=C(C=C1)C2=CC=CC=C2C3=CC=CC=C3	
o-terphenyl	C18H14	0	0	14	4	0	0	0	12	0	0	0.0000	230	331.5	247.7	0.7472	murthyjchemsocfaradaytrans93	murthyjchemsocfaradaytrans93	C1=CC=C(C=C1)C2=CC=CC=C2C3=CC=CC=C3	
o-terphenyl	C18H14	0	0	14	4	0	0	0	12	0	0	0.0000	230	329	245	0.7447	naitojphychem93	naitojphychem93	C1=CC=C(C=C1)C2=CC=CC=C2C3=CC=CC=C3	
2-ethyl-1-butanol	C6H14O	2	3	1	0	1	0	0	0	0	0	0.1667	102	221.15	131	0.5924	bradleydataset	wangjchemphys08	CCC(CC)CO	
3-methyl-2-pentanol	C6H14O	3	1	2	0	1	0	0	0	0	0	0.1667	102	188	143.8	0.7649	gestis1	wangjchemphys08	CCC(C)C(C)O	6
4-methyl-2-pentanol	C6H14O	3	1	2	0	1	0	0	0	0	0	0.1667	102	183.15	157.1	0.8578	murthyjphyschem96	murthyjphyschem96	CC(C)CC(C)O	
3-methyl-2-pentanol	C6H14O	3	1	2	0	1	0	0	0	0	0	0.1667	102	225	158	0.7022	yawshandbook	koleskepolymengsci79	CCC(C)C(C)O	6
5-methyl-2-hexanol	C7H16O	3	2	2	0	1	0	0	0	0	0	0.1429	116	199	152.1	0.7643	yawshandbook	wangjchemphys08	CC(C)CCC(C)O	
3-methyl-4-hexanol	C7H16O	3	2	2	0	1	0	0	0	0	0	0.1429	116	199	163	0.8191	joback	koleskepolymengsci79	CCC(C)C(CC)O	
6-methyl-3-heptanol	C8H18O	3	3	2	0	1	0	0	0	0	0	0.1250	130	213.15	159	0.7460	murthyjphyschem96	murthyjphyschem96	CCC(CCC(C)C)O	
4-methyl-3-heptanol	C8H18O	3	3	2	0	1	0	0	0	0	0	0.1250	130	211	162.8	0.7716	joback	murthyjphyschem96	CCCC(C)C(CC)O	6
3-methyl-4-heptanol	C8H18O	3	3	2	0	1	0	0	0	0	0	0.1250	130	212	173	0.8160	yawshandbook	koleskepolymengsci79	CCCC(C(C)CC)O	
6-methyl-2-heptanol	C8H18O	3	3	2	0	1	0	0	0	0	0	0.1250	130	188	159	0.8457	crchandbook	murthyjphyschem96	CC(C)CCCC(C)O	
4-methyl-1-heptanol	C8H18O	2	5	1	0	1	0	0	0	0	0	0.1250	130	212	138	0.6509	yawshandbook	johariannnyacadsci76	CCCC(C)CCCO	
2-methyl-1-heptanol	C8H18O	2	5	1	0	1	0	0	0	0	0	0.1250	130	161.15	144	0.8936	johariannnyacadsci76	johariannnyacadsci76	CCCCCC(C)CO	
2-methyl-3-heptanol	C8H18O	3	3	2	0	1	0	0	0	0	0	0.1250	130	188.15	158	0.8398	johariannnyacadsci76	johariannnyacadsci76	CCCCC(C(C)C)O	
4-methyl-3-heptanol	C8H18O	3	3	2	0	1	0	0	0	0	0	0.1250	130	211	162.2	0.7687	joback	wangjchemphys08	CCCC(C)C(CC)O	6
4-methyl-3-heptanol	C8H18O	3	3	2	0	1	0	0	0	0	0	0.1250	130	150.15	160	1.0656	johariannnyacadsci76	johariannnyacadsci76	CCCC(C)C(CC)O	6
5-methyl-3-heptanol	C8H18O	3	3	2	0	1	0	0	0	0	0	0.1250	130	181.95	159	0.8739	johariannnyacadsci76	johariannnyacadsci76	CCC(C)CC(CC)O	
4-methyl-3-heptanol	C8H18O	3	3	2	0	1	0	0	0	0	0	0.1250	130	150.15	162.2	1.0803	wangjphyschemb07	wangjphyschemb07	CCCC(C)C(CC)O	6
2-ethyl-1-hexanol	C8H18O	2	5	1	0	1	0	0	0	0	0	0.1250	130	203.15	146	0.7187	wangjphyschemb07	wangjphyschemb07	CCCCC(CC)CO	
2-ethyl-1-hexanol	C8H18O	2	5	1	0	1	0	0	0	0	0	0.1250	130	203.15	145.9	0.7182	wangjchemphys08	wangjchemphys08	CCCCC(CC)CO	
2-methylpentane	C6H14	3	2	1	0	0	0	0	0	0	0	0.0000	86	119.55	78	0.6524	wangjphyschemb07	wangjphyschemb07	CCCC(C)C	5A
3-methylpentane	C6H14	3	2	1	0	0	0	0	0	0	0	0.0000	86	110.25	88	0.7982	koleskepolymengsci79	koleskepolymengsci79	CCC(C)CC	
3-methylpentane	C6H14	3	2	1	0	0	0	0	0	0	0	0.0000	86	110.25	77	0.6984	wangjchemphys06	wangjchemphys06	CCC(C)CC	
3-methylpentane	C6H14	3	2	1	0	0	0	0	0	0	0	0.0000	86	110.25	77	0.6984	wangjphyschemb07	wangjphyschemb07	CCC(C)CC	
propylbenzene	C9H12	1	2	5	1	0	0	0	4	0	0	0.0000	120	173.55	122	0.7030	carpenterjchemphys67	carpenterjchemphys67	CCCC1=CC=CC=C1	
propylbenzene	C9H12	1	2	5	1	0	0	0	4	0	0	0.0000	120	173.55	122	0.7030	wangjphyschemb07	wangjphyschemb07	CCCC1=CC=CC=C1	
propylbenzene	C9H12	1	2	5	1	0	0	0	4	0	0	0.0000	120	174	122	0.7011	naitojphychem93	naitojphychem93	CCCC1=CC=CC=C1	
isopropylbenzene	C9H12	2	0	6	1	0	0	0	4	0	0	0.0000	120	177.13	125	0.7057	carpenterjchemphys67	carpenterjchemphys67	CC(C)C1=CC=CC=C1	5A
isopropylbenzene	C9H12	2	0	6	1	0	0	0	4	0	0	0.0000	120	177.13	124.8	0.7046	wangjchemphys06	wangjchemphys06	CC(C)C1=CC=CC=C1	5A
isopropylbenzene	C9H12	2	0	6	1	0	0	0	4	0	0	0.0000	120	177	129.5	0.7316	murthyjchemsocfaradaytrans93	murthyjchemsocfaradaytrans93	CC(C)C1=CC=CC=C1	5A
isopropylbenzene	C9H12	2	0	6	1	0	0	0	4	0	0	0.0000	120	177.13	125	0.7057	wangjphyschemb07	wangjphyschemb07	CC(C)C1=CC=CC=C1	5A
isopropylbenzene	C9H12	2	0	6	1	0	0	0	4	0	0	0.0000	120	177	125	0.7062	naitojphychem93	naitojphychem93	CC(C)C1=CC=CC=C1	5A
diisobutylphthalat	C16H22O4	4	2	6	4	0	2	1	6	0	0	0.1875	262	209.15	191.1	0.9137	wangjphyschemb07	wangjphyschemb07	CC(C)COC(=O)C1=CC=CC=C1C(=O)OCC(C)C	
diisobutylphthalat	C16H22O4	4	2	6	4	0	2	1	6	0	0	0.1875	262	209.15	188	0.8989	carpenterjchemphys67	carpenterjchemphys67	CC(C)COC(=O)C1=CC=CC=C1C(=O)OCC(C)C	
1,4-pentanediol	C5H12O2	1	3	1	0	2	0	0	0	0	0	0.4000	104	253	175.8	0.6949	joback	wangjphyschemb07	CC(CCCO)O	5A, 5B, 6
1,2-pentanediol	C5H12O2	1	3	1	0	2	0	0	0	0	0	0.4000	104	253	181	0.7154	joback	wangjphyschemb07	CCCC(CO)O	5A, 5B
2,4-pentanediol	C5H12O2	2	1	2	0	2	0	0	0	0	0	0.4000	104	318.15	205.9	0.6472	mellan62	wangjphyschemb07	CC(CC(C)O)O	
1,5-hexanediol	C6H14O2	1	4	1	0	2	0	0	0	0	0	0.3333	118	264	179.4	0.6795	joback	wangjphyschemb07	CC(CCCCO)O	
iso-eugenol	C10H12O2	2	0	5	3	1	1	0	5	0	0	0.2000	164	294.15	220.1	0.7483	gestis2	wangjphyschemb07	CC=CC1=CC(=C(C=C1)O)OC	
ethanol	C2H6O	1	1	0	0	1	0	0	0	0	0	0.5000	46	159	94	0.5912	wangjchemphys06	wangjchemphys06	OCC	
m-cresol	C7H8O	1	0	4	1	1	0	0	4	0	0	0.1667	94	285.39	198.6	0.6959	wangjchemphys06	wangjchemphys06	CC1=CC(=CC=C1)O	
2-methyltetrahydrofuran	C5H10O	1	3	1	0	0	1	0	1	0	0	0.2000	86	137	91	0.6642	wangjchemphys06	wangjchemphys06	CC1CCCO1	5A, 6
menthol	C10H20O	3	3	4	0	1	0	0	1	0	0	0.1000	156	389	306	0.7866	tiersthermochimact93	tiersthermochimact93	CC1CCC(C(C1)O)C(C)C	
n-butylbenzene	C10H14	1	3	5	1	0	0	0	4	0	0	0.0000	134	185.3	125	0.6746	carpenterjchemphys67	carpenterjchemphys67	CCCCC1=CC=CC=C1	5A
n-butylbenzene	C10H14	1	3	5	1	0	0	0	4	0	0	0.0000	134	185	124	0.6703	naitojphychem93	naitojphychem93	CCCCC1=CC=CC=C1	5A
cyclohexanol	C6H12O	0	5	1	0	1	0	0	1	0	0	0.1667	100	173	151	0.8728	murthyjchemsocfaradaytrans93	murthyjchemsocfaradaytrans93	C1CCC(CC1)O	
cyclohexanol	C6H12O	0	5	1	0	1	0	0	1	0	0	0.1667	100	173	150	0.8671	naitojphychem93	naitojphychem93	C1CCC(CC1)O	
cycloheptanol	C7H14O	0	6	1	0	1	0	0	1	0	0	0.1429	114	280	140	0.5000	naitojphychem93	naitojphychem93	C1CCCC(CC1)O	

Name	Formula	#CH3	#CH2	#CH	#C	#OH	#C-O-C	#O=C	DBA	#N	#Hal	O:C	M / g/mol	Tm / K	Tg / K	Tg/Tm	Reference(Tm)	Reference(Tg)	SMILES	Figure
cycloheptane	C7H14	0	7	0	0	0	0	0	1	0	0	0.0000	98	265	83	0.3132	naitojphychem93	naitojphychem93	C1CCCCCC1	
2,3-dimethylbutane	C6H14	4	0	2	0	0	0	0	0	0	0	0.0000	86	145	76	0.5241	naitojphychem93	naitojphychem93	CC(C)C(C)C	
1-phenyl-1-propanol	C9H12O	1	1	6	1	1	0	0	4	0	0	0.1111	136	263.43	198.2	0.7524	joback	murthyjphyschem96	CCC(C1=CC=CC=C1)O	
1-phenyl-1-propanol	C9H12O	1	1	6	1	1	0	0	4	0	0	0.1111	136	263.43	193.6	0.7349	joback	johariannnyacadsci76	CCC(C1=CC=CC=C1)O	
1,2-butanediol	C4H10O2	1	2	1	0	2	0	0	0	0	0	0.5000	90	241	178.9	0.7423	joback	mariajthermanalcalorim10	CCC(CO)O	
chenodeoxycholic acid	C24H40O4	3	10	8	3	3	0	1	5	0	0	0.1667	392	436	371	0.8509	fukuokachempharmbull91	fukuokachempharmbull91	CC(CCC(=O)O)C1CCC2C1(CCC3C2C(CC4C3(CCC(C4)O)C)O)C	
chenodeoxycholic acid	C24H40O4	3	10	8	3	3	0	1	5	0	0	0.1667	392	436	371	0.8509	kercthermochimacta95	kercthermochimacta95	CC(CCC(=O)O)C1CCC2C1(CCC3C2C(CC4C3(CCC(C4)O)C)O)C	
cis-2-heptene	C7H14	2	3	2	0	0	0	0	1	0	0	0.0000	98	164.15	93	0.5666	gestis3	carpenterjchemphys67	CCCCC=CC	
2-methyl-1-heptanol	C8H18O	2	5	1	0	1	0	0	0	0	0	0.1250	130	161.15	158	0.9805	koleskepolymengsci79	koleskepolymengsci79	CCCCCC(C)CO	
2-methyl-1-pentanol	C6H14O	2	3	1	0	1	0	0	0	0	0	0.1667	102	203	143	0.7044	joback	koleskepolymengsci79	CCCC(C)CO	
2,2-dimethyl-1-butanol	C6H14O	3	2	0	1	1	0	0	0	0	0	0.1667	102	258.15	158	0.6120	koleskepolymengsci79	koleskepolymengsci79	CCC(C)(C)CO	5A
2,2-dimethyl-1-pentanol	C7H16O	3	3	0	1	1	0	0	0	0	0	0.1429	116	243	166	0.6831	yawshandbook	koleskepolymengsci79	CCCC(C)(C)CO	
2,2-dimethyl-1-hexanol	C8H18O	3	4	0	1	1	0	0	0	0	0	0.1250	130	243	173	0.7119	joback	koleskepolymengsci79	CCCCC(C)(C)CO	
2-hexanol	C6H14O	2	3	1	0	1	0	0	0	0	0	0.1667	102	253.15	148	0.5846	koleskepolymengsci79	koleskepolymengsci79	CCCCC(C)O	
3-hexanol	C6H14O	2	3	1	0	1	0	0	0	0	0	0.1667	102	279	153	0.5484	gestis4	koleskepolymengsci79	CCCC(CC)O	
3-heptanol	C7H16O	2	4	1	0	1	0	0	0	0	0	0.1429	116	203.15	158	0.7778	gestis5	koleskepolymengsci79	CCCCC(CC)O	
2-heptanol	C7H16O	2	4	1	0	1	0	0	0	0	0	0.1429	116	214	155	0.7243	joback	koleskepolymengsci79	CCCCCC(C)O	
4-nonanol	C9H20O	2	6	1	0	1	0	0	0	0	0	0.1111	144	237	175	0.7384	joback	koleskepolymengsci79	CCCCCC(CCC)O	
3-methyl-4-nonanol	C10H22O	3	5	2	0	1	0	0	0	0	0	0.1000	158	265.7	186	0.7000	joback	koleskepolymengsci79	CCCCCC(C(C)CC)O	
2-methyl-2-pentanol	C6H14O	3	2	0	1	1	0	0	0	0	0	0.1667	102	170.15	163	0.9580	koleskepolymengsci79	koleskepolymengsci79	CCCC(C)(C)O	5A
2-methyl-2-hexanol	C7H16O	3	3	0	1	1	0	0	0	0	0	0.1429	116	243	173	0.7119	yawshandbook	koleskepolymengsci79	CCCCC(C)(C)O	
4-methy-4-octanol	C9H20O	3	5	0	1	1	0	0	0	0	0	0.1111	144	254.43	178	0.6996	joback	koleskepolymengsci79	CCCCC(C)(CCC)O	
4-methyl-4-nonanol	C10H22O	3	6	0	1	1	0	0	0	0	0	0.1000	158	272	185	0.6801	yawshandbook	koleskepolymengsci79	CCCCCC(C)(CCC)O	5A
4-methyl-undecane	C12H26	3	8	1	0	0	0	0	0	0	0	0.0000	170	206.5	135	0.6538	petrov1959	koleskepolymengsci79	CCCCCCCC(C)CCC	
n-butanol	C4H10O	1	3	0	0	1	0	0	0	0	0	0.2500	74	184.55	113.38	0.6144	murthyjchemsocfaradaytrans93	murthyjchemsocfaradaytrans93	CCCCO	5B
n-butanol	C4H10O	1	3	0	0	1	0	0	0	0	0	0.2500	74	184.55	111.55	0.6044	lesikarjchemphys75	lesikarjchemphys75	CCCCO	5B
n-butanol	C4H10O	1	3	0	0	1	0	0	0	0	0	0.2500	74	184.55	114	0.6177	johariannnyacadsci76	johariannnyacadsci76	CCCCO	5B
n-butanol	C4H10O	1	3	0	0	1	0	0	0	0	0	0.2500	74	183	119	0.6503	naitojphychem93	naitojphychem93	CCCCO	5B
n-butanol	C4H10O	1	3	0	0	1	0	0	0	0	0	0.2500	74	183.5	113.6	0.6191	murthyjchemsocfaradaytrans93	murthyjchemsocfaradaytrans93	CCCCO	5B
xylose	C5H10O5	0	1	3	1	5	0	1	1	0	0	1.2000	166	426	283	0.6643	kercthermochimacta95	kercthermochimacta95	C(C(C(C(C=O)O)O)O)O	
fructose	C6H12O6	0	2	3	1	5	1	0	1	0	0	1.0000	180	373	286	0.7668	kercthermochimacta95	kercthermochimacta95	C1C(C(C(C(O1)(CO)O)O)O)O	
fructose	C6H12O6	0	2	3	1	5	1	0	1	0	0	1.0000	180	376.15	286	0.7603	wangjchemphys06	wangjchemphys06	C1C(C(C(C(O1)(CO)O)O)O)O	
tartaric acid	C4H6O6	0	0	2	2	4	0	2	2	0	0	1.5000	150	430	289	0.6721	kercthermochimacta95	kercthermochimacta95	O=C(O)C(O)C(O)C(=O)O	
ethanol	C2H6O	1	1	0	0	1	0	0	0	0	0	0.5000	46	159.01	97.25	0.6116	lesikarjchemphys75	lesikarjchemphys75	OCC	
ethanol	C2H6O	1	1	0	0	1	0	0	0	0	0	0.5000	46	161	93	0.5776	beamanjpolysci52	beamanjpolysci52	OCC	
ethanol	C2H6O	1	1	0	0	1	0	0	0	0	0	0.5000	46	159.01	96	0.6037	carpenterjchemphys67	carpenterjchemphys67	OCC	
ethanol	C2H6O	1	1	0	0	1	0	0	0	0	0	0.5000	46	159.01	97.05	0.6103	lesikar_jsolchem_77	lesikar_jsolchem_77	OCC	
n-butanol	C4H10O	1	3	0	0	1	0	0	0	0	0	0.2500	74	184.55	111.65	0.6050	lesikar_jsolchem_77	lesikar_jsolchem_77	CCCCO	5B
n-hexanol	C6H14O	1	5	0	0	1	0	0	0	0	0	0.1667	102	225.75	129.85	0.5752	lesikarjchemphys75	lesikarjchemphys75	CCCCCCO	
n-hexanol	C6H14O	1	5	0	0	1	0	0	0	0	0	0.1667	102	225.75	129.85	0.5752	lesikar_jsolchem_77	lesikar_jsolchem_77	CCCCCCO	
n-pentanol	C5H12O	1	4	0	0	1	0	0	0	0	0	0.2000	88	195.55	120.95	0.6185	lesikar_jsolchem_77	lesikar_jsolchem_77	CCCCCO	
n-pentanol	C5H12O	1	4	0	0	1	0	0	0	0	0	0.2000	88	195.55	120.75	0.6175	lesikarjchemphys75	lesikarjchemphys75	CCCCCO	
n-heptanol	C7H16O	1	6	0	0	1	0	0	0	0	0	0.1429	116	240	141.15	0.5881	lesikar_jsolchem_77	lesikar_jsolchem_77	CCCCCCCO	5B
n-octanol	C8H18O	1	7	0	0	1	0	0	0	0	0	0.1250	130	258.35	149.85	0.5800	lesikar_jsolchem_77	lesikar_jsolchem_77	CCCCCCCCO	6
3-methyl-1-butanol	C5H12O	2	2	1	0	1	0	0	0	0	0	0.2000	88	155.95	123.2	0.7900	murthyjchemsocfaradaytrans93	murthyjchemsocfaradaytrans93	CC(C)CCO	
2-methyl-1-butanol	C5H12O	2	2	1	0	1	0	0	0	0	0	0.2000	88	195	131	0.6718	koleskepolymengsci79	koleskepolymengsci79	CCC(C)CO	
1,2,4-butanetriol	C4H10O3	0	3	1	0	3	0	0	0	0	0	0.7500	106	253.15	200.7	0.7928	gestis6	nakanishi2011	C(CO)C(CO)O	
benzene	C6H6	0	0	6	0	0	0	0	4	0	0	0.0000	78	279.15	118	0.4227	gestis7	xia2006	C1=CC=CC=C1	
2,4,6-trimethylheptane	C10H22	5	2	3	0	0	0	0	0	0	0	0.0000	142	219.16	126	0.5749	wangjchemphys08	wangjchemphys08	CC(C)CC(C)CC(C)C	5B
santonin	C15H18O3	3	2	5	5	0	1	2	7	0	0	0.2000	246	434	290	0.6682	kercthermochimacta95	kercthermochimacta95	CC1C2CCC3(C=CC(=O)C(=C3C2OC1=O)C)C	5A
maltotetraose	C24H42O21	0	4	20	0	14	7	0	4	0	0	0.8750	666	484	401	0.8285	trccanada	imamura2006	C(C1C(C(C(C(O1)OC2C(OC(C(C2O)O)OC3C(OC(C(C3O)O)OC4C(OC(C(C4O)O)O)CO)CO)CO)O)O)O)O	
maltotetraose	C24H42O21	0	4	20	0	14	7	0	4	0	0	0.8750	666	484	421	0.8698	trccanada	orford89	C(C1C(C(C(C(O1)OC2C(OC(C(C2O)O)OC3C(OC(C(C3O)O)OC4C(OC(C(C4O)O)O)CO)CO)CO)O)O)O)O	
malic acid	C4H6O5	0	1	1	2	3	0	2	2	0	0	1.2500	134	405	252	0.6222	crchandbook	maltini97	C(C(C(=O)O)O)C(=O)O	
malic acid	C4H6O5	0	1	1	2	3	0	2	2	0	0	1.2500	134	405	258	0.6370	crchandbook	curtin2013	C(C(C(=O)O)O)C(=O)O	
pinonic acid	C10H16O3	3	2	2	3	1	0	2	3	0	0	0.3000	184	376.68	265	0.7035	kolodziejczyk19	zhang2019	CC(=O)C1CC(C1(C)C)CC(=O)O	
2-methylbutane-1,2,3,4-tetraol	C5H12O4	1	2	1	1	4	0	0	0	0	0	0.8000	136	363	238	0.6556	lessmeier18	lessmeier18	CC(CO)(C(CO)O)O	6
2-methylbutane-1,2,3,4-tetraol	C5H12O4	1	2	1	1	4	0	0	0	0	0	0.8000	136	363	245	0.6749	lessmeier18	lessmeier18	CC(CO)(C(CO)O)O	6
1,2-isopooh	C5H10O3	1	2	1	1	2	1	0	1	0	0	0.6000	118	220	142	0.6455	engineeringtoolbox	zhang2019	C=C(C)C(CO)OO	
trans-beta-iepox	C5H10O3	1	2	1	1	2	1	0	1	0	0	0.6000	118	220	167.14	0.7597	engineeringtoolbox	zhang2019	CC1(CO)OC1CO	
pinic acid	C9H14O4	2	2	2	3	2	0	2	3	0	0	0.4444	186	364.32	268.1	0.7359	kolodziejczyk19	koop2011	CC1(C(CC1C(=O)O)CC(=O)O)C	
2,4-dimethyl-3-pentanol	C7H16O	4	0	3	0	1	0	0	0	0	0	0.1429	116	203.15	193	0.9500	bradleydataset	johari71	CC(C)C(C(C)C)O	
erythritol	C4H10O4	0	2	2	0	4	0	0	0	0	0	1.0000	122	394	228.3	0.5794	bradleydataset	talja2001	C(C(C(CO)O)O)O	5A, 5B
erythritol	C4H10O4	0	2	2	0	4	0	0	0	0	0	1.0000	122	394	231	0.5863	bradleydataset	o'donnell2012	C(C(C(CO)O)O)O	5A, 5B
2-ethyl-1,3-hexanediol	C816O2	2	4	2	0	2	0	0	0	0	0	0.2500	146	272	208.9	0.7680	joback	wangjchemphys08	CCCC(C(CC)CO)O	
1,2,7-heptanetriol	C7H16O3	0	6	1	0	3	0	0	0	0	0	0.4286	148	336	200.3	0.5961	joback	nakanishi2011	C(CCC(CO)O)CCO	5B
1,2,6-hexanetriol	C6H14O3	0	5	1	0	3	0	0	0	0	0	0.5000	134	300	201.9	0.6730	chembook1	nakanishi2011	C(CCO)CC(CO)O	
1,2,6-hexanetriol	C6H14O3	0	5	1	0	3	0	0	0	0	0	0.5000	134	300	206.4	0.6880	chembook1	dorfmueller79	C(CCO)CC(CO)O	
mannitol	C6H14O6	0	2	4	0	6	0	0	0	0	0	1.0000	182	437	282	0.6453	crchandbook	angell82	C(C(C(C(C(CO)O)O)O)O)O	
mannitol	C6H14O6	0	2	4	0	6	0	0	0	0	0	1.0000	182	437	283.9	0.6497	crchandbook	yu98	C(C(C(C(C(CO)O)O)O)O)O	
2-methyl-1,3-propanediol	C4H10O2	1	2	1	0	2	0	0	0	0	0	0.5000	90	242	167	0.6901	joback	jabrane98	CC(CO)CO	
3-methyl-2-butanol	C5H12O	3	0	2	0	1	0	0	0	0	0	0.2000	88	222	154	0.6937	yawshandbook	faucher66	CC(C)C(C)O	
3-methyl-1,3,5-pentanetriol	C6H14O3	1	4	0	1	3	0	0	0	0	0	0.5000	134	342	205.7	0.6015	joback	nakanishi2011	CC(CCO)(CCO)O	5B
1,3-propanediol	C3H8O2	0	3	0	0	2	0	0	0	0	0	0.6667	76	246.5	144.6	0.5866	bradleydataset	takeda98	C(CO)CO	
1,3-propanediol	C3H8O2	0	3	0	0	2	0	0	0	0	0	0.6667	76	246.5	150.4	0.6101	bradleydataset	jabrane98	C(CO)CO	
arabitol	C5H12O5	0	2	3	0	5	0	0	0	0	0	1.0000	152	375	261	0.6960	bradleydataset	carpentier2003	C(C(C(C(CO)O)O)O)O	5A, 6
ribitol	C5H12O5	0	2	3	0	5	0	0	0	0	0	1.0000	152	369	252	0.6829	crchandbook	carpentier2003	C(C(C(C(CO)O)O)O)O	5A, 6
threitol	C4H10O4	0	2	2	0	4	0	0	0	0	0	1.0000	122	363	226.3	0.6234	yawshandbook	nakanishi2011	C(C(C(CO)O)O)O	5A, 5B
xylitol	C5H12O5	0	2	3	0	2	0	0	0	0	0	0.4000	104	367	244	0.6649	bradleydataset	roos93	C(C(C(C(CO)O)O)O)O	5A, 6
xylitol	C5H12O5	0	2	3	0	2	0	0	0	0	0	0.4000	104	367	247.8	0.6752	bradleydataset	nakanishi2011	C(C(C(C(CO)O)O)O)O	5A, 6
xylitol	C5H12O5	0	2	3	0	2	0	0	0	0	0	0.4000	104	367	248.9	0.6782	bradleydataset	talja2001	C(C(C(C(CO)O)O)O)O	5A, 6
xylitol	C5H12O5	0	2	3	0	2	0	0	0	0	0	0.4000	104	367	250	0.6812	bradleydataset	carpentier2003	C(C(C(C(CO)O)O)O)O	5A, 6
xylitol	C5H12O5	0	2	3	0	2	0	0	0	0	0	0.4000	104	367	254	0.6921	bradleydataset	orford90	C(C(C(C(CO)O)O)O)O	5A, 6
altrose	C6H12O6	0	1	4	1	5	0	1	1	0	0	1.0000	180	377	283.5	0.7520	crchandbook	slade94	C(C1C(C(C(C(O1)O)O)O)O)O	
arabinose	C5H10O5	0	1	3	1	4	0	1	1	0	0	1.0000	150	433	271	0.6259	yawshandbook	roos93	C(C(C(C(C=O)O)O)O)O	
arabinose	C5H10O5	0	1	3	1	4	0	1	1	0	0	1.0000	150	433	277	0.6397	yawshandbook	orford90	C(C(C(C(C=O)O)O)O)O	
cellobiose	C12H22O11	0	2	10	0	8	3	0	2	0	0	0.9167	342	512.15	372	0.7263	yawshandbook	pinto2010	C(C1C(C(C(C(O1)OC2C(OC(C(C2O)O)O)CO)O)O)O)O	6
cellobiose	C12H22O11	0	2	10	0	8	3	0	2	0	0	0.9167	342	512.15	381.3	0.7445	yawshandbook	miller2000	C(C1C(C(C(C(O1)OC2C(OC(C(C2O)O)O)CO)O)O)O)O	6
fructose	C6H12O6	0	2	3	1	5	1	0	1	0	0	1.0000	180	373	274	0.7346	roos93	wungtanagorn2001	C1C(C(C(C(O1)(CO)O)O)O)O	
fructose	C6H12O6	0	2	3	1	5	1	0	1	0	0	1.0000	180	373	278	0.7453	roos93	roos93	C1C(C(C(C(O1)(CO)O)O)O)O	
fructose	C6H12O6	0	2	3	1	5	1	0	1	0	0	1.0000	180	373	280	0.7507	roos93	orford90	C1C(C(C(C(O1)(CO)O)O)O)O	
galactose	C6H12O6	0	1	4	1	5	0	1	1	0	0	1.0000	180	439	303	0.6902	bradleydataset	roos93	C(C1C(C(C(C(O1)O)O)O)O)O	

Name	Formula	#CH3	#CH2	#CH	#C	#OH	#C-O-C	#O=C	DBA	#N	#Hal	O:C	M / g/mol	Tm / K	Tg / K	Tg/Tm	Reference(Tm)	Reference(Tg)	SMILES	Figure
galactose	C6H12O6	0	1	4	1	5	0	1	1	0	0	1.0000	180	439	305	0.6948	bradleydataset	orford90	C(C1C(C(C(C(O1)O)O)O)O)O	
gentiobiose	C12H22O11	0	2	10	0	8	3	0	2	0	0	0.9167	342	482	359	0.7448	yawshandbook	pinto2010	C(C1C(C(C(C(O1)OCC2C(C(C(C(O2)O)O)O)O)O)O)O)O	
gentiobiose	C12H22O11	0	2	10	0	8	3	0	2	0	0	0.9167	342	482	374	0.7759	yawshandbook	miller2000	C(C1C(C(C(C(O1)OCC2C(C(C(C(O2)O)O)O)O)O)O)O)O	
isomalt	C12H24O11	0	3	9	0	9	2	0	1	0	0	0.9167	344	420.75	307	0.7296	römpp	o'donnell2012	C(C1C(C(C(C(O1)OCC(C(C(C(CO)O)O)O)O)O)O)O)O	
isomaltose	C12H22O11	0	2	10	0	8	3	0	2	0	0	0.9167	342	498.15	351	0.7046	römpp	orford90	C(C1C(C(C(C(O1)OCC2C(C(C(C(O2)O)O)O)O)O)O)O)O	
isomaltose	C12H22O11	0	2	10	0	8	3	0	2	0	0	0.9167	342	498.15	351	0.7046	römpp	shamblin99	C(C1C(C(C(C(O1)OCC2C(C(C(C(O2)O)O)O)O)O)O)O)O	
isomaltulose	C12H22O11	0	3	8	1	8	3	0	2	0	0	0.9167	342	396.15	342.1	0.8636	tiefenbacher17	miller2000	C(C1C(C(C(C(O1)OCC(C(C(C(=O)CO)O)O)O)O)O)O)O	
kojibiose	C12H22O11	0	2	10	0	8	2	1	2	0	0	0.9167	342	461	359	0.7787	yamauchi61	imamura2006	C(C1C(C(C(C(O1)OC(C=O)C(C(C(CO)O)O)O)O)O)O)O	
lactose	C12H22O11	0	2	10	0	8	3	0	2	0	0	0.9167	342	496	374	0.7540	crchandbook	roos93	C(C1C(C(C(C(O1)OC(C(CO)O)C(C(C=O)O)O)O)O)O)O	6
lactose	C12H22O11	0	2	10	0	8	3	0	2	0	0	0.9167	342	496	381	0.7681	crchandbook	saleki-gerhardt94	C(C1C(C(C(C(O1)OC(C(CO)O)C(C(C=O)O)O)O)O)O)O	6
lactose	C12H22O11	0	2	10	0	8	3	0	2	0	0	0.9167	342	496	385.3	0.7768	crchandbook	miller2000	C(C1C(C(C(C(O1)OC(C(CO)O)C(C(C=O)O)O)O)O)O)O	6
lactose	C12H22O11	0	2	10	0	8	3	0	2	0	0	0.9167	342	496	387	0.7802	crchandbook	taylor98	C(C1C(C(C(C(O1)OC(C(CO)O)C(C(C=O)O)O)O)O)O)O	6
lactose	C12H22O11	0	2	10	0	8	3	0	2	0	0	0.9167	342	496	387	0.7802	crchandbook	elamin95	C(C1C(C(C(C(O1)OC(C(CO)O)C(C(C=O)O)O)O)O)O)O	6
lactulose	C12H22O11	0	3	8	1	8	3	0	2	0	0	0.9167	342	442	367.4	0.8312	crchandbook	miller2000	C(C1C(C(C(C(O1)OC2C(OC(C2O)(CO)O)CO)O)O)O)O	
leucrose	C12H22O11	0	3	8	1	8	2	1	2	0	0	0.9167	342	435	362.7	0.8338	stodola56	miller2000	C(C1C(C(C(C(O1)OC2C(OC(C2O)(CO)O)CO)O)O)O)O	
levoglucosan	C6H10O5	0	1	5	0	3	2	0	2	0	0	0.8333	162	456	247.8	0.5434	bradleydataset	tombari2015	C1C2C(C(C(C(O1)O2)O)O)O	
levoglucosan	C6H10O5	0	1	5	0	3	2	0	2	0	0	0.8333	162	456	249	0.5461	bradleydataset	lienhard2012	C1C2C(C(C(C(O1)O2)O)O)O	
lyxose	C5H10O5	0	1	3	1	4	0	1	1	0	0	1.0000	150	383.15	277.1	0.7232	bradleydataset	singh2011	C(C(C(C(C=O)O)O)O)O	
maltitol	C12H24O11	0	3	9	0	9	2	0	1	0	0	0.9167	344	423.65	312	0.7365	roos93	roos93	C(C1C(C(C(C(O1)OC(C(CO)O)C(C(CO)O)O)O)O)O)O	6
maltoheptaose	C42H72O36	0	7	35	0	23	13	0	7	0	0	0.8571	1152	435	423	0.9724	lei2022	imamura2006	C(C1C(C(C(C(O1)OC2C(OC(C(C2O)O)OC3C(OC(C(C3O)O)OC4C(OC(C(C4O)O)OC5C(OC(C(C5O)O)OC6C(OC(C(C6O)O)OC7C(OC(C(C7O)O)O)CO)CO)CO)CO)CO)CO)O)O)O)O	
maltohexaose	C36H62O31	0	6	30	0	20	11	0	6	0	0	0.8611	990	479.15	418	0.8724	chembook2	imamura2006	C(C1C(C(C(C(O1)OC2C(OC(C(C2O)O)OC3C(OC(C(C3O)O)OC4C(OC(C(C4O)O)OC5C(OC(C(C5O)O)OC6C(OC(C(C6O)O)O)CO)CO)CO)CO)CO)O)O)O)O	
maltohexaose	C36H62O31	0	6	30	0	20	11	0	6	0	0	0.8611	990	479.15	448	0.9350	chembook2	orford90	C(C1C(C(C(C(O1)OC2C(OC(C(C2O)O)OC3C(OC(C(C3O)O)OC4C(OC(C(C4O)O)OC5C(OC(C(C5O)O)OC6C(OC(C(C6O)O)O)CO)CO)CO)CO)CO)O)O)O)O	
maltopentaose	C30H52O26	0	5	25	0	17	9	0	5	0	0	0.8667	828	441.15	410	0.9294	carboExpert	imamura2006	C(C1C(C(C(C(O1)OC2C(OC(C(C2O)O)OC3C(OC(C(C3O)O)OC4C(OC(C(C4O)O)OC5C(OC(C(C5O)O)O)CO)CO)CO)CO)O)O)O)O	
maltopentaose	C30H52O26	0	5	25	0	17	9	0	5	0	0	0.8667	828	441.15	443	1.0042	carboExpert	orford89	C(C1C(C(C(C(O1)OC2C(OC(C(C2O)O)OC3C(OC(C(C3O)O)OC4C(OC(C(C4O)O)OC5C(OC(C(C5O)O)O)CO)CO)CO)CO)O)O)O)O	
maltose	C12H22O11	0	2	10	0	8	3	0	2	0	0	0.9167	342	436	360	0.8257	crchandbook	roos93	C(C1C(C(C(C(O1)OC2C(OC(C(C2O)O)O)CO)O)O)O)O	
maltose	C12H22O11	0	2	10	0	8	3	0	2	0	0	0.9167	342	436	363	0.8326	crchandbook	imamura2006	C(C1C(C(C(C(O1)OC2C(OC(C(C2O)O)O)CO)O)O)O)O	
maltose	C12H22O11	0	2	10	0	8	3	0	2	0	0	0.9167	342	436	363	0.8326	crchandbook	kawai2005	C(C1C(C(C(C(O1)OC2C(OC(C(C2O)O)O)CO)O)O)O)O	
maltose	C12H22O11	0	2	10	0	8	3	0	2	0	0	0.9167	342	436	364	0.8349	crchandbook	orford89	C(C1C(C(C(C(O1)OC2C(OC(C(C2O)O)O)CO)O)O)O)O	
maltose	C12H22O11	0	2	10	0	8	3	0	2	0	0	0.9167	342	436	368	0.8440	crchandbook	orford90	C(C1C(C(C(C(O1)OC2C(OC(C(C2O)O)O)CO)O)O)O)O	
maltose	C12H22O11	0	2	10	0	8	3	0	2	0	0	0.9167	342	436	373	0.8555	crchandbook	taylor98	C(C1C(C(C(C(O1)OC2C(OC(C(C2O)O)O)CO)O)O)O)O	
maltose	C12H22O11	0	2	10	0	8	3	0	2	0	0	0.9167	342	436	373.8	0.8573	crchandbook	miller2000	C(C1C(C(C(C(O1)OC2C(OC(C(C2O)O)O)CO)O)O)O)O	
maltotriose	C18H32O16	0	3	15	0	11	5	0	3	0	0	0.8889	504	407	383	0.9410	yawshandbook	imamura2006	C(C1C(C(C(C(O1)OC2C(OC(C(C2O)O)OC3C(OC(C(C3O)O)O)CO)CO)O)O)O)O	
maltotriose	C18H32O16	0	3	15	0	11	5	0	3	0	0	0.8889	504	407	407	1.0000	yawshandbook	orford90	C(C1C(C(C(C(O1)OC2C(OC(C(C2O)O)OC3C(OC(C(C3O)O)O)CO)CO)O)O)O)O	
mannose	C6H12O6	0	1	4	1	5	0	1	1	0	0	1.0000	180	405.15	298	0.7355	yawshandbook	roos93	C(C1C(C(C(C(O1)O)O)O)O)O	
mannose	C6H12O6	0	1	4	1	5	0	1	1	0	0	1.0000	180	405.15	309	0.7627	yawshandbook	orford90	C(C1C(C(C(C(O1)O)O)O)O)O	
mannobiose	C12H22O11	0	2	10	0	8	3	0	2	0	0	0.9167	342	476.65	363	0.7616	hmdb	slade94	C(C1C(C(C(C(O1)OC2C(OC(C(C2O)O)O)CO)O)O)O)O	
melibiose	C12H22O11	0	2	10	0	8	3	0	2	0	0	0.9167	342	457	358	0.7834	römpp	roos93	C(C1C(C(C(C(O1)OCC2C(C(C(C(O2)O)O)O)O)O)O)O)O	
melibiose	C12H22O11	0	2	10	0	8	3	0	2	0	0	0.9167	342	457	368	0.8053	römpp	orford90	C(C1C(C(C(C(O1)OCC2C(C(C(C(O2)O)O)O)O)O)O)O)O	
melibiose	C12H22O11	0	2	10	0	8	3	0	2	0	0	0.9167	342	457	374.5	0.8195	römpp	miller2000	C(C1C(C(C(C(O1)OCC2C(C(C(C(O2)O)O)O)O)O)O)O)O	
nigerose	C12H22O11	0	2	10	0	8	3	0	2	0	0	0.9167	342	429	362	0.8438	römpp	imamura2006	C(C1C(C(C(C(O1)O)O)OC2C(C(C(C(O2)CO)O)O)O)O)O	
nystose	C24H42O21	0	7	14	3	14	7	0	4	0	0	0.8750	666	405	350	0.8642	römpp	slade94	C(C1C(C(C(C(O1)OC2(C(C(C(O2)CO)O)O)COC3(C(C(C(O3)CO)O)O)COC4(C(C(C(O4)CO)O)O)CO)O)O)O)O	
raffinose	C18H32O16	0	4	13	1	11	5	0	3	0	0	0.8889	504	416	375	0.9014	lei2022	saleki-gerhardt94	C(C1C(C(C(C(O1)OCC2C(C(C(C(O2)OC3(C(C(C(O3)CO)O)O)CO)O)O)O)O)O)O)O	
raffinose	C18H32O16	0	4	13	1	11	5	0	3	0	0	0.8889	504	416	376.4	0.9048	lei2022	kajiwara97	C(C1C(C(C(C(O1)OCC2C(C(C(C(O2)OC3(C(C(C(O3)CO)O)O)CO)O)O)O)O)O)O)O	
raffinose	C18H32O16	0	4	13	1	11	5	0	3	0	0	0.8889	504	416	379	0.9111	lei2022	buitink2000	C(C1C(C(C(C(O1)OCC2C(C(C(C(O2)OC3(C(C(C(O3)CO)O)O)CO)O)O)O)O)O)O)O	
raffinose	C18H32O16	0	4	13	1	11	5	0	3	0	0	0.8889	504	416	382	0.9183	lei2022	moura_ramos2005	C(C1C(C(C(C(O1)OCC2C(C(C(C(O2)OC3(C(C(C(O3)CO)O)O)CO)O)O)O)O)O)O)O	
raffinose	C18H32O16	0	4	13	1	11	5	0	3	0	0	0.8889	504	416	385.6	0.9269	lei2022	miller2000	C(C1C(C(C(C(O1)OCC2C(C(C(C(O2)OC3(C(C(C(O3)CO)O)O)CO)O)O)O)O)O)O)O	
raffinose	C18H32O16	0	4	13	1	11	5	0	3	0	0	0.8889	504	416	387	0.9303	lei2022	taylor98	C(C1C(C(C(C(O1)OCC2C(C(C(C(O2)OC3(C(C(C(O3)CO)O)O)CO)O)O)O)O)O)O)O	
raffinose	C18H32O16	0	4	13	1	11	5	0	3	0	0	0.8889	504	416	387	0.9303	lei2022	lienhard2012	C(C1C(C(C(C(O1)OCC2C(C(C(C(O2)OC3(C(C(C(O3)CO)O)O)CO)O)O)O)O)O)O)O	
rhamnose	C6H12O5	1	0	4	1	4	0	1	1	0	0	0.8333	164	355	266	0.7493	yawshandbook	roos93	CC1C(C(C(C(O1)O)O)O)O	
rhamnose	C6H12O5	1	0	4	1	4	0	1	1	0	0	0.8333	164	355	300	0.8451	yawshandbook	orford90	CC1C(C(C(C(O1)O)O)O)O	
fucose	C6H12O5	1	0	4	1	4	0	1	1	0	0	0.8333	164	413	299	0.7240	yawshandbook	roos93	CC1C(C(C(C(O1)O)O)O)O	
ribose	C5H10O5	0	1	3	1	4	0	1	1	0	0	1.0000	150	361	253	0.7008	yawshandbook	roos93	C1C(C(C(C(O1)O)O)O)O	
ribose	C5H10O5	0	1	3	1	4	0	1	1	0	0	1.0000	150	361	262	0.7258	yawshandbook	orford90	C1C(C(C(C(O1)O)O)O)O	
sorbose	C6H12O6	0	2	3	1	5	0	1	1	0	0	1.0000	180	436	292	0.6697	yawshandbook	roos93	C(C(C(C(C(=O)CO)O)O)O)O	6
stachyose	C24H42O21	0	5	18	1	14	7	0	4	0	0	0.8750	666	441	396	0.8980	römpp	buitink2000	C(C1C(C(C(C(O1)OCC2C(C(C(C(O2)OCC3C(C(C(C(O3)OC4(C(C(C(O4)CO)O)O)CO)O)O)O)O)O)O)O)O)O)O)O	
talose	C6H12O6	0	1	4	1	5	0	1	1	0	0	1.0000	180	407	284.7	0.6995	bradleydataset	slade94	C(C1C(C(C(C(O1)O)O)O)O)O	
trehalose	C12H22O11	0	2	10	0	8	3	0	2	0	0	0.9167	342	483	370	0.7660	crchandbook	imamura2006	C(C1C(C(C(C(O1)OC2C(C(C(C(O2)CO)O)O)O)O)O)O)O	
trehalose	C12H22O11	0	2	10	0	8	3	0	2	0	0	0.9167	342	483	373	0.7723	crchandbook	roos93	C(C1C(C(C(C(O1)OC2C(C(C(C(O2)CO)O)O)O)O)O)O)O	
trehalose	C12H22O11	0	2	10	0	8	3	0	2	0	0	0.9167	342	483	378	0.7826	crchandbook	shamblin99	C(C1C(C(C(C(O1)OC2C(C(C(C(O2)CO)O)O)O)O)O)O)O	
trehalose	C12H22O11	0	2	10	0	8	3	0	2	0	0	0.9167	342	483	380	0.7867	crchandbook	simperler2006	C(C1C(C(C(C(O1)OC2C(C(C(C(O2)CO)O)O)O)O)O)O)O	
trehalose	C12H22O11	0	2	10	0	8	3	0	2	0	0	0.9167	342	483	380	0.7867	crchandbook	buitink2000	C(C1C(C(C(C(O1)OC2C(C(C(C(O2)CO)O)O)O)O)O)O)O	
trehalose	C12H22O11	0	2	10	0	8	3	0	2	0	0	0.9167	342	483	386	0.7992	crchandbook	kawai2005	C(C1C(C(C(C(O1)OC2C(C(C(C(O2)CO)O)O)O)O)O)O)O	
trehalose	C12H22O11	0	2	10	0	8	3	0	2	0	0	0.9167	342	483	387	0.8012	crchandbook	crowe96	C(C1C(C(C(C(O1)OC2C(C(C(C(O2)CO)O)O)O)O)O)O)O	
trehalose	C12H22O11	0	2	10	0	8	3	0	2	0	0	0.9167	342	483	388	0.8033	crchandbook	miller97	C(C1C(C(C(C(O1)OC2C(C(C(C(O2)CO)O)O)O)O)O)O)O	
trehalose	C12H22O11	0	2	10	0	8	3	0	2	0	0	0.9167	342	483	388	0.8033	crchandbook	saleki-gerhardt94	C(C1C(C(C(C(O1)OC2C(C(C(C(O2)CO)O)O)O)O)O)O)O	
trehalose	C12H22O11	0	2	10	0	8	3	0	2	0	0	0.9167	342	483	390	0.8075	crchandbook	miller2000	C(C1C(C(C(C(O1)OC2C(C(C(C(O2)CO)O)O)O)O)O)O)O	
trehalose	C12H22O11	0	2	10	0	8	3	0	2	0	0	0.9167	342	483	391	0.8095	crchandbook	taylor98	C(C1C(C(C(C(O1)OC2C(C(C(C(O2)CO)O)O)O)O)O)O)O	
turanose	C12H22O11	0	3	8	1	8	2	1	2	0	0	0.9167	342	441	325	0.7370	crchandbook	slade94	C(C1C(C(C(C(O1)OC(C(C(CO)O)O)C(=O)CO)O)O)O)O	
turanose	C12H22O11	0	3	8	1	8	2	1	2	0	0	0.9167	342	441	361.4	0.8195	crchandbook	miller2000	C(C1C(C(C(C(O1)OC(C(C(CO)O)O)C(=O)CO)O)O)O)O	
xylose	C5H10O5	0	1	3	1	5	0	1	1	0	0	1.2000	166	422	279	0.6611	bradleydataset	roos93	C(C(C(C(C=O)O)O)O)O	
xylose	C5H10O5	0	1	3	1	5	0	1	1	0	0	1.2000	166	422	286	0.6777	bradleydataset	orford90	C(C(C(C(C=O)O)O)O)O	
1,2,4-butanetricarboxylic acid	C7H10O6	0	3	1	3	3	0	3	3	0	0	0.8571	190	391.65	269.6	0.6884	koop2011	koop2011	C(CC(=O)O)C(CC(=O)O)C(=O)O	5A
citric acid	C6H8O7	0	2	0	4	4	0	3	3	0	0	1.1667	192	426	279	0.6549	crchandbook	maltini97	C(C(=O)O)C(CC(=O)O)(C(=O)O)O	
citric acid	C6H8O7	0	2	0	4	4	0	3	3	0	0	1.1667	192	426	284	0.6667	crchandbook	lu97	C(C(=O)O)C(CC(=O)O)(C(=O)O)O	
citric acid	C6H8O7	0	2	0	4	4	0	3	3	0	0	1.1667	192	426	284.7	0.6683	crchandbook	hoppu2009	C(C(=O)O)C(CC(=O)O)(C(=O)O)O	
citric acid	C6H8O7	0	2	0	4	4	0	3	3	0	0	1.1667	192	426	284.85	0.6687	crchandbook	timko79	C(C(=O)O)C(CC(=O)O)(C(=O)O)O	
citric acid	C6H8O7	0	2	0	4	4	0	3	3	0	0	1.1667	192	426	285.5	0.6702	crchandbook	lienhard2012	C(C(=O)O)C(CC(=O)O)(C(=O)O)O	
lactic acid	C3H6O3	1	0	1	1	2	0	1	1	0	0	1.0000	90	299	213	0.7124	naitojphychem93	maltini97	CC(C(=O)O)O	
3-methyl-1,2,3-butanetricarboxylic acid	C8H12O6	2	1	1	4	3	0	3	3	0	0	0.7500	204	416.15	305	0.7329	koop2011	dette2014	CC(C)(C(CC(=O)O)C(=O)O)C(=O)O	
1,2,3-propanetricarboxylic acid	C6H8O6	0	2	1	3	3	0	3	3	0	0	1.0000	176	431.15	268.9	0.6237	koop2011	koop2011	C(C(CC(=O)O)C(=O)O)C(=O)O	
tartaric acid	C4H6O6	0	0	2	2	4	0	2	2	0	0	1.5000	150	430	284.1	0.6607	kercthermochimacta95	lienhard2012	O=C(O)C(O)C(O)C(=O)O	
tartaric acid	C4H6O6	0	0	2	2	4	0	2	2	0	0	1.5000	150	430	291	0.6767	maltini97	maltini97	O=C(O)C(O)C(O)C(=O)O	
cyclooctanol	C8H16O	0	7	1	0	1	0	0	1	0	0	0.1250	128	293	168	0.5734	bradleydataset	yamamuro99	C1CCCC(CCC1)O	
1,2-dimethylcyclohexane	C8H16	2	4	2	0	0	0	0	1	0	0	0.0000	112	201	97	0.4826	yawshandbook	alba90	CC1CCCCC1C	
2-methylbutane	C5H12	3	1	1	0	0	0	0	0	0	0	0.0000	72	113	76	0.6726	crchandbook	angell78	CCC(C)C	5A
4-methylcyclohexen	C7H12	1	3	3	0	0	0	0	2	0	0	0.0000	96	158	94	0.5949	bradleydataset	angell78	CC1CCC=CC1	
acetaldehyd	C2H4O	1	0	0	1	0	0	1	1	0	0	0.5000	44	149	82	0.5503	bradleydataset	angell78	CC=O	
butyronitrile	C4H7N	1	2	0	1	0	0	0	2	1	0	0.0000	69	161.25	97	0.6016	wangjchemphys06	wangjchemphys06	CCCC#N	
4-cyanoaniline	C7H6N2	0	0	4	3	0	0	0	6	2	0	0.0000	118	465	314	0.6753	tiersthermochimact93	tiersthermochimact93	C1=CC(=CC=C1C#N)N	
1-naphthylamine	C10H9N	0	0	7	3	0	0	0	7	1	0	0.0000	143	500	306	0.6120	tiersthermochimact93	tiersthermochimact93	C1=CC=C2C(=C1)C=CC=C2N	
2-naphthylamine	C10H9N	0	0	7	3	0	0	0	7	1	0	0.0000	143	458	304	0.6638	tiersthermochimact93	tiersthermochimact93	C1=CC=C2C=C(C=CC2=C1)N	6

Name	Formula	#CH3	#CH2	#CH	#C	#OH	#C-O-C	#O=C	DBA	#N	#Hal	O:C	M / g/mol	Tm / K	Tg / K	Tg/Tm	Reference(Tm)	Reference(Tg)	SMILES	Figure
m-toluidine	C7H9N	3	0	3	3	0	0	0	4	1	0	0.0000	107	242.8	187	0.7702	albasimionescojchemphys99	albasimionescojchemphys99	CC1=CC(=CC=C1)N	
triethylamine	C6H15N	3	3	0	0	0	0	0	0	1	0	0.0000	101	158.45	103.15	0.6510	lesikarjchemphys77	lesikarjchemphys77	CCN(CC)CC	5B
pyridine	C5H5N	0	0	5	0	0	0	0	4	1	0	0.0000	79	231.45	127.45	0.5507	lesikarjchemphys77	lesikarjchemphys77	C1=CC=NC=C1	
2-methoxy-4-nitroaniline	C7H8N2O3	1	0	3	3	0	1	2	5	2	0	0.4286	168	457	330.5	0.7232	tiersthermochimact93	tiersthermochimact93	COC1=C(C=CC(=C1)[N+](=O)[O-])N	
4-nitroaniline	C6H6N2O2	0	0	4	2	0	0	2	5	2	0	0.3333	138	452	318	0.7035	tiersthermochimact93	tiersthermochimact93	C1=CC(=CC=C1N)[N+](=O)[O-]	5B
4-nitro-1-naphthylamine	C10H8N2O2	0	0	6	4	0	0	2	8	2	0	0.2000	188	448	305	0.6808	tiersthermochimact93	tiersthermochimact93	C1=CC=C2C(=C1)C(=CC=C2[N+](=O)[O-])N	5B
2-nitroaniline	C6H6N2O2	0	0	4	2	0	0	2	5	2	0	0.3333	138	454	299	0.6586	tiersthermochimact93	tiersthermochimact93	C1=CC=C(C(=C1)N)[N+](=O)[O-]	5B
3-nitroaniline	C6H6N2O2	0	0	4	2	0	0	2	5	2	0	0.3333	138	431	301	0.6984	tiersthermochimact93	tiersthermochimact93	C1=CC(=CC(=C1)[N+](=O)[O-])N	5B
4-methoxyaniline	C7H9NO	1	0	4	2	0	1	0	4	1	0	0.1429	123	453	287	0.6336	tiersthermochimact93	tiersthermochimact93	COC1=CC=C(C=C1)N	
cinchonine	C19H22N2O	0	5	11	3	1	0	0	10	2	0	0.0526	194	522	395	0.7567	tiersthermochimact93	tiersthermochimact93	C=CC1CN2CCC1CC2C(C3=CC=NC4=CC=CC=C34)O	
brucine	C23H26N2O4	2	6	8	7	0	3	1	12	2	0	0.1739	394	451	371	0.8226	tiersthermochimact93	tiersthermochimact93	COC1=C(C=C2C(=C1)C34CCN5C3CC6C7C4N2C(=O)CC7OCC=C6C5)OC	
cinchonidine	C19H22N2O	0	5	11	3	1	0	0	10	2	0	0.0526	194	476	377	0.7920	tiersthermochimact93	tiersthermochimact93	C=CC1CN2CCC1CC2C(C3=CC=NC4=CC=CC=C34)O	
piperine	C17H19NO3	0	6	7	4	0	2	1	9	1	0	0.1765	285	401	284	0.7082	tiersthermochimact93	tiersthermochimact93	C1CCN(CC1)C(=O)C=CC=CC2=CC3=C(C=C2)OCO3	
benzyl n-phenylcarbamate	C13H11NO2	0	1	10	3	0	1	1	9	1	0	0.1429	227	354	233	0.6582	naitojphychem93	naitojphychem93	C1=CC=C(C=C1)COC(=O)NC2=CC=CC=C2	
methyl n-phenylcarbamate	C8H9NO2	1	0	5	2	0	1	1	5	1	0	0.2500	151	325	218	0.6708	naitojphychem93	naitojphychem93	COC(=O)NC1=CC=CC=C1	
ethyl n-phenylcarbamate	C9H11NO2	1	1	5	2	0	1	1	5	1	0	0.2222	165	326	213	0.6534	naitojphychem93	naitojphychem93	CCOC(=O)NC1=CC=CC=C1	
propyl n-phenylcarbamate	C10H13NO2	1	2	5	2	0	1	1	5	1	0	0.2000	179	331	203	0.6133	naitojphychem93	naitojphychem93	CCCOC(=O)NC1=CC=CC=C1	
dibucaine	C20H29N3O2	3	7	5	5	0	1	1	8	3	0	0.1000	343	336	246	0.7321	fukuokachempharmbull91	fukuokachempharmbull91	CCCCOC1=NC2=CC=CC=C2C(=C1)C(=O)NCCN(CC)CC	6
proxyphylline	C10H14N4O3	3	1	2	4	1	0	2	6	4	0	0.3000	238	403	295	0.7320	fukuokachempharmbull91	fukuokachempharmbull91	CC(CN1C=NC2=C1C(=O)N(C(=O)N2C)C)O	6
eserine	C15H21N3O2	4	2	4	5	0	1	1	7	3	0	0.1333	275	378	297	0.7857	fukuokachempharmbull91	fukuokachempharmbull91	CC12CCN(C1N(C3=C2C=C(C=C3)OC(=O)NC)C)C	
nialamide	C16H18N4O2	0	3	9	4	0	0	2	10	3	0	0.1250	298	427	297	0.6956	fukuokachempharmbull91	fukuokachempharmbull91	C1=CC=C(C=C1)CNC(=O)CCNNC(=O)C2=CC=NC=C2	
acetaminophen	C8H9NO2	1	0	4	3	1	0	1	5	1	0	0.2500	151	441	302	0.6848	fukuokachempharmbull91	fukuokachempharmbull91	CC(=O)NC1=CC=C(C=C1)O	
diphylline	C10H14N4O4	2	2	2	4	2	0	2	6	4	0	0.4000	254	438	315	0.7192	fukuokachempharmbull91	fukuokachempharmbull91	CN1C2=C(C(=O)N(C1=O)C)N(C=N2)CC(CO)O	5B
brucine	C23H26N2O4	2	6	8	7	0	3	1	12	2	0	0.1739	394	451	365	0.8093	fukuokachempharmbull91	fukuokachempharmbull91	COC1=C(C=C2C(=C1)C34CCN5C3CC6C7C4N2C(=O)CC7OCC=C6C5)OC	
acetaminophen	C8H9NO2	1	0	4	3	1	0	1	5	1	0	0.2500	151	447	295	0.6600	kercthermochimacta95	kercthermochimacta95	CC(=O)NC1=CC=C(C=C1)O	
nitrendipine	C18H20N2O6	4	1	4	9	0	2	4	10	2	0	0.3333	360	429	303	0.7063	kercthermochimacta95	kercthermochimacta95	CCOC(=O)C1=C(NC(=C(C1C2=CC(=CC=C2)[N+](=O)[O-])C(=O)OC)C)C	
phenobarbital	C12H12N2O3	1	1	5	5	0	0	3	8	2	0	0.2500	232	452	315	0.6969	kercthermochimacta95	kercthermochimacta95	CCC1(C(=O)NC(=O)NC1=O)C2=CC=CC=C2	
phenobarbital	C12H12N2O3	1	1	5	5	0	0	3	8	2	0	0.2500	232	443	321	0.7246	kercthermochimacta95	kercthermochimacta95	CCC1(C(=O)NC(=O)NC1=O)C2=CC=CC=C2	
quinidine	C20H24N2O2	1	5	10	4	1	1	0	10	2	0	0.1000	324	445	326	0.7326	kercthermochimacta95	kercthermochimacta95	COC1=CC2=C(C=CN=C2C=C1)C(C3CC4CCN3CC4C=C)O	
antipyrine	C11H12N2O	2	0	6	3	0	0	1	7	2	0	0.0909	188	380	256	0.6737	kercthermochimacta95	kercthermochimacta95	CC1=CC(=O)N(N1C)C2=CC=CC=C2	5B
phenylbutazone	C19H20N2O2	1	3	11	4	0	0	2	11	2	0	0.1053	308	377	277	0.7347	kercthermochimacta95	kercthermochimacta95	CCCCC1C(=O)N(N(C1=O)C2=CC=CC=C2)C3=CC=CC=C3	
quinineethylcarbonate	C23H28N2O4	2	6	10	5	0	3	1	11	2	0	0.1739	396	362	278	0.7680	kercthermochimacta95	kercthermochimacta95	CCOC(=O)OC(C1CC2CCN1CC2C=C)C3=C4C=C(C=CC4=NC=C3)OC	
pentobarbital	C11H18N2O3	3	3	1	4	0	0	3	4	2	0	0.2727	226	408	279	0.6838	kercthermochimacta95	kercthermochimacta95	CCCC(C)C1(C(=O)NC(=O)NC1=O)CC	
atropine	C17H23NO3	1	5	9	2	1	1	1	7	1	0	0.1765	289	379	281	0.7414	kercthermochimacta95	kercthermochimacta95	CN1C2CCC1CC(C2)OC(=O)C(CO)C3=CC=CC=C3	
hexobarbital	C12H16N2O3	2	4	1	5	0	0	3	6	2	0	0.2500	236	423	286	0.6761	kercthermochimacta95	kercthermochimacta95	CC1(C(=O)NC(=O)N(C1=O)C)C2=CCCCC2	
amobarbital	C11H18N2O3	3	3	1	4	0	0	3	4	2	0	0.2727	226	432	286	0.6620	kercthermochimacta95	kercthermochimacta95	CCC1(C(=O)NC(=O)NC1=O)CCC(C)C	
nimodipine	C21H26N2O7	5	2	6	8	0	3	4	10	2	0	0.3333	418	389	288	0.7404	kercthermochimacta95	kercthermochimacta95	CC1=C(C(C(=C(N1)C)C(=O)OC(C)C)C2=CC(=CC=C2)[N+](=O)[O-])C(=O)OCCOC	
dimethyl acetamide	C4H9NO	3	0	0	1	0	0	1	1	1	0	0.2500	87	254.56	146.15	0.5741	lesikarjchemphys77	lesikarjchemphys77	CC(=O)N(C)C	5B
1-bromobutane	C4H9Br	1	3	0	0	0	0	0	0	0	1	0.0000	137	160.55	96.5	0.6011	wangjphyschemb07	wangjphyschemb07	CCCCBr	
isobutylbromide	C4H9Br	2	1	1	0	0	0	0	0	0	1	0.0000	137.018	154.15	99.1	0.6429	wangjphyschemb07	wangjphyschemb07	CC(C)CBr	
isoamylbromide	C5H11Br	2	2	1	0	0	0	0	0	0	1	0.0000	151.045	161.15	109	0.6764	wangjphyschemb07	wangjphyschemb07	CC(C)CCBr	
3-bromopentane	C5H11Br	2	2	1	0	0	0	0	0	0	1	0.0000	151	146.95	106	0.7213	wangjchemphys06	wangjchemphys06	CCC(Br)CC	
9-bromophenanthrene	C14H9Br	0	0	9	5	0	0	0	12	0	1	0.0000	257	337.65	224.8	0.6658	wangjchemphys06	wangjchemphys06	C1=CC=C2C(=C1)C=C(C3=CC=CC=C23)Br	
bromobenzene	C6H5Br	0	0	5	1	0	0	0	4	0	1	0.0000	157.008	243	140	0.5761	naitojphychem93	naitojphychem93	C1=CC=C(C=C1)Br	5B
tert-butyl-chloride	C4H9Cl	3	0	0	1	0	0	0	0	0	1	0.0000	92	248	130	0.5242	naitojphychem93	naitojphychem93	CC(C)(C)Cl	5B, 6
1 -bromopropane	C3H7Br	1	2	0	0	0	0	0	0	0	1	0.0000	122.992	160.3	107	0.6675	murthyjchemsocfaradaytrans93	murthyjchemsocfaradaytrans93	CCCBr	
1-bromobutane	C4H9Br	1	3	0	0	0	0	0	0	0	1	0.0000	137.018	159.2	103.3	0.6489	murthyjchemsocfaradaytrans93	murthyjchemsocfaradaytrans93	CCCCBr	
1-iodobutane	C4H9I	1	3	0	0	0	0	0	0	0	1	0.0000	184.018	164.6	108.4	0.6586	murthyjchemsocfaradaytrans93	murthyjchemsocfaradaytrans93	CCCCI	
isobutylchloride	C4H9Cl	2	1	1	0	0	0	0	0	0	1	0.0000	92	142.7	89.4	0.6265	murthyjchemsocfaradaytrans93	murthyjchemsocfaradaytrans93	CC(C)CCl	5B
tert-butyl-chloride	C4H9Cl	2	1	1	0	0	0	0	0	0	1	0.0000	92	247.5	137.5	0.5556	murthyjchemsocfaradaytrans93	murthyjchemsocfaradaytrans93	CC(C)(C)Cl	5B, 6
iodobenzene	C6H5I	0	0	5	1	0	0	0	4	0	1	0.0000	204.008	241.6	149.7	0.6196	murthyjchemsocfaradaytrans93	murthyjchemsocfaradaytrans93	C1=CC=C(C=C1)I	5B
3-bromopentane	C5H11Br	2	2	1	0	0	0	0	0	0	1	0.0000	151	146.8	110	0.7493	murthyjchemsocfaradaytrans93	murthyjchemsocfaradaytrans93	CCC(Br)CC	
m-fluorotoluene	C7H7F	1	0	4	2	0	0	0	4	0	1	0.0000	110.129	185	122.5	0.6622	albasimionescojchemphys99	albasimionescojchemphys99	CC1=CC(=CC=C1)F	
isobutylchloride	C4H9Cl	2	1	1	0	0	0	0	0	0	1	0.0000	92	142	88	0.6197	carpenterjchemphys67	carpenterjchemphys67	CC(C)CCl	5B
isobutylbromide	C4H9Br	2	1	1	0	0	0	0	0	0	1	0.0000	137.018	154.15	95	0.6163	carpenterjchemphys67	carpenterjchemphys67	CC(C)CBr	
isoamylbromide	C5H11Br	2	2	1	0	0	0	0	0	0	1	0.0000	151.045	161.3	105	0.6510	carpenterjchemphys67	carpenterjchemphys67	CC(C)CCBr	
trichloroethylene	C2HCl3	0	0	1	1	0	0	0	1	0	3	0.0000	131	188.45	107.05	0.5681	lesikarjchemphys75	lesikarjchemphys75	ClC=C(Cl)Cl	
hydrochloro-thiazide	C7H8ClN3O4S2	0	1	2	4	0	0	4	5	3	1	0.5714	297.74	547.15	385	0.7036	wangjchemphys06	wangjchemphys06	C1NC2=CC(=C(C=C2S(=O)(=O)N1)S(=O)(=O)N)Cl	5B
griseofulvin	C17H17ClO6	4	1	3	9	0	4	2	9	0	1	0.3529	352	493.15	364	0.7381	wangjchemphys06	wangjchemphys06	CC1CC(=O)C=C(C12C(=O)C3=C(O2)C(=C(C=C3OC)OC)Cl)OC	
glibenclamide	C23H28ClN3O5S	1	7	8	7	0	1	4	11	3	1	0.2174	494.004	445.65	331	0.7427	wangjchemphys06	wangjchemphys06	COC1=C(C=C(C=C1)Cl)C(=O)NCCC2=CC=C(C=C2)S(=O)(=O)NC(=O)NC3CCCCC3	
triphenyl phosphite	C18H15O3P	0	0	15	3	0	3	0	12	1	0	0.1667	310.284	298.15	200	0.6708	wangjchemphys06	wangjchemphys06	C1=CC=C(C=C1)OP(OC2=CC=CC=C2)OC3=CC=CC=C3	
2-chloro-4-nitroaniline	C6H5ClN2O2	0	0	3	3	0	0	2	5	2	1	0.3333	172	441	324	0.7347	tiersthermochimact93	tiersthermochimact93	C1=CC(=C(C=C1[N+](=O)[O-])Cl)N	
4-chloroaniline	C6H6ClN	0	0	4	2	0	0	0	4	1	1	0.0000	127	463	297	0.6415	tiersthermochimact93	tiersthermochimact93	C1=CC(=CC=C1N)Cl	6
3,4-dichloroaniline	C6H5Cl2N	0	0	3	3	0	0	0	4	1	2	0.0000	162	443	296	0.6682	tiersthermochimact93	tiersthermochimact93	C1=CC(=C(C=C1N)Cl)Cl	
2-chloroaniline	C6H6ClN	0	0	4	2	0	0	0	4	1	1	0.0000	127	435	292	0.6713	tiersthermochimact93	tiersthermochimact93	C1=CC=C(C(=C1)N)Cl	
3-chloroaniline	C6H6ClN	0	0	4	2	0	0	0	4	1	1	0.0000	127	416	286	0.6875	tiersthermochimact93	tiersthermochimact93	C1=CC(=CC(=C1)Cl)N	
3-trifluoromethylaniline	C7H6F3N	0	0	4	3	0	0	0	4	1	3	0.0000	161.125	397	280	0.7053	tiersthermochimact93	tiersthermochimact93	C1=CC(=CC(=C1)N)C(F)(F)F	
o-fluoroaniline	C6H6FN	0	0	4	2	0	0	0	4	1	1	0.0000	111.117	238.6	174	0.7293	albasimionescojchemphys99	albasimionescojchemphys99	C1=CC=C(C(=C1)N)F	6
m-fluorophenol	C6H5FO	0	0	4	2	1	0	0	4	0	1	0.1667	112.101	286	191	0.6678	albasimionescojchemphys99	albasimionescojchemphys99	C1=CC(=CC(=C1)F)O	
ethacrynic acid	C13H12Cl2O4	1	3	2	7	1	1	2	7	0	2	0.3077	303	398	282	0.7085	fukuokachempharmbull91	fukuokachempharmbull91	CCC(=C)C(=O)C1=C(C(=C(C=C1)OCC(=O)O)Cl)Cl	
tolbutamide	C12H18N2O3S	2	3	4	3	0	0	3	5	2	0	0.2500	270.347	403	284	0.7047	fukuokachempharmbull91	fukuokachempharmbull91	CCCCNC(=O)NS(=O)(=O)C1=CC=C(C=C1)C	
tolnaftate	C19H17NOS	2	0	11	6	0	0	0	12	1	0	0.0000	307.41	384	287	0.7474	fukuokachempharmbull91	fukuokachempharmbull91	CC1=CC(=CC=C1)N(C)C(=S)OC2=CC3=CC=CC=C3C=C2	
flufenamic acid	C14H10F3NO2	0	0	8	6	1	0	1	9	1	3	0.1429	281.23	406	290	0.7143	fukuokachempharmbull91	fukuokachempharmbull91	C1=CC=C(C(=C1)C(=O)O)NC2=CC=CC(=C2)C(F)(F)F	
chorotrianisene	C23H21ClO3	3	0	12	8	0	3	0	13	0	1	0.1304	380	393	298	0.7583	fukuokachempharmbull91	fukuokachempharmbull91	COC1=CC=C(C=C1)C(=C(C2=CC=C(C=C2)OC)Cl)C3=CC=C(C=C3)OC	
chloramphenicol	C11H12Cl2N2O5	0	1	7	3	2	0	3	6	2	2	0.4545	323	414	306	0.7391	fukuokachempharmbull91	fukuokachempharmbull91	C1=CC(=CC=C1C(C(CO)NC(=O)C(Cl)Cl)O)[N+](=O)[O-]	
spironolactone	C24H32O4S	3	9	5	7	0	1	3	9	0	0	0.1667	416.574	478	331	0.6925	fukuokachempharmbull91	fukuokachempharmbull91	CC(=O)SC1CC2=CC(=O)CCC2(C3C1C4CCC5(C4(CC3)C)CCC(=O)O5)C	
chlormadinone acetate	C23H29ClO4	4	6	5	7	0	1	3	9	0	1	0.1818	404	483	334	0.6915	fukuokachempharmbull91	fukuokachempharmbull91	CC(=O)C1(CCC2C1(CCC3C2C=C(C4=CC(=O)CCC34C)Cl)C)OC(=O)C	
griseofulvin	C17H17ClO6	4	1	3	9	0	4	2	9	0	1	0.3529	352	497	370	0.7445	fukuokachempharmbull91	fukuokachempharmbull91	CC1CC(=O)C=C(C12C(=O)C3=C(O2)C(=C(C=C3OC)OC)Cl)OC	
indomethacin	C19H16ClNO4	2	1	7	9	1	1	2	12	1	1	0.2105	357	438	320	0.7306	hancockjpharmsci97	hancockjpharmsci97	CC1=C(C2=C(N1C(=O)C3=CC=C(C=C3)Cl)C=CC(=C2)OC)CC(=O)O	
chloroamphenicol	C11H12Cl2N2O5	0	1	7	3	2	0	3	6	2	2	0.4545	323	425	301	0.7082	alzghoul2014	kercthermochimacta95	C1=CC(=CC=C1C(C(CO)NC(=O)C(Cl)Cl)O)[N+](=O)[O-]	
chloramphenicol	C11H12Cl2N2O5	0	1	7	3	2	0	3	6	2	2	0.4545	323	425	304	0.7153	alzghoul2014	alzghoul2014	C1=CC(=CC=C1C(C(CO)NC(=O)C(Cl)Cl)O)[N+](=O)[O-]	
sulphisoxazole	C11H13N3O3S	2	0	4	5	0	1	2	7	1	0	0.2727	267.304	460	306	0.6652	kercthermochimacta95	kercthermochimacta95	CC1=C(ON=C1C)NS(=O)(=O)C2=CC=C(C=C2)N	
felodipine	C18H19Cl2NO4	4	1	4	9	0	2	2	9	1	2	0.2222	384	407	316	0.7764	kercthermochimacta95	kercthermochimacta95	CCOC(=O)C1=C(NC(=C(C1C2=C(C(=CC=C2)Cl)Cl)C(=O)OC)C)C	
sulphathiazole	C9H9N3O2S2	0	0	6	3	0	0	2	7	3	0	0.2222	255.316	471	334	0.7091	kercthermochimacta95	kercthermochimacta95	C1=CC(=CC=C1N)S(=O)(=O)NC2=NC=CS2	
sulphadimethoxine	C12H14N4O4S	2	0	5	5	0	2	2	8	4	0	0.3333	310.329	465	339	0.7290	kercthermochimacta95	kercthermochimacta95	COC1=NC(=NC(=C1)NS(=O)(=O)C2=CC=C(C=C2)N)OC	
glibenclamide	C23H28ClN3O5S	1	7	8	7	0	1	4	11	3	1	0.2174	494.004	447	344	0.7696	kercthermochimacta95	kercthermochimacta95	COC1=C(C=C(C=C1)Cl)C(=O)NCCC2=CC=C(C=C2)S(=O)(=O)NC(=O)NC3CCCCC3	
proxyphylline	C10H14N4O3	3	1	2	4	1	0	2	5	4	0	0.3000	238	403	295	0.7320	kercthermochimacta95	kercthermochimacta95	CC(CN1C=NC2=C1C(=O)N(C(=O)N2C)C)O	6
acetaminophen	C8H9NO2	1	0	4	3	1	0	1	5	1	0	0.2500	151	347	297	0.8559	kercthermochimacta95	kercthermochimacta95	CC(=O)NC1=CC=C(C=C1)O	
eserine	C15H21N3O2	4	2	4	5	0	1	1	7	3	0	0.1333	275	378	297	0.7857	kercthermochimacta95	kercthermochimacta95	CC12CCN(C1N(C3=C2C=C(C=C3)OC(=O)NC)C)C	
nialamide	C16H18N4O2	0	3	9	4	0	0	2	10	4	0	0.1250	298	427	297	0.6956	kercthermochimacta95	kercthermochimacta95	C1=CC=C(C=C1)CNC(=O)CCNNC(=O)C2=CC=NC=C2	
chlorotrianisene	C23H21ClO3	3	0	12	8	0	3	0	13	0	1	0.1304	380	393	298	0.7583	kercthermochimacta95	kercthermochimacta95	COC1=CC=C(C=C1)C(=C(C2=CC=C(C=C2)OC)Cl)C3=CC=C(C=C3)OC	

Name	Formula	#CH3	#CH2	#CH	#C	#OH	#C-O-C	#O=C	DBA	#N	#Hal	O:C	M / g/mol	Tm / K	Tg / K	Tg/Tm	Reference(Tm)	Reference(Tg)	SMILES	Figure
acetaminophen	C8H9NO2	1	0	4	3	1	0	1	5	1	0	0.2500	151	441	302	0.6848	kercthermochimacta95	kercthermochimacta95	CC(=O)NC1=CC=C(C=C1)O	
chloramphenicol	C11H12Cl2N2O5	0	1	7	3	2	0	3	6	2	2	0.4545	323	414	306	0.7391	kercthermochimacta95	kercthermochimacta95	C1=CC(=CC=C1C(C(CO)NC(=O)C(Cl)Cl)O)[N+](=O)[O-]	
diphylline	C10H14N4O4	2	2	2	4	2	0	2	6	4	0	0.4000	254	438	315	0.7192	kercthermochimacta95	kercthermochimacta95	CN1C2=C(C(=O)N(C1=O)C)N(C=N2)CC(CO)O	5B
spironolactone	C24H32O4S	3	9	5	7	0	1	3	9	0	0	0.1667	416.574	478	331	0.6925	kercthermochimacta95	kercthermochimacta95	CC(=O)SC1CC2=CC(=O)CCC2(C3C1C4CCC5(C4(CC3)C)CCC(=O)O5)C	
chlormadinone acetate	C23H29ClO4	4	6	5	7	0	1	3	9	0	1	0.1818	404	483	334	0.6915	kercthermochimacta95	kercthermochimacta95	CC(=O)C1(CCC2C1(CCC3C2C=C(C4=CC(=O)CCC34C)Cl)C)OC(=O)C	
griseofulvin	C17H17ClO6	4	1	3	9	0	4	2	9	0	1	0.3529	352	422	362	0.8578	kercthermochimacta95	kercthermochimacta95	CC1CC(=O)C=C(C12C(=O)C3=C(O2)C(=C(C=C3OC)OC)Cl)OC	
brucine	C23H26N2O4	2	6	8	7	0	3	1	12	2	0	0.1739	394	451	365	0.8093	kercthermochimacta95	kercthermochimacta95	COC1=C(C=C2C(=C1)C34CCN5C3CC6C7C4N2C(=O)CC7OCC=C6C5)OC	
griseofulvin	C17H17ClO6	4	1	3	9	0	4	2	9	0	1	0.3529	352	497	370	0.7445	kercthermochimacta95	kercthermochimacta95	CC1CC(=O)C=C(C12C(=O)C3=C(O2)C(=C(C=C3OC)OC)Cl)OC	
dibucaine	C20H29N3O2	3	7	5	5	0	1	1	8	3	0	0.1000	343	336	246	0.7321	kercthermochimacta95	kercthermochimacta95	CCCCOC1=NC2=CC=CC=C2C(=C1)C(=O)NCCN(CC)CC	6
ethacrynic acid	C13H12Cl2O4	1	3	2	7	1	1	2	7	0	2	0.3077	303	398	282	0.7085	kercthermochimacta95	kercthermochimacta95	CCC(=C)C(=O)C1=C(C(=C(C=C1)OCC(=O)O)Cl)Cl	
tolbutamide	C12H18N2O3S	2	3	4	3	0	0	3	5	2	0	0.2500	270.347	403	284	0.7047	kercthermochimacta95	kercthermochimacta95	CCCCNC(=O)NS(=O)(=O)C1=CC=C(C=C1)C	
tolnaphtate	C19H17NOS	2	0	11	6	0	0	0	12	1	0	0.0000	307.41	384	287	0.7474	kercthermochimacta95	kercthermochimacta95	CC1=CC(=CC=C1)N(C)C(=S)OC2=CC3=CC=CC=C3C=C2	
flufenamic acid	C14H10F3NO2	0	0	8	6	1	0	1	9	1	3	0.1429	281.23	406	290	0.7143	kercthermochimacta95	kercthermochimacta95	C1=CC=C(C(=C1)C(=O)O)NC2=CC=CC(=C2)C(F)(F)F	
chlorobenzene	C6H5Cl	0	0	5	1	0	0	0	4	0	1	0.0000	112	226.6	126.3	0.5574	murthyjchemsocfaradaytrans93	murthyjchemsocfaradaytrans93	C1=CC=C(C=C1)Cl	
isobutylbromide	C4H9Br	2	1	1	0	0	0	0	0	0	1	0.0000	137	156	85	0.5449	naitojphychem93	naitojphychem93	CC(C)CBr	
isobutylbromide	C4H9Br	2	1	1	0	0	0	0	0	0	1	0.0000	137	155.6	97	0.6234	murthyjchemsocfaradaytrans93	murthyjchemsocfaradaytrans93	CC(C)CBr	
isobutylbromide	C4H9Br	2	1	1	0	0	0	0	0	0	1	0.0000	137	156	85	0.5449	naitojphychem93	naitojphychem93	CC(C)CBr	
m-fluorotoluene	C7H7F	1	0	4	2	0	0	0	4	0	1	0.0000	110.129	186.15	122.5	0.6581	wangjchemphys06	wangjchemphys06	CC1=CC(=CC=C1)F	
m-toluidine	C7H9N	3	0	3	3	0	0	0	4	1	0	0.0000	107	241.85	187	0.7732	wangjchemphys06	wangjchemphys06	CC1=CC(=CC=C1)N	
probucol	C31H48O2S2	14	0	4	13	2	0	0	8	0	0	0.0645	516	398.15	295	0.7409	bradleydataset	wangjchemphys06	CC(C)(C)C1=CC(=CC(=C1O)C(C)(C)C)SC(C)(C)SC2=CC(=C(C(=C2)C(C)(C)C)O)C(C)(C)C	5B
phenobarbital	C12H12N2O3	1	1	5	5	0	0	3	8	2	0	0.2500	232	447.15	319	0.7134	wangjchemphys06	wangjchemphys06	CCC1(C(=O)NC(=O)NC1=O)C2=CC=CC=C2	
indomethacin	C19H16ClNO4	2	1	7	9	1	1	2	12	1	1	0.2105	357	431.15	318	0.7376	wangjchemphys06	wangjchemphys06	CC1=C(C2=C(N1C(=O)C3=CC=C(C=C3)Cl)C=CC(=C2)OC)CC(=O)O	
3-bromopentane	C5H11Br	2	2	1	0	0	0	0	0	0	1	0.0000	151	146.95	108	0.7349	wangjphyschemb07	wangjphyschemb07	CCC(Br)CC	
2-ethylhexylamine	C8H19N	2	5	1	0	0	0	0	0	1	0	0.0000	129	197	141	0.7157	bradleydataset	wangjchemphys08	CCCCC(CC)CN	
n-(s-1-naphthylethyl)-tetrachlorophthalimide	C20H13O2NCl4	1	0	10	9	0	0	2	14	1	4	0.1000	439	462	362	0.7835	tiersthermochimact93	tiersthermochimact93	CC(c2ccc1ccccc1c2)n4c(=O)c3c(Cl)c(Cl)c(Cl)c(Cl)c3c4=O	
n-(s-1-naphthylethyl)-2,2′-diphenic imide	C26H19O2N	1	0	16	9	0	0	2	15	1	0	0.0769	377	469	356	0.7591	tiersthermochimact93	tiersthermochimact93	CC(c2ccc1ccccc1c2)n5c(=O)c3ccccc3c4ccccc4c5=O	
n-(r-1-phenylethyl)-2,2′-diphenic imide	C22H17O2N	1	0	14	7	0	0	2	15	1	0	0.0909	327	483	333	0.6894	tiersthermochimact93	tiersthermochimact93	CC(c1ccccc1)n4c(=O)c2ccccc2c3ccccc3c4=O	6
n-(r-1-phenylethyl)-4-nitro-1,8-naphthalimide	C20H14O4N2	1	0	11	8	0	0	4	15	2	0	0.2000	346	401	320	0.7980	tiersthermochimact93	tiersthermochimact93	CC(c1ccccc1)n2c(=O)c4cccc3c(N(=O)=O)ccc(c2=O)c34	
n-(r-1-phenylethyl)-1,8-naphthalimide	C20H15O2N	1	0	12	7	0	0	2	15	1	0	0.1000	300	435	319	0.7333	tiersthermochimact93	tiersthermochimact93	CC(c1ccccc1)n2c(=O)c4cccc3cccc(c2=O)c34	
n-(r-1-phenylethyl)-3-nitrophthalimide	C16H12O4N2	1	0	9	6	0	0	4	12	2	0	0.2500	296	395	289	0.7316	tiersthermochimact93	tiersthermochimact93	CC(c1ccccc1)n3c(=O)c2cccc(N(=O)=O)c2c3=O	6
4-acetamido-n-(2′,4′-dimethylphenyl)-1,8naphthalimide	C21H18O3N2	3	0	8	10	0	0	3	15	2	0	0.1429	346	564	401	0.7110	tiersthermochimact93	tiersthermochimact93	CC(=O)Nc2ccc4c(=O)n(c1ccc(C)cc1C)c(=O)c3cccc2c34	5B
n-(1,2,3,4-tetrahydro-1-naphthyl)-tetrachlorophthalimide	C18H11O2NCl4	0	3	5	10	0	0	2	12	1	4	0.1111	413	501	343	0.6846	tiersthermochimact93	tiersthermochimact93	O=c2c1c(Cl)c(Cl)c(Cl)c(Cl)c1c(=O)n2c3cccc4ccccc34	5B
n-(r-1-phenylethyl)-2,2′-diphenic imide	C22H17O2N	1	0	14	7	0	0	2	15	1	0	0.0909	327	475	333	0.7011	tiersthermochimact93	tiersthermochimact93	CC(c1ccccc1)n4c(=O)c2ccccc2c3ccccc3c4=O	6
1-methyl-1-(p-hydroxyphenyl)-1,2,3,4tetrahydronaphthalene	C17H18O	1	3	8	5	1	0	0	9	0	0	0.0588	238	393	283	0.7201	tiersthermochimact93	tiersthermochimact93	CC3(c1ccc(O)cc1)CCCc2ccccc23	6
1,3-dinitro-4,6-bis-(r-l′-phenylethylamino)benzene	C22H22N4O4	2	0	14	6	0	0	3	14	4	0	0.1364	406	469	316	0.6738	tiersthermochimact93	tiersthermochimact93	CC(Nc2cc(NC(C)c1ccccc1)c(N(=O)=O)cc2N(=O)=O)c3ccccc3	
n-(r-1-phenylethyl)-n′-(p-nitrophenyl)thiourea	C15H15N3O2S	1	0	10	4	0	0	3	10	3	0	0.2000	301	386	313	0.8109	tiersthermochimact93	tiersthermochimact93	CC(NC(=S)Nc1ccc(N(=O)=O)cc1)c2ccccc2	
1-menthyl-3-nitrophenylurethane	C17H23N2O4	3	3	8	3	0	1	3	6	2	0	0.2353	319	354	288	0.8136	tiersthermochimact93	tiersthermochimact93	CC(C)C1CCC(C)CC1OC(=O)Nc2cccc(N(=O)=O)c2	
1′-(ethoxycarbonyl)ethyl-2-methyl-4-nitrophenylurethane	C14H20N2O6	3	4	3	4	0	2	4	7	2	0	0.4286	312	343	273	0.7959	tiersthermochimact93	tiersthermochimact93	CCOC(=O)CCC(C(=O)OCC)c1ccc(N(=O)=O)cc1C	
1,1,3-trimethyl-3-(p-carboxyphenyl)-indan-5-carboxylic acid	C20H20O4	3	1	7	9	2	0	2	11	0	0	0.2000	324	565	402	0.7115	tiersthermochimact93	tiersthermochimact93	CC3(C)CC(C)(c1ccc(C(=O)O)cc1)c2cc(C(=O)O)ccc23	
2-benzyl-(3,3′)spirobi-(benzo[f]chroman)	C32H46O2	0	16	14	2	0	2	0	10	0	0	0.0625	462	483	339	0.7019	tiersthermochimact93	tiersthermochimact93	c7ccc(CC3CC2C(CCC1CCCCC12)OC36CCC5C(CCC4CCCCC45)O6)cc7	
n-(1-phenylethyl)-n′-(p-nitrophenyl)urea	C15H15N3O3	1	0	10	4	0	0	3	9	3	0	0.2000	285	444	305	0.6869	tiersthermochimact93	tiersthermochimact93	CC(NC(=O)Nc1ccc(N(=O)=O)cc1)c2ccccc2	
n-(1-phenylethyl)-2,4-dinitroaniline	C14H13N3O4	1	0	9	4	0	0	3	10	3	0	0.2143	287	387	283	0.7313	tiersthermochimact93	tiersthermochimact93	CC(Nc1ccc(N(=O)=O)cc1N(=O)=O)c2ccccc2	
n-(2-tetrahydropyryl)saccharin	C12H13NO4S	0	4	5	3	0	1	3	7	1	0	0.3333	267	409	279	0.6822	tiersthermochimact93	tiersthermochimact93	O=C2c1ccccc1S(=O)(=O)C2C3CCCOC3	
9,9-bis(p-hydroxyphenyl)fluorene	C25H18O2	0	0	16	9	2	0	0	17	0	0	0.0800	350	497	372	0.7485	tiersthermochimact93	tiersthermochimact93	Oc5ccc(C4(c1ccc(O)cc1)c2ccccc2c3ccccc34)cc5	
9,9-bis(p-aminophenyl)fluorene	C25H20N2	0	0	16	9	0	0	0	17	2	0	0.0000	348	501	360	0.7186	tiersthermochimact93	tiersthermochimact93	Nc5ccc(C4(c1ccc(N)cc1)c2ccccc2c3ccccc34)cc5	
anilinephthalein	C20H16N2O2	0	0	12	8	0	1	1	12	2	0	0.1000	316	475	353	0.7432	tiersthermochimact93	tiersthermochimact93	Nc4ccc(C3(c1ccc(N)cc1)OC(=O)c2ccccc23)cc4	5B
2,2,2-tris(chloromethyl)ethyl-p-nitrophenylurethane	C12H13N2O4Cl3	0	4	4	4	0	1	3	6	2	3	0.3333	354	429	293	0.6830	tiersthermochimact93	tiersthermochimact93	O=C(Nc1ccc(N(=O)=O)cc1)OCC(CCl)(CCl)CCl	5B
2,2,3,3-tetrafluoropropyl-p-nitrophenylurethane	C10H8N2O4F4	0	1	5	4	0	1	3	6	2	4	0.4000	296	348	261	0.7500	tiersthermochimact93	tiersthermochimact93	O=C(Nc1ccc(N(=O)=O)cc1)OCC(F)(F)C(F)F	
n-pentyl-p-nitrophenylurethane	C12H16N2O4	1	4	4	3	0	1	3	6	2	0	0.3333	210	365	243	0.6658	tiersthermochimact93	tiersthermochimact93	CCCCCOC(=O)Nc1ccc(N(=O)=O)cc1	
budesonide	C25H34O6	3	7	9	6	2	2	2	9	0	0	0.2400	430	530	368	0.6943	alzghoul2014	alzghoul2014	CCCC1OC2CC3C4CCC5=CC(=O)C=CC5(C4C(CC3(C2(O1)C(=O)CO)C)O)C	7
prednisone	C21H26O5	2	6	6	7	2	0	3	9	0	0	0.2381	358	513	366	0.7135	alzghoul2014	alzghoul2014	CC12CC(=O)C3C(C1CCC2(C(=O)CO)O)CCC4=CC(=O)C=CC34C	7
hydrocortisone	C21H30O5	2	8	5	6	3	0	2	7	0	0	0.2381	362	497	359	0.7223	alzghoul2014	alzghoul2014	CC12CCC(=O)C=C1CCC3C2C(CC4(C3CCC4(C(=O)CO)O)C)O	7
warfarin	C19H16O4	1	1	10	7	1	1	2	12	0	0	0.2105	308	435	341	0.7839	alzghoul2014	alzghoul2014	CC(=O)CC(C1=CC=CC=C1)C2=C(C3=CC=CC=C3OC2=O)O	7
testosterone	C19H28O2	2	8	5	4	1	0	1	6	0	0	0.1053	288	426	315	0.7394	alzghoul2014	alzghoul2014	CC12CCC3C(C1CCC2O)CCC4=CC(=O)CCC34C	7
nandrolone	C18H26O2	1	8	6	3	1	0	1	6	0	0	0.1111	274	397	310	0.7809	alzghoul2014	alzghoul2014	CC12CCC3C(C1CCC2O)CCC4=CC(=O)CCC34	7
simvastatin	C25H38O5	5	6	10	4	1	2	2	7	0	0	0.2000	418	412	309	0.7500	alzghoul2014	alzghoul2014	CCC(C)(C)C(=O)OC1CC(C=C2C1C(C(C=C2)C)CCC3CC(CC(=O)O3)O)C	7
fluorescamin	C17H10O4	0	0	10	7	0	2	2	13	0	0	0.2353	278	428	299	0.6986	alzghoul2014	alzghoul2014	C1=CC=C(C=C1)C2=COC3(C2=O)C4=CC=CC=C4C(=O)O3	7
ibuprofen	C13H18O2	3	1	6	3	1	0	1	5	0	0	0.1538	206	350	228	0.6514	alzghoul2014	alzghoul2014	CC(C)CC1=CC=C(C=C1)C(C)C(=O)O	7
ketoprofen	C16H14O3	1	0	10	5	1	0	2	10	0	0	0.1875	254	368	270	0.7337	alzghoul2014	alzghoul2014	CC(C1=CC(=CC=C1)C(=O)C2=CC=CC=C2)C(=O)O	7
Aripiprazole	C23H27Cl2N3O2	0	10	6	7	0	1	1	11	3	2	0.0870	448	526	363	0.6901	alzghoul2014	alzghoul2014	C1CC(=O)NC2=C1C=CC(=C2)OCCCCN3CCN(CC3)C4=C(C(=CC=C4)Cl)Cl	7
Bucindolol	C22H25N3O2	2	3	10	7	1	1	0	12	3	0	0.0909	364	459	356	0.7756	alzghoul2014	alzghoul2014	CC(C)(CC1=CNC2=CC=CC=C21)NCC(COC3=CC=CC=C3C#N)O	7
Danazol	C22H27NO2	2	7	6	7	1	1	0	10	1	0	0.0909	337	500	352	0.7040	alzghoul2014	alzghoul2014	CC12CCC3C(C1CCC2(C#C)O)CCC4=CC5=C(CC34C)C=NO5	7
Bezafibrate	C19H20ClNO4	2	2	8	7	1	1	2	10	1	0	0.2105	362	457	346	0.7571	alzghoul2014	alzghoul2014	CC(C)(C(=O)O)OC1=CC=C(C=C1)CCNC(=O)C2=CC=C(C=C2)Cl	7
Ezetimibe	C24H21F2NO3	0	2	15	7	2	0	1	14	1	2	0.1250	409	437	338	0.7735	alzghoul2014	alzghoul2014	C1=CC(=CC=C1C2C(C(=O)N2C3=CC=C(C=C3)F)CCC(C4=CC=C(C=C4)F)O)O	7
Glafenine	C19H17ClN2O4	0	2	10	7	2	1	1	12	2	1	0.2105	373	437	336	0.7689	alzghoul2014	alzghoul2014	C1=CC=C(C(=C1)C(=O)OCC(CO)O)NC2=C3C=CC(=CC3=NC=C2)Cl	7
Pimozide	C28H29F2N3O	0	7	14	7	0	0	1	14	3	2	0.0357	461	492	327	0.6646	alzghoul2014	alzghoul2014	C1CN(CCC1N2C3=CC=CC=C3NC2=O)CCCC(C4=CC=C(C=C4)F)C5=CC=C(C=C5)F	7
Zolmitriptan	C16H21N3O2	2	4	5	5	0	1	1	8	3	0	0.1250	287	410	322	0.7854	alzghoul2014	alzghoul2014	CN(C)CCC1=CNC2=C1C=C(C=C2)CC3COC(=O)N3	7
Diazepam	C16H13ClN2O	1	1	8	6	0	0	1	7	2	1	0.0625	284	404	319	0.7896	alzghoul2014	alzghoul2014	CN1C(=O)CN=C(C2=C1C=CC(=C2)Cl)C3=CC=CC=C3	7
Felodipine	C18H19Cl2NO4	4	1	4	9	0	2	2	9	1	2	0.2222	384	420	318	0.7571	alzghoul2014	alzghoul2014	CCOC(=O)C1=C(NC(=C(C1C2=C(C(=CC=C2)Cl)Cl)C(=O)OC)C)C	7
Nifedipine	C17H18N2O6	4	0	5	8	0	2	4	9	1	0	0.3529	346	446	318	0.7130	alzghoul2014	alzghoul2014	CC1=C(C(C(=C(N1)C)C(=O)OC)C2=CC=CC=C2[N+](=O)[O-])C(=O)OC	7
Carvedilol	C24H26N2O4	1	4	12	7	1	3	0	13	2	0	0.1667	407	390	315	0.8077	alzghoul2014	alzghoul2014	COC1=CC=CC=C1OCCNCC(COC2=CC=CC3=C2C4=CC=CC=C4N3)O	7
Loratadine	C22H23ClN2O2	1	7	6	8	0	1	1	12	2	1	0.0909	383	409	310	0.7579	alzghoul2014	alzghoul2014	CCOC(=O)N1CCC(=C2C3=C(CCC4=C2N=CC=C4)C=C(C=C3)Cl)CC1	7
Clemastine	C21H26ClNO	2	5	10	4	0	1	0	9	1	1	0.0476	344	451	308	0.6829	alzghoul2014	alzghoul2014	CC(C1=CC=CC=C1)(C2=CC=C(C=C2)Cl)OCCC3CCCN3C	7
Clotrimazole	C22H17ClN2	0	0	17	5	0	0	0	15	2	1	0.0000	345	418	303	0.7249	alzghoul2014	alzghoul2014	C1=CC=C(C=C1)C(C2=CC=CC=C2)(C3=CC=CC=C3Cl)N4C=CN=C4	7
Physostigmine	C15H21N3O2	4	2	4	5	0	1	1	7	3	0	0.1333	275	377	293	0.7772	alzghoul2014	alzghoul2014	CC12CCN(C1N(C3=C2C=C(C=C3)OC(=O)NC)C)C	7
Bifonazole	C22H18N2	0	0	18	4	0	0	0	15	2	0	0.0000	310	424	290	0.6840	alzghoul2014	alzghoul2014	C1=CC=C(C=C1)C2=CC=C(C=C2)C(C3=CC=CC=C3)N4C=CN=C4	7
Cinnarizine	C26H28N2	0	5	18	3	0	0	0	14	2	0	0.0000	369	394	280	0.7107	alzghoul2014	alzghoul2014	C1CN(CCN1CC=CC2=CC=CC=C2)C(C3=CC=CC=C3)C4=CC=CC=C4	7
Clofoctol	C21H26Cl2O	5	2	6	8	1	0	0	8	0	2	0.0476	365	361	269	0.7452	alzghoul2014	alzghoul2014	CC(C)(C)CC(C)(C)C1=CC(=C(C=C1)O)CC2=C(C=C(C=C2)Cl)Cl	7
Tamoxifen	C26H29NO	3	3	14	6	0	1	0	13	1	0	0.0385	372	371	263	0.7089	alzghoul2014	alzghoul2014	CCC(=C(C1=CC=CC=C1)C2=CC=C(C=C2)OCCN(C)C)C3=CC=CC=C3	7
Procaine	C13H20N2O2	2	4	4	3	0	1	1	5	2	0	0.1538	236	335	234	0.6985	alzghoul2014	alzghoul2014	CCN(CC)CCOC(=O)C1=CC=C(C=C1)N	7
Orlistat	C29H53NO5	4	17	5	3	0	2	3	4	1	0	0.1724	496	316	228	0.7215	alzghoul2014	alzghoul2014	CCCCCCCCCCCC(CC1C(C(=O)O1)CCCCCC)OC(=O)C(CC(C)C)NC=O	7
Linaprazan	C21H26N4O2	4	3	5	9	1	0	1	11	4	0	0.0952	367	519	373	0.7187	alzghoul2014	alzghoul2014	CC1=C(C(=CC=C1)C)CNC2=CC(=CN3C2=NC(=C3C)C)C(=O)NCCO	7
Isradipine	C19H21N3O5	5	0	5	9	0	3	2	11	3	0	0.2632	371	432	316	0.7315	alzghoul2014	alzghoul2014	CC1=C(C(C(=C(N1)C)C(=O)OC(C)C)C2=CC=CC3=NON=C32)C(=O)OC	7
Acemetacin	C21H18ClNO6	2	2	7	10	1	2	3	13	1	1	0.2857	416	421	310	0.7363	alzghoul2014	alzghoul2014	CC1=C(C2=C(N1C(=O)C3=CC=C(C=C3)Cl)C=CC(=C2)OC)CC(=O)OCC(=O)O	7
Nilutamide	C12H10F3N3O4	2	0	3	7	0	0	4	7	3	3	0.3333	317	428	306	0.7150	alzghoul2014	alzghoul2014	CC1(C(=O)N(C(=O)N1)C2=CC(=C(C=C2)[N+](=O)[O-])C(F)(F)F)C	7
Aceclofenac	C16H13Cl2NO4	0	2	7	7	1	1	2	10	1	2	0.2500	354	426	283	0.6643	alzghoul2014	alzghoul2014	C1=CC=C(C(=C1)CC(=O)OCC(=O)O)NC2=C(C=CC=C2Cl)Cl	7
Miconazole	C18H14Cl4N2O	0	2	10	6	0	1	0	11	2	4	0.0556	416	359	274	0.7632	alzghoul2014	alzghoul2014	C1=CC(=C(C=C1Cl)Cl)COC(CN2C=CN=C2)C3=C(C=C(C=C3)Cl)Cl	7
Flurbiprofen	C15H13FO2	1	0	9	5	1	0	1	9	0	1	0.1333	244	388	267	0.6881	alzghoul2014	alzghoul2014	CC(C1=CC(=C(C=C1)C2=CC=CC=C2)F)C(=O)O	7
Fenofibrate	C20H21ClO4	4	0	9	7	0	2	2	10	0	1	0.2000	361	354	254	0.7175	alzghoul2014	alzghoul2014	CC(C)OC(=O)C(C)(C)OC1=CC=C(C=C1)C(=O)C2=CC=C(C=C2)Cl	7

Abbreviation	Reference	Link
crchandbook	Haynes, W. M. (2014). CRC Handbook of Chemistry and Physics: A ready-reference book of chemical and physical data. Boca Raton, London, New York: CRC Press.	
bradleydataset	Bradley, J.-C., Lang, A., and Williams, A. (2014). Jean-Claude Bradley Double Plus Good (Highly Curated and Validated) Melting Point Dataset. Accessed November 02, 2022, https://dx.doi.org/10.6084/m9.figshare.1031638	https://dx.doi.org/10.6084/m9.figshare.1031638
yawshandbook	Yaws, C. L. (2015). The Yaws handbook of physical properties for hydrocarbons and chemicals: Physical properties for more than 54,000 organic and inorganic chemical compounds, coverage for C1 to C100 organics and Ac to Zr inorganics. Amsterdam, Boston, Heidelberg: Elsevier.	
römpp	Böckler, F., Dill, B., Eisenbrand, G., Faupel, F., Fugmann, B., Gamse, T., et al. (2022). RÖMPP Online-Enzyklopädie. Accessed November 02, 2022, https://roempp.thieme.de	https://roempp.thieme.de
mellan62	Mellan, I. (1962). Polyhydric alcohols. Washington: Spartan Books.	
hmdb	Wishart, D. S., Guo, A., Oler, E., Wang, F., Anjum, A., Peters, H., et al. (2022). HMDB 5.0: the Human Metabolome Database for 2022. Nucleic Acids Res 50, D622-D631. doi: 10.1093/nar/gkab1062	https://hmdb.ca/metabolites/HMDB0029933
SimonBA	John, S. (2017). Entwicklung von Regressions- und Prognosemethoden zur Beschreibung von Glasübergangstemperaturen in ternären Mischungen. Bachelor Thesis. Bielefeld: Bielefeld University, Faculty of Chemistry.	
maltini97	Maltini, E., Anese, M., and Shtylla, I. (1997). State diagrams of some organic acid-water systems of interest in food. Cryo-Letters 18, 263–268.	
o'donnell2012	O'Donnell, K., and Kearsley, M. W., eds (2012). Sweeteners and Sugar Alternatives in Food Technology. Chichester: Wiley.	
faucher66	Faucher, J. A., and Koleske, J. V. (1966). Glass Transitions of Organic Compounds. 1. Lower Aliphatic Alcohols. Physics and Chemistry of Glasses 7, 202.	
petrov1959	Petrov, A. A., Sergienko, S. R., Nechitailo, N. A., and Tsedilina, A. L. (1959). Synthesis and properties of C12- C16 monomethylalkanes. Russ Chem Bull 8, 1053–1058. doi: 10.1007/BF00916675	https://doi.org/10.1007/BF00916675
yamauchi61	Yamauchi, F., and Aso, K. (1961). Crystalline alpha-Kojibiose. Nature 189, 753. doi: 10.1038/189753a0	https://doi.org/10.1038/189753a0
stodola56	Stodola, F. H., Sharpe, E. S., and Koepsell, H. J. (1956). The Preparation, Properties and Structure of the Disaccharide Leucrose. J. Am. Chem. Soc. 78, 2514–2518. doi: 10.1021/ja01592a050	https://doi.org/10.1021/ja01592a050
lei2022	Lei, Z., Zhang, J., Mueller, E. A., Xiao, Y., Kolozsvari, K. R., McNeil, A. J., et al. (2022). Glass Transition Temperatures of Individual Submicrometer Atmospheric Particles: Direct Measurement via Heated Atomic Force Microscopy Probe. Anal Chem 94, 11973–11977. doi: 10.1021/acs.analchem.2c01979	https://doi.org/10.1021/acs.analchem.2c01979
kercthermochimacta95	Keřc, J., and Sřcič, S. (1995). Thermal analysis of glassy pharmaceuticals. Thermochimica Acta 248, 81–95. doi: 10.1016/0040-6031(94)01949-H	https://doi.org/10.1016/0040-6031(94)01949-H
beamanjpolysci52	Beaman, R. G. (1952). Relation between (apparent) second-order transition temperature and melting point. J. Polym. Sci. 9, 470–472. doi: 10.1002/pol.1952.120090510	https://doi.org/10.1002/pol.1952.120090510
murthyjchemsocfaradaytrans93	Murthy, S. S. N., and Kumar, D. (1993). Glass formation in organic binary liquids studied using differential scanning calorimetry. Faraday Trans. 89, 2423. doi: 10.1039/FT9938902423	https://doi.org/10.1039/FT9938902423
wangjchemphys06	Wang, L.-M., Angell, C. A., and Richert, R. (2006). Fragility and thermodynamics in nonpolymeric glass-forming liquids. Journal of Chemical Physics 125, 74505. doi: 10.1063/1.2244551	https://doi.org/10.1063/1.2244551
naitojphychem93	Naito, K., and Miura, A. (1993). Molecular design for nonpolymeric organic dye glasses with thermal stability: relations between thermodynamic parameters and amorphous properties. J. Phys. Chem. 97, 6240–6248. doi: 10.1021/j100125a025	https://doi.org/10.1021/j100125a025
koop2011	Koop, T., Bookhold, J., Shiraiwa, M., and Pöschl, U. (2011). Glass transition and phase state of organic compounds: dependency on molecular properties and implications for secondary organic aerosols in the atmosphere. Phys Chem Chem Phys 13, 19238–19255. doi: 10.1039/C1CP22617G	https://doi.org/10.1039/C1CP22617G
carpenterjchemphys67	Carpenter, M. R., Davies, D. B., and Matheson, A. J. (1967). Measurement of the Glass-Transition Temperature of Simple Liquids. Journal of Chemical Physics 46, 2451–2454. doi: 10.1063/1.1841068	https://doi.org/10.1063/1.1841068
lesikarjchemphys75	Lesikar, A. V. (1975). On the glass transition in organic halide–alcohol mixtures. Journal of Chemical Physics 63, 2297–2302. doi: 10.1063/1.431680	https://doi.org/10.1063/1.431680
lesikar_jsolchem_77	Lesikar, A. V. (1977). On the self-association of the normal alcohols and the glass transition in alcohol-alcohol solutions. J Solution Chem 6, 81–93. doi: 10.1007/BF00643434	https://doi.org/10.1007/BF00643434
lesikarjchemphys77	Lesikar, A. V. (1977). On the glass transition in mixtures between the normal alcohols and various Lewis bases. Journal of Chemical Physics 66, 4263–4276. doi: 10.1063/1.433736	https://doi.org/10.1063/1.433736
koleskepolymengsci79	Koleske, J. V., and Faucher, J. A. (1979). Glass transitions of organic compounds. III. Cellulose substrate technique and aliphatic alcohols. Polym. Eng. Sci. 19, 716–721. doi: 10.1002/pen.760191011	https://doi.org/10.1002/pen.760191011
johariannnyacadsci76	Johari, G. P. (1976). Glass Transition and Secondary Relaxations in Molecular Liquids and Crystals. Ann NY Acad Sci 279, 117–140. doi: 10.1111/j.1749-6632.1976.tb39701.x	https://doi.org/10.1111/j.1749-6632.1976.tb39701.x
nayakjchemphys93	Murthy, S. S. N., and Nayak, S. K. (1993). Experimental study of the nature of the glass transition process in monohydroxy alcohols. Journal of Chemical Physics 99, 5362–5368. doi: 10.1063/1.466187	https://doi.org/10.1063/1.466187
wangjphyschemb07	Wang, L.-M., and Richert, R. (2007). Glass transition dynamics and boiling temperatures of molecular liquids and their isomers. Journal of Physical Chemistry B 111, 3201–3207. doi: 10.1021/jp0688254	https://doi.org/10.1021/jp0688254
ramos2014	Ramos, S. L. L. M., Ogino, M., and Oguni, M. (2014). Phase- and glass-transition phenomena due to the same configurational order–disorder mechanism in crystalline racemic sec-butylcyclohexane. J Therm Anal Calorim 115, 1353–1358. doi: 10.1007/s10973-013-3436-1	https://doi.org/10.1007/s10973-013-3436-1
tyllijmolecstruct90	Tylli, H., Konschin, H., and Tenhu, H. (1990). A Raman spectroscopic and differential scanning calorimetric study of the different crystalline phases of guaiacol. Journal of Molecular Structure 220, 129–136. doi: 10.1016/0022-2860(90)80105-S	https://doi.org/10.1016/0022-2860(90)80105-S
hancockjpharmsci97	Hancock, B. C., and Zografi, G. (1997). Characteristics and significance of the amorphous state in pharmaceutical systems. J Pharm Sci 86, 1–12. doi: 10.1021/js9601896	https://doi.org/10.1021/js9601896
tiersthermochimact93	v.d. Tiers, G., and Francis, C. V. (1993). Materials science of organic compounds. Thermochimica Acta 226, 311–316. doi: 10.1016/0040-6031(93)80232-Y	https://doi.org/10.1016/0040-6031(93)80232-Y
mariajthermanalcalorim10	Maria, T. M. R., Lopes Jesus, A. J., and Eusébio, M. E. S. (2010). Glass-forming ability of butanediol isomers. J Therm Anal Calorim 100, 385–390. doi: 10.1007/s10973-009-0633-z	https://doi.org/10.1007/s10973-009-0633-z
albasimionescojchemphys99	Alba-Simionesco, C., Fan, J., and Angell, C. A. (1999). Thermodynamic aspects of the glass transition phenomenon. II. Molecular liquids with variable interactions. Journal of Chemical Physics 110, 5262–5272. doi: 10.1063/1.478800	https://doi.org/10.1063/1.478800
fukuokachempharmbull91	Fukuoka, E., Makita, M., and Nakamura, Y. (1991). Glassy State of Pharmaceuticals. V. Relaxation during Cooling and Heating of Glass by Differential Scanning Calorimetry. Chem. Pharm. Bull. 39, 2087–2090. doi: 10.1248/cpb.39.2087	https://doi.org/10.1248/cpb.39.2087
wangjchemphys08	Wang, L.-M., Tian, Y., Liu, R., and Richert, R. (2008). Calorimetric versus kinetic glass transitions in viscous monohydroxy alcohols. Journal of Chemical Physics 128, 84503. doi: 10.1063/1.2840357	https://doi.org/10.1063/1.2840357
murthyjphychem96	Murthy, S. S. N. (1996). Dielectric Relaxation in Monohydroxy Alcohols and Its Connection to the Glass Transition Process. J. Phys. Chem. 100, 8508–8517. doi: 10.1021/jp953596z	https://doi.org/10.1021/jp953596z
kolodziejczyk19	Kołodziejczyk, A., Pyrcz, P., Pobudkowska, A., Błaziak, K., and Szmigielski, R. (2019). Physicochemical Properties of Pinic, Pinonic, Norpinic, and Norpinonic Acids as Relevant α-Pinene Oxidation Products. J Phys Chem B 123, 8261–8267. doi: 10.1021/acs.jpcb.9b05211	https://doi.org/10.1021/acs.jpcb.9b05211

Abbreviation	Reference	Link
imamura2006	Imamura, K., Sakaura, K., Ohyama, K., Fukushima, A., Imanaka, H., Sakiyama, T., et al. (2006). Temperature scanning FTIR analysis of hydrogen bonding states of various saccharides in amorphous matrixes below and above their glass transition temperatures. Journal of Physical Chemistry B 110, 15094–15099. doi: 10.1021/jp057527o	https://doi.org/10.1021/jp057527o
orford89	Orford, P. D., Parker, R., Ring, S. G., and Smith, A. C. (1989). Effect of water as a diluent on the glass transition behaviour of malto-oligosaccharides, amylose and amylopectin. International Journal of Biological Macromolecules 11, 91–96. doi: 10.1016/0141-8130(89)90048-2	https://doi.org/10.1016/0141-8130(89)90048-2
curtin2013	Curtin, V., Amharar, Y., Hu, Y., Erxleben, A., McArdle, P., Caron, V., et al. (2013). Investigation of the capacity of low glass transition temperature excipients to minimize amorphization of sulfadimidine on comilling. Mol Pharm 10, 386–396. doi: 10.1021/mp300529a	https://doi.org/10.1021/mp300529a
zhang2019	Zhang, Y., Nichman, L., Spencer, P., Jung, J. I., Lee, A., Heffernan, B. K., et al. (2019). The Cooling Rate- and Volatility-Dependent Glass-Forming Properties of Organic Aerosols Measured by Broadband Dielectric Spectroscopy. Environ Sci Technol 53, 12366–12378. doi: 10.1021/acs.est.9b03317	https://doi.org/10.1021/acs.est.9b03317
lessmeier18	Lessmeier, J., Dette, H. P., Godt, A., and Koop, T. (2018). Physical state of 2-methylbutane-1,2,3,4-tetraol in pure and internally mixed aerosols. Atmos. Chem. Phys. 18, 15841–15857. doi: 10.5194/acp-18-15841-2018	https://doi.org/10.5194/acp-18-15841-2018
johari71	Johari, G. P., and Goldstein, M. (1971). Viscous Liquids and the Glass Transition. III. Secondary Relaxations in Aliphatic Alcohols and Other Nonrigid Molecules. Journal of Chemical Physics 55, 4245–4252. doi: 10.1063/1.1676742	https://doi.org/10.1063/1.1676742
talja2001	Talja, R. A., and Roos, Y. H. (2001). Phase and state transition effects on dielectric, mechanical, and thermal properties of polyols. Thermochimica Acta 380, 109–121. doi: 10.1016/S0040-6031(01)00664-5	https://doi.org/10.1016/S0040-6031(01)00664-5
nakanishi2011	Nakanishi, M., and Nozaki, R. (2011). Systematic study of the glass transition in polyhydric alcohols. Phys Rev E Stat Nonlin Soft Matter Phys 83, 51503. doi: 10.1103/PhysRevE.83.051503	https://doi.org/10.1103/PhysRevE.83.051503
dorfmueller79	Dorfmüller, T., Dux, H., Fytas, G., and Mersch, W. (1979). A light scattering study of the molecular motion in hexanetriol 1,2,6. Journal of Chemical Physics 71, 366–375. doi: 10.1063/1.438079	https://doi.org/10.1063/1.438079
angell82	Angell, C. A., Stell, R. C., and Sichina, W. (1982). Viscosity-temperature function for sorbitol from combined viscosity and differential scanning calorimetry studies. J. Phys. Chem. 86, 1540–1542. doi: 10.1021/j100206a015	https://doi.org/10.1021/j100206a015
yu98	Yu, L., Mishra, D. S., and Rigsbee, D. R. (1998). Determination of the glass properties of D-mannitol using sorbitol as an impurity. J Pharm Sci 87, 774–777. doi: 10.1021/js970224o	https://doi.org/10.1021/js970224o
jabrane98	Jabrane, S., Létoffé, J., and Claudy, P. (1998). Study of the thermal behaviour of 1,3-propanediol and its aqueous solutions. Thermochimica Acta 311, 121–127. doi: 10.1016/S0040-6031(97)00416-4	https://doi.org/10.1016/S0040-6031(97)00416-4
xia2006	Xia, Y., Dosseh, G., Morineau, D., and Alba-Simionesco, C. (2006). Phase diagram and glass transition of confined benzene. Journal of Physical Chemistry B 110, 19735–19744. doi: 10.1021/jp063393i	https://doi.org/10.1021/jp063393i
takeda98	Takeda, K., Murata, K., and Yamashita, S. (1998). Thermodynamic investigation of glass transition in binary polyalcohols. Journal of Non-Crystalline Solids 231, 273–279. doi: 10.1016/S0022-3093(98)00538-9	https://doi.org/10.1016/S0022-3093(98)00538-9
carpentier2003	Carpentier, L., Desprez, S., and Descamps, M. (2003). Crystallization and glass properties of pentitols. J Therm Anal Calorim 73, 577–586. doi: 10.1023/A:1025482230325	https://doi.org/10.1023/A:1025482230325
roos93	Roos, Y. H. (1993). Melting and glass transitions of low molecular weight carbohydrates. Carbohydrate Research 238, 39–48. doi: 10.1016/0008-6215(93)87004-C	https://doi.org/10.1016/0008-6215(93)87004-C
orford90	Orford, P. D., Parker, R., and Ring, S. G. (1990). Aspects of the glass transition behaviour of mixtures of carbohydrates of low molecular weight. Carbohydrate Research 196, 11–18. doi: 10.1016/0008-6215(90)84102-Z	https://doi.org/10.1016/0008-6215(90)84102-Z
slade94	Slade, L., and Levine, H. (1995). Water and the glass transition — Dependence of the glass transition on composition and chemical structure: Special implications for flour functionality in cookie baking. Journal of Food Engineering 24, 431–509. doi: 10.1016/0260-8774(95)90766-5	https://doi.org/10.1016/0260-8774(95)90766-5
pinto2010	Pinto, S. S., Diogo, H. P., Nunes, T. G., and Moura Ramos, J. J. (2010). Molecular mobility studies on the amorphous state of disaccharides. I-thermally stimulated currents and differential scanning calorimetry. Carbohydrate Research 345, 1802–1807. doi: 10.1016/j.carres.2010.05.023	https://doi.org/10.1016/j.carres.2010.05.023
miller2000	Miller, D. P., and Pablo, J. J. de (2000). Calorimetric Solution Properties of Simple Saccharides and Their Significance for the Stabilization of Biological Structure and Function. Journal of Physical Chemistry B 104, 8876–8883. doi: 10.1021/jp000807d	https://doi.org/10.1021/jp000807d
wungtanagorn2001	Wungtanagorn, R., and Schmidt, S. J. (2001). Phenomenological study of enthalpy relaxation of amorphous glucose, fructose, and their mixture. Thermochimica Acta 369, 95–116. doi: 10.1016/S0040-6031(00)00741-3	https://doi.org/10.1016/S0040-6031(00)00741-3
shamblin99	Shamblin, S. L., Tang, X. L., Chang, L. Q., Hancock, B. C., and Pikal, M. J. (1999). Characterization of the time scales of molecular motion in pharmaceutically important glasses. Journal of Physical Chemistry B 103, 4113–4121. doi: 10.1021/jp983964+	https://doi.org/10.1021/jp983964+
saleki-gerhardt94	Saleki-Gerhardt, A., and Zografi, G. (1994). Non-isothermal and isothermal crystallization of sucrose from the amorphous state. Pharm Res 11, 1166–1173. doi: 10.1023/A:1018945117471	https://doi.org/10.1023/A:1018945117471
taylor98	Taylor, L. S., and Zografi, G. (1998). Sugar-polymer hydrogen bond interactions in lyophilized amorphous mixtures. J Pharm Sci 87, 1615–1621. doi: 10.1021/js9800174	https://doi.org/10.1021/js9800174
elamin95	Elamin, A. (1995). The use of amorphous model substances to study mechanically activated materials in the solid state. International Journal of Pharmaceutics 119, 25–36. doi: 10.1016/0378-5173(94)00364-B	https://doi.org/10.1016/0378-5173(94)00364-B
tombari2015	Tombari, E., and Johari, G. P. (2015). Structural fluctuations and orientational glass of levoglucosan--High stability against ordering and absence of structural glass. J Chem Phys 142, 104501. doi: 10.1063/1.4913759	https://doi.org/10.1063/1.4913759
lienhard2012	Lienhard, D. M., Zobrist, B., Zuend, A., Krieger, U. K., and Peter, T. (2012). Experimental evidence for excess entropy discontinuities in glass-forming solutions. J Chem Phys 136, 74515. doi: 10.1063/1.3685902	https://doi.org/10.1063/1.3685902
singh2011	Singh, L. P., Alegría, A., and Colmenero, J. (2011). Broadband dielectric spectroscopy and calorimetric investigations of D-lyxose. Carbohydrate Research 346, 2165–2172. doi: 10.1016/j.carres.2011.06.029	https://doi.org/10.1016/j.carres.2011.06.029
kawai2005	Kawai, K., Hagiwara, T., Takai, R., and Suzuki, T. (2005). Comparative investigation by two analytical approaches of enthalpy relaxation for glassy glucose, sucrose, maltose, and trehalose. Pharm Res 22, 490–495. doi: 10.1007/s11095-004-1887-6	https://doi.org/10.1007/s11095-004-1887-6
kajiwara97	Kajiwara, K., and Franks, F. (1997). Crystalline and amorphous phases in the binary system water–raffinose. Faraday Trans. 93, 1779–1783. doi: 10.1039/A608572E	https://doi.org/10.1039/A608572E
buitink2000	Buitink, J., van den Dries, I. J., Hoekstra, F. A., Alberda, M., and Hemminga, M. A. (2000). High Critical Temperature above Tg May Contribute to the Stability of Biological Systems. Biophysical Journal 79, 1119–1128. doi: 10.1016/S0006-3495(00)76365-X	https://doi.org/10.1016/S0006-3495(00)76365-X
moura_ramos2005	Moura Ramos, J. J., Pinto, S. S., and Diogo, H. P. (2005). Molecular mobility in raffinose in the crystalline pentahydrate form and in the amorphous anhydrous form. Pharm Res 22, 1142–1148. doi: 10.1007/s11095-005-5645-1	https://doi.org/10.1007/s11095-005-5645-1

Abbreviation	Reference	Link
simperler2006	Simperler, A., Kornherr, A., Chopra, R., Bonnet, P. A., Jones, W., Motherwell, W. D. S., et al. (2006). Glass transition temperature of glucose, sucrose, and trehalose: an experimental and in silico study. Journal of Physical Chemistry B 110, 19678–19684. doi: 10.1021/jp063134t	https://doi.org/10.1021/jp063134t
crowe96	Crowe, L. M., Reid, D. S., and Crowe, J. H. (1996). Is trehalose special for preserving dry biomaterials? Biophysical Journal 71, 2087–2093. doi: 10.1016/S0006-3495(96)79407-9	https://doi.org/10.1016/S0006-3495(96)79407-9
miller97	Miller, D. P., Pablo, J. J. de, and Corti, H. (1997). Thermophysical properties of trehalose and its concentrated aqueous solutions. Pharm Res 14, 578–590. doi: 10.1023/A:1012192725996	https://doi.org/10.1023/A:1012192725996
lu97	Lu, Q., and Zografi, G. (1997). Properties of citric acid at the glass transition. J Pharm Sci 86, 1374–1378. doi: 10.1021/js970157y	https://doi.org/10.1021/js970157y
hoppu2009	Hoppu, P., Hietala, S., Schantz, S., and Juppo, A. M. (2009). Rheology and molecular mobility of amorphous blends of citric acid and paracetamol. Eur J Pharm Biopharm 71, 55–63. doi: 10.1016/j.ejpb.2008.06.029	https://doi.org/10.1016/j.ejpb.2008.06.029
timko79	Timko, R. J., and Lordi, N. G. (1979). Thermal characterization of citric acid solid dispersions with benzoic acid and phenobarbital. J Pharm Sci 68, 601–605. doi: 10.1002/jps.2600680523	https://doi.org/10.1002/jps.2600680523
dette2014	Dette, H. P., Qi, M., Schröder, D. C., Godt, A., and Koop, T. (2014). Glass-forming properties of 3-methylbutane-1,2,3-tricarboxylic acid and its mixtures with water and pinonic acid. J Phys Chem A 118, 7024–7033. doi: 10.1021/jp505910w	https://doi.org/10.1021/jp505910w
yamamuro99	Takahara, S., Yamamuro, O., and Suga, H. (1994). Heat capacities and glass transitions of 1-propanol and 3-methylpentane under pressure. New evidence for the entropy theory. Journal of Non-Crystalline Solids 171, 259–270. doi: 10.1016/0022-3093(94)90195-3	https://doi.org/10.1016/0022-3093(94)90195-3
alba90	Alba, C., Busse, L. E., List, D. J., and Angell, C. A. (1990). Thermodynamic aspects of the vitrification of toluene, and xylene isomers, and the fragility of liquid hydrocarbons. Journal of Chemical Physics 92, 617–624. doi: 10.1063/1.458411	https://doi.org/10.1063/1.458411
angell78	Angell, C. A., Sare, J. M., and Sare, E. J. (1978). Glass transition temperatures for simple molecular liquids and their binary solutions. J. Phys. Chem. 82, 2622–2629. doi: 10.1021/j100513a016	https://doi.org/10.1021/j100513a016
alzghoul2014	Alhalaweh, A., Alzghoul, A., Kaialy, W., Mahlin, D., and Bergström, C. A. S. (2014). Computational Predictions of Glass-Forming Ability and Crystallization Tendency of Drug Molecules. Mol Pharm 11, 3123–3132. doi: 10.1021/mp500303a	https://doi.org/10.1021/mp500303a
engineeringtoolbox	Engineering ToolBox (2001). Accessed May 15, 2021, https://www.engineeringtoolbox.com	https://www.engineeringtoolbox.com/
chembook1	ChemicalBook Inc. 1,2,6-Hexanetriol. Accessed September 09, 2022, https://www.chemicalbook.com/ChemicalProductProperty_US_CB6193771.aspx	https://www.chemicalbook.com/ChemicalProductProperty_US_CB6193771.aspx
chembook2	ChemicalBook Inc. Maltohexaose. Accessed October 13, 2022, https://www.chemicalbook.com/ProductChemicalPropertiesCB5222922_EN.htm	https://m.chemicalbook.com/ProductChemicalPropertiesCB5222922_EN.htm
carboExpert	Carboexpert Inc. Maltopentaose. Accessed October 13, 2022, https://www.carboexpert.com/wp-content/uploads/2018/01/MSDS-Maltopentaose-1.pdf	https://www.carboexpert.com/product/maltopentaose/
gestis2	Institut für Arbeitsschutz der Deutschen Gesetzlichen Unfallversicherung. Isoeugenol. Accessed September 12, 2022, https://gestis.dguv.de/data?name=492574	https://gestis.dguv.de/data?name=492574
gestis3	Institut für Arbeitsschutz der Deutschen Gesetzlichen Unfallversicherung. cis-2-Hepten. Accessed September 12, 2022, https://gestis.dguv.de/data?name=120579	https://gestis.dguv.de/data?name=120579
gestis4	Institut für Arbeitsschutz der Deutschen Gesetzlichen Unfallversicherung. 3-Hexanol. Accessed September 12, 2022, https://gestis.dguv.de/data?name=491019	https://gestis.dguv.de/data?name=491019
gestis5	Institut für Arbeitsschutz der Deutschen Gesetzlichen Unfallversicherung. 3-Heptanol. Accessed September 12, 2022, https://gestis.dguv.de/data?name=570042	https://gestis.dguv.de/data?name=570042
gestis6	Institut für Arbeitsschutz der Deutschen Gesetzlichen Unfallversicherung. 1,2,4-Butantriol. Accessed September 12, 2022, https://gestis.dguv.de/data?name=114088	https://gestis.dguv.de/data?name=114088
gestis7	Institut für Arbeitsschutz der Deutschen Gesetzlichen Unfallversicherung. Benzol. Accessed September 12, 2022, https://gestis.dguv.de/data?name=010060	https://gestis.dguv.de/data?name=010060
trccanada	Toronto Research Chemicals. Safety Data Sheet Maltotetraose. Accessed September 13, 2022, https://www.trc-canada.com/prod-img/MSDS/M161520MSDS.pdf	https://www.trc-canada.com/product-detail/?M161520
